# Identifying plasma metabolic characteristics of major depressive disorder, bipolar disorder, and schizophrenia in adolescents

**DOI:** 10.1038/s41398-024-02886-z

**Published:** 2024-03-26

**Authors:** Bangmin Yin, Yuping Cai, Teng Teng, Xiaolin Wang, Xueer Liu, Xuemei Li, Jie Wang, Hongyan Wu, Yuqian He, Fandong Ren, Tianzhang Kou, Zheng-Jiang Zhu, Xinyu Zhou

**Affiliations:** 1 Department of Psychiatry, The First Affiliated Hospital of Chongqing Medical University, Chongqing, China; 2 Interdisciplinary Research Center on Biology and Chemistry, Shanghai Institute of Organic Chemistry, Chinese Academy of Sciences, Shanghai, China; 3 Health Management Center, The First Affiliated Hospital of Chongqing Medical University, Chongqing, China; 4 Department of Neurology, The First Affiliated Hospital of Chongqing Medical University, Chongqing, China; 5 Shanghai Key Laboratory of Aging Studies, Shanghai, China

**Keywords:** Bipolar disorder, Depression, Schizophrenia

## Abstract

Major depressive disorder (MDD), bipolar disorder (BD), and schizophrenia (SCZ) are classified as major mental disorders and together account for the second-highest global disease burden, and half of these patients experience symptom onset in adolescence. Several studies have reported both similar and unique features regarding the risk factors and clinical symptoms of these three disorders. However, it is still unclear whether these disorders have similar or unique metabolic characteristics in adolescents. We conducted a metabolomics analysis of plasma samples from adolescent healthy controls (HCs) and patients with MDD, BD, and SCZ. We identified differentially expressed metabolites between patients and HCs. Based on the differentially expressed metabolites, correlation analysis, metabolic pathway analysis, and potential diagnostic biomarker identification were conducted for disorders and HCs. Our results showed significant changes in plasma metabolism between patients with these mental disorders and HCs; the most distinct changes were observed in SCZ patients. Moreover, the metabolic differences in BD patients shared features with those in both MDD and SCZ, although the BD metabolic profile was closer to that of MDD than to SCZ. Additionally, we identified the metabolites responsible for the similar and unique metabolic characteristics in multiple metabolic pathways. The similar significant differences among the three disorders were found in fatty acid, steroid-hormone, purine, nicotinate, glutamate, tryptophan, arginine, and proline metabolism. Interestingly, we found unique characteristics of significantly altered glycolysis, glycerophospholipid, and sphingolipid metabolism in SCZ; lysine, cysteine, and methionine metabolism in MDD and BD; and phenylalanine, tyrosine, and aspartate metabolism in SCZ and BD. Finally, we identified five panels of potential diagnostic biomarkers for MDD-HC, BD-HC, SCZ-HC, MDD-SCZ, and BD-SCZ comparisons. Our findings suggest that metabolic characteristics in plasma vary across psychiatric disorders and that critical metabolites provide new clues regarding molecular mechanisms in these three psychiatric disorders.

## Introduction

Major depressive disorder (MDD), bipolar disorder (BD), and schizophrenia (SCZ) are classified as major mental disorders and together account for the second-highest global disease burden, with ~55 million years lived with disability [1]. Approximately half of these patients experience emotional disturbance and functional impairment due to these disorders before the age of 15 years [2, 3]. According to the Diagnostic and Statistical Manual of Mental Disorders (DSM) and International Classification of Diseases codes, MDD, BD, and SCZ are diagnosed based on clinical features and symptoms rather than etiology [4]. Although MDD, BD, and SCZ are distinct disorders, they share some risk factors and clinical symptoms [5, 6]. Due to the overlap in these disorders, current diagnostic criteria based solely on symptoms cannot completely capture the essence of these major mental disorders, which hinders the selection of appropriate treatment strategies [7]. Additionally, the initial clinical manifestations in adolescence are often incomplete, variable, and uncharacteristic, which further contributes to heterogeneity in these disorders, particularly compared to those in adulthood [8, 9]. This heterogeneity is likely to weaken the validity of disease diagnosis and lead to obstacles and setbacks in clinical practice for adolescents [10].

To address the challenges posed by the heterogeneity of these major mental disorders, several studies have been published to explore their objective biological characteristics at the gene [5, 6], protein [11, 12], and metabolite levels [13]. Among these biological characteristics, metabolites provide a comprehensive functional representation of the biological process, and metabolomics has been extensively used to determine metabolic profiles, making this method one of the best tools for identifying disease-specific biomarkers and molecular mechanisms [14, 15]. Several previous metabolomics studies of major mental disorders have focused on metabolic characteristics of plasma in various psychiatric disorders. Oresic et al. used a metabolomics approach to analyze serum samples from adults with SCZ, affective disorders (MDD or BD), other non-affective psychoses, and healthy controls (HCs) and found that glucoregulatory processes were specifically associated with SCZ, and metabolic abnormalities were less pronounced in persons with affective disorders than in those with SCZ [13]. Tao et al. used a lipidomics approach to analyze serum samples from participants with MDD, BD, SCZ, and HCs and found that all patients could be divided into two clusters (one mainly including SCZ and BD, and the other mainly including MDD and BD) [16]. To our knowledge, no study has used a metabolomics approach to compare the plasma metabolic profiles of adolescent HCs and those of patients with MDD, BD, and SCZ, although the heterogeneity, functional impairment, and influence on later life of these major mental disorders are noteworthy in adolescents.

Given the significant heterogeneity, functional impairment, and influence on later life in adolescents, these major mental disorders are of substantial research interest. Thus, we conducted a comprehensive untargeted metabolomics analysis using hydrophilic interaction liquid chromatography-mass spectrometry and reversed-phase liquid chromatography-mass spectrometry on plasma samples from adolescents with MDD, BD, SCZ, and HCs. The objective of this study was to compare the plasma metabolism characteristics of adolescents with MDD, BD, SCZ, and HCs.

## Methods

### Participants and sample collection

All adolescent patients with major mental disorders (MDD, BD, and SCZ) were recruited from the Department of Psychiatry of The First Affiliated Hospital of Chongqing Medical University from December 2020 to August 2021. At the same time, population-based matched HCs were recruited from The First Affiliated Hospital of Chongqing Medical University by advertisements. The diagnosis of these major mental disorders was confirmed by two experienced psychiatrists according to the criteria in the Diagnostic and Statistical Manual of Mental Disorders, 5th edition (DSM-V). Participants were included in the study if they met the following criteria: (1) aged between 12 and 18 years, (2) diagnosed with MDD, BD, or SCZ (or healthy individuals matched to the disorder groups), and (3) provided informed consent (and their legal guardians also provided informed consent). Individuals were excluded if they met any of the following criteria: (1) history of head injury resulting in sustained loss of consciousness or cognitive sequela; (2) current or lifetime diagnosis of serious neurologic diseases or other psychiatric disorders such as epilepsy, encephalitis, autism, attention-deficit/hyperactivity disorder or obsessive-compulsive disorder; (3) presence of chronic physical diseases that could significantly affect peripheral metabolism or neurological function, such as hepatitis, chronic nephrosis, or chronic enteritis; or (4) history of substance abuse or dependence issues. Then, we collected general demographic and clinical characteristics, including sex, age, body mass index (BMI), duration of disease, drug therapy, and symptom scale. Clinical assessments of HCs and patients with BD or MDD were conducted using the 24-item Hamilton Depression Rating Scale (HAMD-24) and 14-item Hamilton Anxiety Rating Scale (HAMA-14). The Young Mania Rating Scale (YMRS) was used to assess mania symptoms in patients with BD. We characterized the clinical status of patients with SCZ using the Positive and Negative Syndrome Scale (PANSS) [17]. The dietary habits of the participants were assessed using a simplified dietary questionnaire, which included inquiries about the frequency of consuming carbohydrates, vegetables, fruits, meats, tea, and coffee on a weekly basis. Both participants and their legal guardians read and signed the informed consent form before participating. The protocol for this study was approved by the Ethics Committee of The First Affiliated Hospital of Chongqing Medical University (Approved ID: 2020-864).

All fasting blood samples were collected from the participants between 8:00 A.M. and 12:00 P.M./noon via venipuncture and were placed in EDTA-coated (anticoagulant) tubes. The tubes were then centrifuged for 10 min at 3000 rpm and 4 °C. After centrifugation, the plasma was separated and stored at −80 °C until metabolomics profiling.

### LC-MS analysis and metabolomics data processing

The LC-MS analysis protocol followed that in our previous publication [18]. Data acquisition was performed using a Thermo Scientific Vanquish UHPLC system coupled to a Thermo Scientific Orbitrap Exploris 480. All samples were randomly injected during data acquisition.

The data acquisition was operated in full MS-scan mode with a positive/negative ion polarity switch for individual samples. Information-dependent acquisition mode was used for quality control (QC) samples to acquire MS/MS spectra. The MS/MS spectra of the QC sample were acquired under a stepped normalized collision energy of 20–30–40%.

The data processing protocol followed that in our previous publication [19, 20]. Briefly, ProteoWizard (version 3.0.20360) was first used to convert raw MS data (.raw) files to the mzXML format. Then, mzXML data files of samples were grouped for peak detection, retention time correction, and peak alignment. Then, missing value imputation and data normalization were conducted. Metabolic peaks with relative standard deviations (RSDs) less than 30% in QC samples were used for subsequent analysis. Metabolite annotation was performed using MetDNA (version 1.2.2; http://metdna.zhulab.cn/). We performed metabolite annotation separately in both positive and negative modes. According to the definition of the metabolomics standards initiative, we assigned the metabolite annotations with three confidence levels. Level 1 was used for metabolites annotated through matching of MS1, RT, and MS/MS spectra with the in-house metabolite spectral library. Level 2 was used for metabolites annotated by matching MS1 and MS/MS2 spec with a public metabolite spectral library (mainly from NIST 2017). Level 3 was used for metabolites annotated based on MS1 and surrogate MS/MS match using MetDNA. The details of LC-MS analysis and metabolomics data processing were available in Supplementary Materials.

### Identifying differentially expressed metabolites between patients with these disorders and HCs

**Fig. 1:**
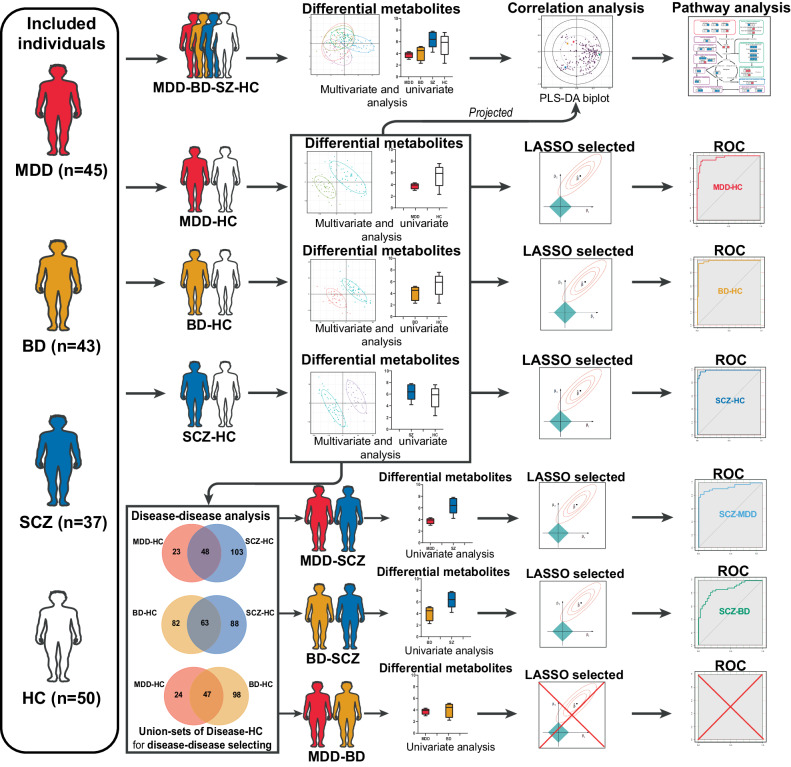
The flowchart of experimental process.

The experimental process is presented in Fig. 1 as a flowchart. To perform metabolomics analysis, we applied a combination of multivariate and univariate analyses to screen for differentially expressed metabolites from the MDD-BD-SCZ-HC comparison and disorder-HC comparisons (MDD-HC, BD-HC, and SCZ-HC). The multivariate analysis was conducted using R software (version 4.1.2) and SIMCA-P (version 11.0). First, we used an unsupervised principal component analysis (PCA) with unit variance scaling to evaluate the comprehensive metabolic alterations among groups. Then, supervised partial least-squares discrimination analysis (PLS-DA) with unit variance scaling was conducted to maximize the difference among groups and identify important metabolites with a quantitative contribution to the classification, according to their variable importance of projection (VIP) values [21]. To assess the risk of overfitting of the PLS-DA model, we performed a permutation test with 200 times. Univariate analysis was conducted using R software and SPSS (Statistical Product Service Solutions; version 20.0). For the univariate analysis, we used a Wilcoxon–Mann–Whitney test followed by false discovery rate (FDR) adjustment [22, 23] to determine the difference in every metabolite for the disorder-HC comparisons and a Kruskal–Wallis test followed by FDR adjustment to determine the difference in every metabolite for the MDD-BD-SCZ-HC comparison. Furthermore, the fold change (FC) in metabolites was calculated according to the ratio of the mean. Finally, differentially expressed metabolites were manually checked.

The criteria used to identify differentially expressed metabolites were as follows: VIP > 1, FDR-adjusted Wilcoxon–Mann‒Whitney test (for disorder-HC comparisons) or Kruskal‒Wallis test (for MDD-BD-SCZ-HC comparison) with *P* value < 0.05 and FC < 0.8 or >1.25 (for disorder-HC comparisons only). Volcano plots and heatmaps were generated using R to illustrate the global profile of the differentially expressed metabolites.

To investigate the potential impacts of confounding factors (drug treatment), all metabolites were subjected to Wilcoxon–Mann–Whitney tests followed by FDR adjustment for the MDD, BD, and SCZ groups. The influence of drug treatment was visualized using PCA plots for the MDD, BD, and SCZ groups. To investigate the potential impacts of confounding factors (dietary habit), all metabolites were subjected to Spearman correlation analysis followed by FDR adjustment for available information.

### Correlation and metabolic pathway analyses of groups and differentially expressed metabolites

To explore the correlation between differentially expressed metabolites and groups, we generated a biplot using a new PLS-DA model generated from the differentially expressed metabolites of the MDD-BD-SCZ-HC comparison. To assess the overfitting risk of this PLS-DA model, we performed a permutation test with 200 times. Then, to investigate how the differentially expressed metabolites from the three disorder-HC comparisons contributed to the four-group distinction, we projected the differentially expressed metabolites from each disorder-HC comparison onto the PLS-DA biplot. Finally, to further investigate the pathways of the differentially expressed metabolites that contributed to the classification of groups, we applied Kyoto Encyclopedia of Genes and Genomes (KEGG) pathway analysis to the collection of differentially expressed metabolites from the three disorder-HC comparisons projected onto the biplot as mentioned above.

### Identifying differentially expressed metabolites between patients with diverse disorders

The differentially expressed metabolites between diverse disorders were identified from union sets of differentially expressed metabolites from each two disease-HC comparisons (MDD-HC and SCZ-HC, MDD-HC and BD-HC, and SCZ-HC and BD-HC). The union sets were visualized by Venn diagrams. We compared metabolites of the union sets among the MDD-SCZ, MDD-BD, and BD-SCZ comparisons, respectively. The Wilcoxon–Mann–Whitney test was conducted to three union sets, followed by FDR adjustment. Differentially expressed metabolites between two disorders were identified based on FC values < 0.8 or >1.2 and FDR-adjusted *P* values < 0.05.

### Identifying potential diagnostic metabolites

Based on the package “glmnet” in R software, we used a tenfold cross-validation (CV) implementation of least absolute shrinkage and selection operator (LASSO) regression to select potential diagnostic metabolites from differentially expressed metabolites [24]. In this approach, each tenfold CV penalized regression model was trained. The penalized parameter lambda was selected as lambda.1se. We repeated this process ten times, resulting in ten different beta coefficients for each metabolite. Differentially expressed metabolites with nonzero beta coefficients generated across all ten LASSO models were selected as LASSO-selected metabolites.

We evaluated the discriminant validity of potential diagnostic metabolites (using only level 1 LASSO-selected metabolites) according to the receiver-operating characteristic (ROC) curve, which was generated from principal component regression (PCR) [25]. We performed PCR by determining the principal components (from PCA) and then using some of the principal components (exceeding a 90% contribution rate) as predictors in a linear regression model fitted with the typical least-squares procedure [26]. Then, the principal components were linearly transformed to potential diagnostic metabolites.

### Statistical analysis

The demographic and clinical characteristics of participants were analyzed using SPSS. The differences in gender were analyzed using the chi-square test, while the differences in age, BMI, HAMA score, HAMD score, and duration of illness were analyzed using the Kruskal‒Wallis test. All statistical tests in this study were two-sided. For metabolites with null values (NA), those with less than 50% null values were filled with half of the global minimum value, while those with more than 50% null values were deleted [27, 28].

## Results

### Demographic and clinical characteristics

Detailed information on the demographics and clinical characteristics of the recruited individuals is presented in Table 1. A total of 175 participants were enrolled, including 45 with MDD, 43 with BD, 37 with SCZ, and 50 HCs. The age of the participants ranged from 12 to 18 years. There were no significant differences in age (*P* = 0.178), gender (*P* = 0.058), or BMI (*P* = 0.696) among the four groups. The mean illness durations for patients with MDD, BP, and SCZ were 23.5 ± 20.3, 20.3 ± 18.8, and 15.9 ± 16.1 months, respectively, and there were no significant differences among the major mental disorders (*P* = 0.189). There were significant differences in HAMD and HAMA scores among the MDD, BD, and HC groups (*P* < 0.001). The HAMD scores for the MDD, BD, and HC groups were 26.5 ± 8.0, 25.9 ± 9.9, and 2.1 ± 1.7, respectively. The HAMA scores for the MDD, BD, and HC groups were 16.7 ± 8.0, 17.7 ± 6.4, and 1.4 ± 1.3, respectively. The PANSS score for patients with SCZ was 86.1 ± 20.3, and the YMRS score for patients with BD was 12.5 ± 8.1. The most commonly used drug in MDD group is sertraline (12/22). In BD group, the most commonly used drug is quetiapine (10/30). Olanzapine (14/27) is the most frequently used drug in SCZ group. Detailed medication information for patients in the three disease groups is provided in Supplementary Table 7. In the end, 59.4% (104/175) participants completed the dietary questionnaire, among whom 26 HC, 27 MDD, 32 BD, and 19 SCZ. And no significant differences in dietary habits were found among the four groups (Supplementary Table 8).

**Table 1:** Demographic characteristics of patients with MDD, BD, SCZ, and HCs.

	MDD (*n* = 45)	BD (*n* = 43)	SCZ (*n* = 37)	HCs (*n* = 50)	*P* value
Gender (male/female)^a^	10/35	8/35	15/22	19/31	0.058
Age (years)^b^	15.2 ± 1.5	15.7 ± 1.7	15.9 ± 1.9	15.4 ± 1.8	0.178
BMI^b^	20.4 ± 3.2	20.6 ± 3.2	20.4 ± 2.6	20.1 ± 3.8	0.696
Duration of disease (month)^b^	23.5 ± 20.3	20.3 ± 18.8	15.9 ± 16.1	NA	0.163
HAMD-24^b^	26.5 ± 8.0	25.9 ± 9.9	NA	2.1 ± 1.7	<0.001
HAMA-14^b^	16.7 ± 8.0	17.7 ± 6.4	NA	1.4 ± 1.3	<0.001
PANSS	NA	NA	86.1 ± 20.3	NA	NA
PANSS-P	NA	NA	21.5 ± 5.9	NA	NA
PANSS-N	NA	NA	18.8 ± 6.5	NA	NA
PANSS-G	NA	NA	38.2 ± 10.3	NA	NA
YMRS	NA	12.2 ± 8.1	NA	NA	NA
Drug therapy (yes/no)	22/23	30/13	27/10	0/50	NA

Continuous variables were presented as the mean ± SD(standard deviation). The differences in gender were analyzed using the chi-square test, while differences in age, BMI, HAMA, HAMD, and duration of disease were analyzed using the Kruskal–Wallis test.

*MDD* major depressive disorder, *BD* bipolar disorder, *SCZ* schizophrenia, *HC* health control, *BMI* body mass index, *HAMD-24* 24-item Hamilton depression rating scale, *HAMA-14* 14-item Hamilton anxiety rating scale, *PANSS* positive and negative syndrome scale, *YMRS* young mania rating scale.

^a^Analyzed by Chi-square test.

^b^Analyzed by Kruskal–Wallis test.

### Metabolic profiles and differentially expressed metabolites in the MDD-BD-SCZ-HC comparison

**Fig. 2:**
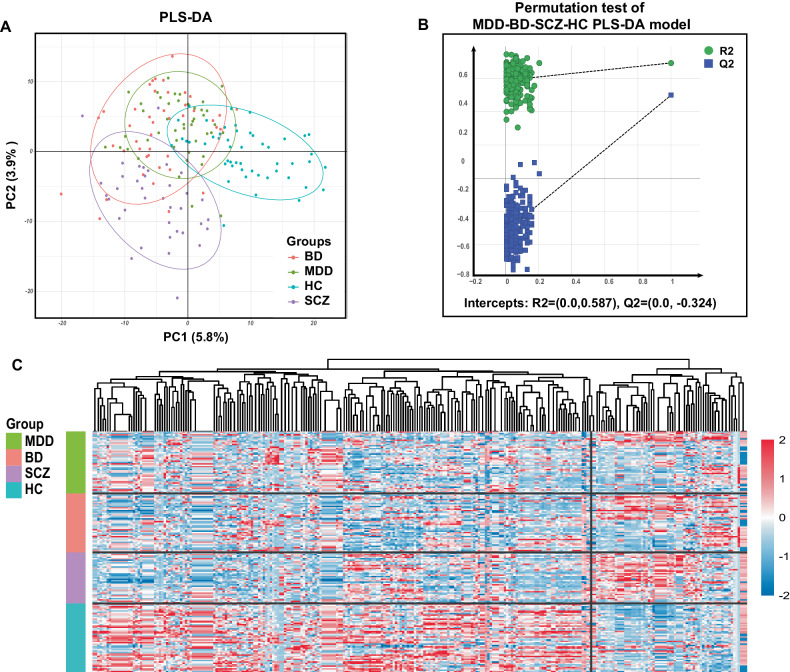
Metabolic profiles of the MDD-BD-SCZ-HC comparison. **A** The PLS-DA plot displays the distinguishing characteristics of the four groups. **B** The permutation test showed credible Q2 intercepts (0.00, −0.324). **C** The global profile of the 276 metabolites showed contrasts among patients with different mental disorders compared to HCs. Metabolites that were downregulated in the disorder groups clustered on the left of the heatmap, and those that were upregulated clustered on the right side. The most distinct contrast was observed between SCZ patients and HCs, while the least distinct contrast was observed between MDD patients and HCs.

The PCA plot showed a trend of differentiation, although with partial overlap, between HC and the three major mental disorders. (Supplementary Fig. 1A). Then, the supervised PLS-DA plot indicated more distinguishing characteristics, which showed that the HC group and the three disorder groups were excellently distinguished and that SCZ was well distinguished from MDD and BD, while the distinction between MDD and BD was relatively poor (Fig. 2A). The PLS-DA model had an acceptable goodness-of-fit (R2X = 0.179 and R2Y = 0.641) and predictability (Q2 = 0.280) (Supplementary Table 1). The permutation test showed credible Q2 intercepts (0.00, −0.324) (Fig. 2B). Finally, a total of 276 differentially expressed metabolites were identified in the MDD-BD-SCZ-HC comparison (VIP > 1, FDR < 0.05; Supplementary Table 2). The global profile of the 276 metabolites was visualized in a heatmap (Fig. 2C). The heatmap contrasts between the disorder groups and the HC group exhibited differential characteristics. The most distinct differences were found in SCZ, followed by BD with moderately distinct differences and MDD with minor differences (Fig. 2C). Additionally, we analyzed the impact of confounding factors (drug therapy) and found that there were no clear discrimination on the PCA plots of patients with MDD, BD, and SCZ for the drug-naïve and drug-treatment groups (Supplementary Fig. 2A–C). And no differentially expressed metabolites were identified for the drug-naïve and drug-treatment groups of patients with MDD, BD, or SCZ. Furthermore, no significant differences were found in correlation analyses between dietary habits and metabolites.

### Metabolic profiles and differentially expressed metabolites of the disorder-HC comparisons

**Fig. 3:**
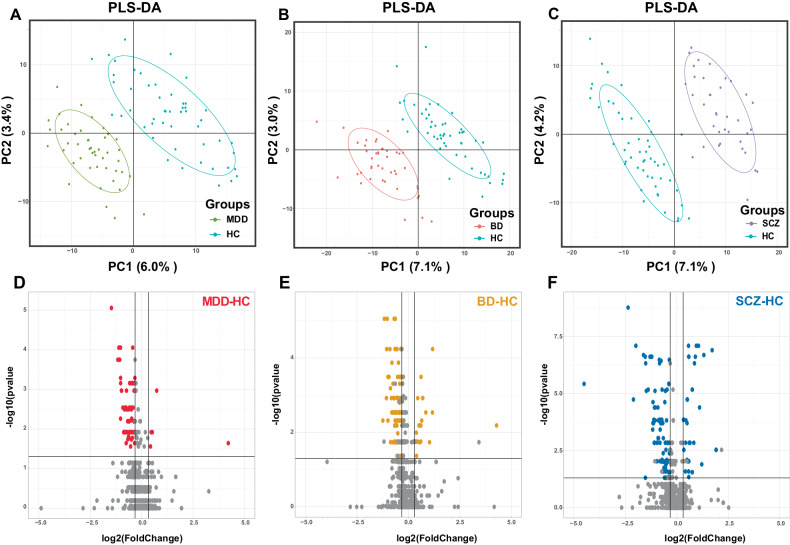
Metabolic profiles and differentially expressed metabolites of the disorder-HC comparisons. **A** The PLS-DA plot for the comparison between MDD and HC. **B** The PLS-DA plot for the comparison between BD and HC. **C** The PLS-DA plot for the comparison between SCZ and HC. **D** The Volcano plot for the comparison between MDD and HC showed the distribution of log_2_(FoldChange) and −log_10_(*P* value) of metabolites. The differentially expressed metabolites for MDD-HC were filled in red. **E** The Volcano plot for the comparison between BD and HC showed the distribution of log_2_(FoldChange) and −log_10_(*P* value) of metabolites. The differentially expressed metabolites for BD-HC were filled in orange. **F** The Volcano plot for the comparison between SCZ and HC showed the distribution of log_2_(FoldChange) and −log_10_(*P* value) of metabolites. The differentially expressed metabolites for SCZ-HC were filled in blue.

The PCA plots for the three disorder-HC comparisons (Supplementary Fig. 1B–D) showed a trend of differentiation between the disorder groups and the HC group. The supervised PLS-DA plots (Fig. 3A–C) demonstrated distinctions in the three disorder-HC comparisons. The three PLS-DA models had acceptable goodness-of-fit and predictability (Supplementary Table 1). The permutation tests of the three PLS-DA models are shown in Supplementary Fig. 3A–C. Based on the FC of metabolites, FDR-corrected *P* value, and VIP value (Fig. 3D–F), we identified 71 differentially expressed metabolites for the MDD-HC comparison, 145 differentially expressed metabolites for the BD-HC comparison, and 151 differentially expressed metabolites for the SCZ-HC comparison (Table 2). Detailed information on the differentially expressed metabolites for the disorder-HC comparisons (MDD-HC, BD-HC, and SCZ-HC) is listed in Supplementary Table 3A–C.

**Table 2:** Summary of differentially expressed metabolites and LASSO metabolites from the MDD-BD-SCZ-HC, disorder-HC, and disorder-disorder comparisons.

	HC-SCZ-BD-MDD	MDD-HC	BD-HC	SCZ-HC	MDD-SCZ	BD-SCZ	MDD-BD
Total differential metabolites	276	71	145	151	64	49	0
Level 1	137	45	61	86	37	26	0
Level 2	24	9	13	15	7	7	0
Level 3	115	17	71	50	20	16	0
Total LASSO metabolites	NA	14	10	13	13	10	NA
Level 1	NA	7*	7*	7*	8*	4*	NA
Level 2	NA	2	1	1	1	4	NA
Level 3	NA	5	2	5	4	2	NA

Level 1 metabolites marked with * are considered potential diagnostic metabolites for respective comparisons.

### Correlation analyses of groups and differentially expressed metabolites

**Fig. 4:**
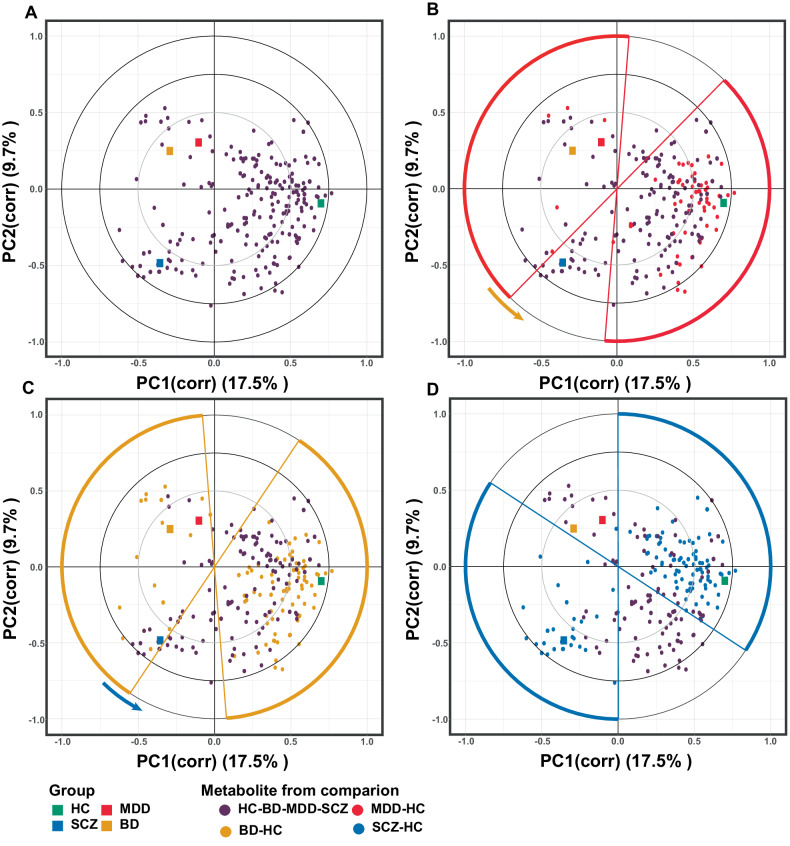
The PLS-DA biplot showed correlation between groups and differentially expressed metabolites. The PLS-DA biplot depicted the points of groups and metabolites on one coordinate system. Each point can be considered a vector from the origin to the point location. The correlation of vectors between two points can be represented as the projection of two vectors. **A** The PLS-DA biplot generated from the differentially expressed metabolites of the MDD-BD-SCZ-HC comparison showed the correlation between the four groups and the differentially expressed metabolites. **B** Differentially expressed metabolites from the MDD-HC comparison were projected onto PLS-DA biplot (**A**) and filled in red. The differentially expressed metabolites from the MDD-HC comparison were distributed in the 2nd and 4th quadrants and 1st and 3rd quadrants near the X-axis. **C** Differentially expressed metabolites from the BD-HC comparison were projected onto PLS-DA biplot (**A**) and filled in orange. The differentially expressed metabolites from the BD-HC comparison were distributed in the 2nd and 4th quadrants and 1st and 3rd quadrants near the X-axis. **D** Differentially expressed metabolites from the SCZ-HC comparison were projected onto PLS-DA biplot (**A**) and filled in blue. The differentially expressed metabolites from the SCZ-HC comparison were distributed in 1st and 3rd quadrants and 2nd and 4th quadrants near the X-axis.

The correlation between groups and differentially expressed metabolites was visualized using a new PLS-DA biplot (Fig. 4A). The biplot of the PLS-DA model had an acceptable goodness-of-fit (R2X = 0.340; R2Y = 0.355) and predictability (Q2 = 0.248) (Supplementary Table 1). The permutation test also showed credible Q2 intercepts (0.00, −0.171) (Supplementary Fig. 3D). The biplot showed the HC group in quadrant 4 (0.700, −0.090), the MDD and BD groups in quadrant 2 (MDD: −0.110, 0.300; BD: −0.290, 0.250), and the SCZ group in quadrant 3 (SCZ: −0.350, −0.490). The distribution of metabolites tended to cluster into three groups: the MDD and BD groups, the SCZ group, and the HC group.

To explore how differentially expressed metabolites from the three disorder-HC comparisons contributed to the four-group distinction, differentially expressed metabolites from each disorder-HC comparison were projected onto the PLS-DA biplot. Our results showed that the differentially expressed metabolites from the three disorder-HC comparisons were distributed in two opposite directions, with downregulated metabolites in the 1st and 4th quadrants (toward the HC group) and upregulated metabolites in the 2nd and 3rd quadrants (away from the HC group). Interestingly, there was a gradual counterclockwise shift in the distribution from the MDD-HC comparison to the BD-HC comparison and then to the SCZ-HC comparison in the coordinate system space, as shown by the arrows in Fig. 4B–D. This implied that the metabolic differences in BD shared characteristics of both MDD and SCZ and that the BD metabolic profile was more similar to that of MDD than to that of SCZ.

### Metabolic pathway analysis of differentially expressed metabolites between patients with major mental disorders and HCs

**Fig. 5:**
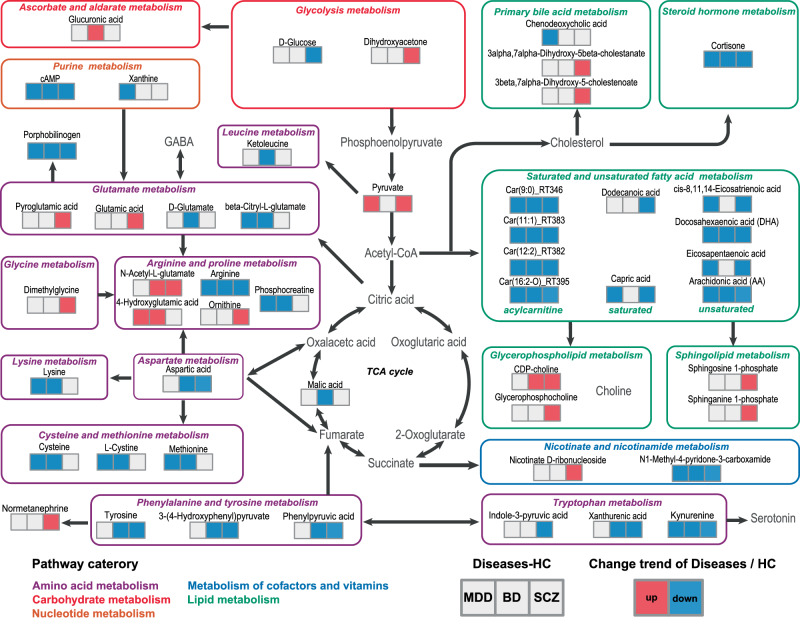
A simplified schematic of the altered metabolic pathways in the plasma of patients with MDD, BD and SCZ. For the differentially expressed metabolites, boxes in red represent upregulation, boxes in blue represent downregulation, and boxes in white represent no significant change when compared with expression in HCs. Each differentially expressed metabolite is associated with three horizontally arranged boxes, with the left box representing MDD, the middle box representing BD, and the right box representing SCZ. Notably, numerous acylcarnitines, intermediate products of fatty acid metabolism, were identified in all three comparisons (MDD-HC: 28 acylcarnitines, BD-HC: 18 acylcarnitines, and SCZ-HC: 36 acylcarnitines; Supplementary Table 4A–C). Given the similar biological function of acylcarnitines (and for simplicity of visualization), only four representative acylcarnitines are presented in this figure, selected based on their identification as level 1 potential diagnostic metabolites.

We identified a total of five metabolic pathway categories, including amino acid metabolism (glutamate, arginine, proline, leucine, glycine, aspartate, lysine, cysteine, methionine, phenylalanine, tyrosine, and tryptophan), lipid metabolism (saturated and unsaturated fatty acid, primary bile acid, steroid hormone, glycerophospholipid, and sphingolipid), nucleotide metabolism (purine), carbohydrate metabolism (glycolysis, ascorbate, and aldarate), and metabolism of cofactors and vitamins (nicotinate and nicotinamide). A total of 22 metabolites were classified into the amino acid metabolism pathway, 14 metabolites and four representative acylcarnitines were classified into the lipid metabolism pathway, three metabolites were classified into the carbohydrate metabolism pathway, two metabolites were classified into the nucleotide metabolism pathway, and two metabolites were classified into the pathway of metabolism of cofactors and vitamins. The detailed results of the metabolic pathways are visualized in Fig. 5.

Regarding metabolites of lipid metabolism, we found that unsaturated fatty acids, representative acylcarnitines, and cortisone were identified in all three disorder-HC comparisons. Glycerophospholipids and sphingolipids were mainly identified in the SCZ-HC comparison. Among the metabolites of amino acid metabolism, glutamate, tryptophan, arginine, and proline were identified in all three disorder-HC comparisons. Lysine, cysteine, and methionine were identified in the MDD-HC and BD-HC comparisons, whereas aspartate, phenylalanine, and tyrosine were identified in the BD-HC and SCZ-HC comparisons. In the metabolites of carbohydrate metabolism, d-glucose and dihydroxyacetone were identified in only the SCZ-HC comparison. In addition, purine and nicotinate metabolites were identified in all three disorder-HC comparisons.

### The differentially expressed metabolites between patients with diverse disorders

The union-set of MDD-HC and SCZ-HC included 174 metabolites (Supplementary Fig. 4A); the union-set of SCZ-HC and BD-HC included 233 metabolites (Supplementary Fig. 4B) and the union-set of MDD-HC and BD-HC included 169 metabolites (Supplementary Fig. 4C). Based on the FC of metabolites and FDR-corrected *P* value (Supplementary Fig. 4D–F), we identified 64 differentially expressed metabolites for the MDD-SCZ comparison, 49 differentially expressed metabolites for the BD-SCZ comparison (Table 2). However, no differentially expressed metabolite was identified from the MDD-BD comparison. Detailed information of the the union sets and the differentially expressed metabolites for the disorder-disorder comparisons (MDD-SCZ, BD-SCZ, and MDD-BD) is listed in Supplementary Table 5A–C.

### Identifying potential diagnostic metabolites

Finally, five panels of potential diagnostic biomarkers were identified for MDD-HC, BD-HC, SCZ-HC, MDD-SCZ, and BD-SCZ comparisons. The number and level of LASSO-selected metabolites of all comparisons are listed in Table 2. We identified seven potential diagnostic metabolites for the MDD-HC comparison; seven for the BD-HC comparison; seven for the SCZ-HC comparison; eight for the MDD-SCZ comparison and four for the BD-SCZ comparison. The areas under the ROC curves (AUCs) from each comparison were as follows: the MDD-HC comparison (AUC = 0.962, Supplementary Fig. 5A), the BD-HC comparison (AUC = 0.983, Supplementary Fig. 5B), the SCZ-HC comparison (AUC = 0.994, Supplementary Fig. 5C), the MDD-SCZ comparison (AUC = 0.933, Supplementary Fig. 4G) and the BD-SCZ comparison (AUC = 0.867, Supplementary Fig. 4H). The detailed parameters of LASSO and PCR models are listed in Supplementary Table 6. Additionally, in the process of missing value imputation, none of the potential diagnostic biomarkers had missing values imputed.

## Discussion

In our study, we first performed metabolomic analyses to compare plasma metabolism profiles among adolescents with MDD, BD, SCZ, and HCs. Our results showed significant differences in plasma metabolism between patients with these major mental disorders and HCs, with the most distinct differences observed in SCZ patients. Moreover, the metabolic differences between patients with BD and HCs had characteristics similar to those of both MDD and SCZ, and the BD metabolic profile was closer to that of MDD than to that of SCZ. Additionally, we found that fatty acid, steroid-hormone, purine, nicotinate, glutamate, tryptophan, arginine, and proline metabolism exhibited consistent significant differences among the three disorders. Interestingly, we found several unique characteristics with significant differences in various mental disorders: glycolysis, glycerophospholipid, and sphingolipid metabolism exhibited significant differences in SCZ; lysine, cysteine, and methionine metabolism in MDD and BD; and phenylalanine, tyrosine, and aspartate metabolism in SCZ and BD. Finally, we identified five panels of potential diagnostic biomarkers for MDD-HC, BD-HC, SCZ-HC, MDD-SCZ, and BD-SCZ comparisons.

The relationships among BD, MDD, and SCZ diagnoses have changed over the years. In the DSM-IV, MDD and BD were both listed in the same chapter (affective disorder) and compared with SCZ [29]. However, in the DSM-V, BD and related disorders were grouped in a separate chapter from depressive disorders and placed between the chapters of SCZ spectrum disorders and depressive disorders, indicating that BD is a bridge between the two diagnostic classes [30]. Our results showed that the most distinct differences in plasma metabolism were in SCZ, the metabolic differences in the BD-HC comparison had characteristics similar to those of both MDD and SCZ, and the BD metabolic profile was closer to that of MDD than that of SCZ, consistent with the diagnostic categorizations of both the DSM-IV and DSM-V. A previous lipid metabonomics study that included patients with SCZ, BD, MDD, and HCs found that all patients could be divided into two clusters (one mainly including SCZ and BD, and the other mainly including MDD and BD) [16]. Other metabolomics studies focusing on two of the three psychiatric disorders also suggested similar correlations that the metabolic differences between HCs and BD patients with shared characteristics of the metabolic profiles of both MDD and SCZ [31, 32].

Previous studies of plasma metabolism have reported that lower plasma concentrations of unsaturated fatty acids were associated with MDD [33], BD [34], and SCZ [35]. Furthermore, supplementation with unsaturated fatty acids has been shown to improve psychiatric symptoms [36, 37]. Our results showed that fatty acids and their intermediates in β-oxidative metabolism, acylcarnitine, were downregulated in almost all three disorders. Moreover, another important hormone in lipid metabolism, cortisone, has been widely reported to be a stress-related hormone. Both high and low cortisone levels have been reported to be associated with MDD and BD [38, 39]. Low cortisone levels have been reported to be associated with SCZ [40]. We found that cortisone levels were significantly reduced in all three disorders. In addition, perturbations of amino acid metabolism in psychiatric disorders have been reported in a series of two-group metabolomics studies focusing on SCZ [41, 42], MDD [43, 44], and BD [45, 46] compared with HCs. Our results also showed that glutamate, tryptophan, arginine, and proline metabolism were significantly different in all three disorders. These amino acids are involved in the synthesis and regulation of neurotransmitters and their receptors. For example, glutamate, arginine, and proline metabolism are involved in the synthesis and regulation of the excitatory neurotransmitter glutamate and its NMDA receptors, which is implicated in psychiatric disorders [42, 47–49]. Additionally, glutamate can be used to synthesize the essential inhibitory neurotransmitter gamma-aminobutyric acid (GABA), which counteracts the excitatory effects of glutamate [50, 51]. Moreover, tryptophan metabolism is involved in the production of not only the important neurotransmitter 5-HT but also its downstream metabolites, which have both neuroprotective (kynurenic acid) and neurotoxic (3-hydroxykynurenine and quinolinic acid) effects [52]. We also found that cAMP, a purine metabolite, was downregulated in the three psychiatric disorders; this metabolite is an important secondary messenger that regulates impulse conduction and gene expression of nerve cells through cAMP-PKA-calcium signaling [53–55], which is altered in psychiatric disorders [54, 56–59]. In conclusion, these consistent differences in plasma metabolism may imply shared pathologies of mental illness.

Interestingly, our results highlighted several unique metabolic characteristics among the different disorders. Glycerophospholipid and sphingolipid metabolism were significantly different in SCZ. This may be because SCZ induces more severe developmental and organic defects in nerve cells, which are often accompanied by more severe cell membrane damage [35]. We observed a significant perturbation in glycolysis metabolism in only SCZ. This perturbation may be attributed to impaired oxidative phosphorylation of mitochondria and increased glycolysis in SCZ patients, which requires the consumption of more sugars to meet the normal energy requirements [60]. Mitochondrial dysfunctions in SCZ patients play a more prominent role in the development and progression of the illness than those in BD and MDD [61]. Notably, antipsychotics can increase blood glucose concentrations while effectively controlling psychotic symptoms [62]. We also found that metabolism of the aromatic amino acids phenylalanine and tyrosine were significantly altered in SCZ and BD. Phenylalanine can be intracellularly converted to tyrosine, which is then converted to the important neurotransmitters epinephrine, norepinephrine, and dopamine [63]. Notably, downregulation of phenylalanine and tyrosine was observed in only BD and SCZ, which may imply that positive symptoms are associated with increased consumption of phenylalanine and tyrosine to synthesize catecholamines. In addition, we found that lysine, cysteine, and methionine metabolism were significantly altered in MDD and BD. Cysteine and methionine metabolism occur in the cysteine- cystathionine-γ-lyase (CSE)-H_2_S (hydrogen sulfide) pathway, generating H_2_S, which has antioxidant and neuroprotective effects [64, 65]. Lysine can regulate gene expression through acetylation or methylation, which has been shown to be associated with psychiatric disorders [66, 67]. Oxidative stress and epigenetic changes are the body’s responses to environmental stress [68, 69]. This implies that environmental factors may play a more important role in MDD and BD episodes than in SCZ.

It needs to be emphasized, our results of confounding factors (drug and dietary habits) should not be interpreted as evidence that these confounding factors have no effect on plasma metabolites. Instead, it suggests that in our studied population, the impact of these confounding factors are relatively mild compared to the impact of the disease. The current sample size is only sufficient to detect differences between diseases and not enough to distinguish the effects of these confounding factors on plasma metabolites. This intriguing research topic needs clarification through future studies with a larger sample size.

Our study had several limitations. First, although the total sample size was adequate, each group had a relatively small sample size. This made it impractical for us to verify the potential diagnostic biomarkers in the current cohort, and thus, these potential diagnostic biomarkers should be verified in future studies including validation cohorts. Second, our cross-sectional study focused on only adolescence, which is a critical period for the emergence of these major mental disorders and for early treatment, which can determine patient prognosis [3, 70]. However, there are etiological and treatment differences between adults and adolescents [71]; thus, future cross-sectional research on adults is needed to further advance this field. Longitudinal cohorts that follow individuals from adolescence to adulthood will provide a better understanding of these processes, but such studies require substantial resources. Third, to comprehensively explore these metabolic profiles, we utilized an untargeted metabolomics approach, which generated data in relative concentrations rather than in absolute concentrations (as generated by targeted metabolomics). This weakened the comparability of our results with other studies. Fourth, our sample size was not large enough to reveal the influence of confounding factors(drug and dietary habits).

In summary, our findings suggest significant differences in plasma metabolism between patients with major mental disorders and HCs; the most distinct changes were observed in SCZ patients. Moreover, the metabolic differences in BD (BD patients compared to HCs) seem to share characteristics of those of both MDD and SCZ, although the BD metabolic profile was closer to that of MDD than to that of SCZ. Additionally, we identified the metabolites responsible for the similar and unique metabolic characteristics in multiple metabolic pathways. The similar significant differences among the three disorders were observed in fatty acid, steroid-hormone, purine, nicotinate, glutamate, tryptophan, arginine, and proline metabolism. Additionally, unique characteristics of these disorders in terms of significant differences were found in glycolysis, glycerophospholipid, and sphingolipid metabolism for SCZ; lysine, cysteine, and methionine metabolism for MDD and BD; and phenylalanine, tyrosine, and aspartate metabolism for SCZ and BD. These critical metabolites provide clues of molecular mechanisms underlying these psychiatric disorders. Finally, five panels of potential diagnostic biomarkers were identified for MDD-HC, BD-HC, SCZ-HC, MDD-SCZ, and BD-SCZ comparisons.

### Supplementary information

Supplementary Materials

Supplementary Figure 1

Supplementary Figure 2

Supplementary Figure 3

Supplementary Figure 4

Supplementary Figure 5

Supplementary Table 1

Supplementary Table 2

Supplementary Table 3

Supplementary Table 4

Supplementary Table 5

Supplementary Table 6

Supplementary Table 7

Supplementary Table 8

## Data Availability

The data are available from the corresponding author on reasonable request.
