# Supplementary material for: Identifying plasma metabolic characteristics of major depressive disorder, bipolar disorder, and schizophrenia in adolescents
Source: Transl Psychiatry. 2024 Mar 26;14:163. doi: 10.1038/s41398-024-02886-z (PMC10966062; doi:10.1038/s41398-024-02886-z)
Supplement: Supplementary file 1 — Supplementary Materials [file 41398_2024_2886_MOESM1_ESM.docx]

**Supplementary Materials for**

***Identifying plasma metabolic characteristics of major depressive disorder, bipolar disorder, and schizophrenia in adolescents***

Bangmin Yin, Yuping Cai, Teng Teng, Xiaolin Wang, Xueer Liu, Xuemei Li, Jie Wang, Hongyan Wu, Yuqian He, Fandong Ren, Tianzhang Kou, Zheng-Jiang Zhu, Xinyu Zhou

This article contains：

Supplementary Methods

Supplementary Figures 1

Supplementary Figures 2

Supplementary Figures 3

Supplementary Figures 4

Supplementary Figures 5

Supplementary Tables 1

Supplementary Tables 2

Supplementary Tables 3

Supplementary Tables 4

Supplementary Tables 5

Supplementary Tables 6

Supplementary Tables 7

Supplementary Tables 8

**Supplementary Methods**

**Reagents and sample preparation for LC‒MS analysis**

LC‒MS grade water (H_2_O) and methanol (MeOH) were purchased from Honeywell (Muskegon, USA). Ammonium hydroxide (NH_4_OH) and ammonium acetate (NH_4_OAc) were purchased from Sigma‒Aldrich (St. Louis, USA). Metabolite chemical standards were purchased from J&K (Beijing, China), Sigma (St. Louis, USA), Carbosynth (Berkshire, UK), TCI (Tokyo, Japan) and Energy Chemical (Shanghai, China).

Human plasma samples (50 µL) were extracted using 150 μL of MeOH with internal standards (d3-leucine and d6-phenylalanine). The samples were then vortexed for 30 s and sonicated for 15 min. To precipitate proteins, the samples were incubated for 1 h at -20 °C, followed by 15 min of centrifugation at 13,500 rpm and 4 °C. The resulting supernatants were transferred to high-performance liquid chromatography (HPLC) vials and stored at -80 °C prior to LC‒MS/MS analysis.

**LC‒MS analysis**

The LC‒MS analysis protocol followed that in our previous publication^[1](#_ENREF_1)^. Data acquisition was performed using a Thermo Scientific Vanquish UHPLC system coupled to a Thermo Scientific Orbitrap Exploris 480. A Waters ACQUITY UPLC BEH Amide column (particle size, 1.7 μm; 100 mm (length) × 2.1 mm (i.d.)) and a Kinetex C18 column (2.6 μm, 2.1× 100 mm) were used for LC separation, and the column temperature was kept at 25 °C. For HILIC analysis, the mobile phase A was 25 mM ammonium hydroxide (NH_4_OH) + 25 mM ammonium acetate (NH_4_OAc) in water, and B was acetonitrile (ACN) for both the positive (ESI+) and negative (ESI-) modes. The flow rate was 0.5 mL/min, and the gradient was set as follows: 0−0.5 min, 95% B; 0.5−7 min, 95% B to 65% B; 7−8 min, 65% B to 40% B; 8−9 min, 40% B; 9−9.1 min, 40% B to 95% B; and 9.1−12 min, 95% B. The injection volume was 2 μL. For reversed-phase liquid chromatography (RPLC) analysis, mobile phase A was 0.01% acetic acid in water, and B was a mixture of indolepropionic acid (IPA) and ACN (1:1) for both the positive (ESI+) and negative (ESI-) modes. The flow rate was 0.3 mL/min, and the gradient was set as follows: 0−1 min: 1% B; 1−8 min: 99% B; 8−9 min: 99% B; 9.0−9.1 min, 99% B to 1% B; and 9.1−12 min: 1% B. The injection volume was 2 μL. All samples were randomly injected during data acquisition.

The data acquisition was operated in full MS-scan mode with a positive/negative ion polarity switch for individual samples. Information-dependent acquisition (IDA) mode was used for quality control (QC) samples to acquire MS/MS spectra. The source parameters were set as follows: spray voltage of 3,000 V or -3,000 V for positive or negative mode, respectively. The aux gas heater temperature was set at 350 °C. Sheath gas was set at 50 arb. Aux gas was set at 15 arb. The capillary temperature was set at 400 °C. The full MS resolution was set at 60,000, and the automatic gain control (AGC) target was 1e6 for positive or negative mode. The maximum IT was set at 100 ms. The mass range was set to 70-1,200 Da. For the dd-MS2 settings, the MS resolution was set at 30,000, and the AGC target was set at 1e5. The maximum IT was set at 60 ms. The Top N setting was set at 6. The isolation width was set at 1.0 m/z Da. The MS/MS spectra of the QC sample were acquired under a stepped normalized collision energy (SNCE) of 20-30-40%. The dynamic exclusion was set at 3.0 s, and the isotope exclusion was on.

**Metabolomics data processing**

The data processing protocol followed that in our previous publication^[2](#_ENREF_2), [3](#_ENREF_3)^. Briefly, ProteoWizard (version 3.0.20360) was first used to convert raw MS data (.raw) files to the mzXML format. Then, mzXML data files of samples were grouped for peak detection, retention time correction, and peak alignment using the R package “xcms” (version 3.2; https://bioconductor.org/packages/release/bioc/html/xcms.html). Key parameters were set as follows: method, “centWave”; ppm, 10; snthr, 3; peakwidth, c (5,30); and minfrac, 0.5. Then, missing value imputation and data normalization were conducted using MetFlow software (http://metflow.zhulab.cn/). Briefly, missing values were imputed by the 10 nearest neighbors using the k-nearest neighbor algorithm, and the resulting data were normalized by a supported vector algorithm based on QC samples to remove unwanted system error that occurred in the batch. Metabolic peaks with relative standard deviations (RSDs) less than 30% in QC samples were used for subsequent analysis. Metabolite annotation was performed using MetDNA (version 1.2.2; http://metdna.zhulab.cn/). The metabolite annotation parameters were set as “HILIC” or “RP” according to liquid chromatography mode and “30” or “SNCE_20_30_40%” for collision energy. We performed metabolite annotation separately in both positive and negative modes. According to the definition of the metabolomics standards initiative (MSI), we assigned the metabolite annotations with three confidence levels. Level 1 was used for metabolites annotated through matching of MS1, RT and MS/MS spectra with the in-house metabolite spectral library. Level 2 was used for metabolites annotated by matching MS1 and MS/MS2 spec with a public metabolite spectral library (mainly from NIST 2017). Level 3 was used for metabolites annotated based on MS1 and surrogate MS/MS match using MetDNA.

**Reference**

1. Wang H, Jia H. Serum metabolic traits reveal therapeutic toxicities and responses of neoadjuvant chemoradiotherapy in patients with rectal cancer. 2022; **13**(1): 7802.

2. Shen X, Wang R, Xiong X, et al. Metabolic reaction network-based recursive metabolite annotation for untargeted metabolomics. 2019; **10**(1): 1516.

3. Zhou Z, Luo M, Zhang H, Yin Y, Cai Y, Zhu ZJ. Metabolite annotation from knowns to unknowns through knowledge-guided multi-layer metabolic networking. 2022; **13**(1): 6656.

**Supplementary Figure**

**Supplementary Figure 1. The PCA plots of disorders and HC.**

1. The PCA plot showed a trend of differentiation, although with some overlap, between healthy control (HC) and three disorders. However, the three disorders mostly overlapped with each other.

(B) The PCA plot for the comparison between MDD and HC.

(C) The PCA plot for the comparison between BD and HC.

(D) The PCA plot for the comparison between SCZ and HC.

**Supplementary Figure 2. Impact of the confounding factor: drug treatment.**

1. The PCA plot showed no clear discrimination between MDD patients who received drug treatment and those who did not. (B) The PCA plot showed no clear discrimination between BD patients who received drug treatment and those who did not. (C) The PCA plot showed no clear discrimination between SCZ patients who received drug treatment and those who did not. (D) The PCA plot showed no clear discrimination between MDD patients who were fasting or not.

Abbreviation: DN, drug-naïve; DT, drug-treatment; F, fasting; NF, not-fasting.

**Supplementary Figure 3. Permutation tests for the disorder-HC comparisons PLS-DA and biplot PLS-DA.**

1. Permutation test for MDD-HC. (B) Permutation test for BD-HC. (C) Permutation test for SCZ-HC. (D) Permutation test for biplot PLS-DA.

**Supplementary Figure 4. Identifying differentially expressed metabolites and potential diagnostic metabolites for the disorder-disorder comparisons.**

1. The Venn diagram showed the union-set of differentially expressed metabolites from MDD-HC and SCZ-HC.

(B) The Venn diagram showed the union-set of differentially expressed metabolites from BD-HC and SCZ-HC.

(C) The Venn diagram showed the union-set of differentially expressed metabolites from MDD-HC and BD-HC.

(D) The Volcano plot for SCZ-MDD comparison showed the distribution of log_2_(FoldChange) and -log_10_(Pvalue) of metabolitesthat had been selected out by the Venn diagram. Differentially expressed metabolites for SCZ-MDD were filled in light blue.

(E) The Volcano plot for SCZ-BD showed the distribution of log_2_(FoldChange) and -log_10_(Pvalue) of metabolites that had been selected out by the Venn diagram. Differentially expressed metabolites for SCZ-BD were filled in green.

(F) The Volcano plot for MDD-BD showed the distribution of log_2_(FoldChange) and -log_10_(Pvalue) of metabolites that had been selected out by the Venn diagram.

(G) The ROC curve for SCZ-MDD potential diagnostic metabolites generated by PCR exhibited an AUC = 0.933.

(H) The ROC curve for SCZ-BD potential diagnostic metabolites generated by PCR exhibited an AUC = 0.867.

**Supplementary Figure 5. Potential diagnostic metabolites of the disorder-HC comparisons.**

(A) The ROC of potential diagnostic metabolites for MDD-HC generated by PCR showed an AUC = 0.962.

(B) The ROC of potential diagnostic metabolites for BD-HC generated by PCR showed an AUC = 0.983.

(C) The ROC of potential diagnostic metabolites for SCZ-HC generated by PCR showed an AUC = 0.994.

**Supplementary Table**

**Supplementary Table 1. Parameters of the PLS-DA models.**

The R2X, R2Y and Q2Y of the PLS-DA models of MDD-BD-SCZ-HC, MDD-HC, BD-HC, SCZ-HC and biplot comparisons.

**Supplementary Table 2. Detailed information of differentially expressed metabolites from the MDD-BD-SCZ-HC comparison.**

The Vip, FDR P.value and mean of MDD, BD, SCZ and HC groups for differentially expressed metabolites from the MDD-BD-SCZ-HC comparison.

**Supplementary Table 3. Detailed information of differentially expressed metabolites from the disorder-HC comparisons.**

The Vip, FC (fold change), FDR P.value and whether as biomarker for differentially expressed metabolites from the disorder-HC comparisons (MDD-HC, BD-HC and SCZ-HC).

**Supplementary Table 4. Detailed information of** **differentially expressed acetylcarnitine metabolites from the disorder-HC comparisons.**

The Vip, FC (fold change), FDR P.value and whether as biomarker for differentially expressed acetylcarnitine metabolites from the disorder-HC comparisons (MDD-HC, BD-HC and SCZ-HC).

**Supplementary Table 5. Detailed information of the disorder-disorder differentially expressed metabolites selected from respective union-set of differentially expressed metabolites from the disorder-HC comparisons.**

The FC (fold change), FDR P.value of respective overlaps of differentially expressed metabolites from the disorder-HC comparisons (MDD-SCZ from MDD-HC & SCZ-HC, BD-SCZ from BD-HC & SCZ-HC, MDD-BD from MDD-HC & BD-HC).

**Supplementary Table 6. The detailed parameters of LASSO and PCR models.**

The lambda.1se, intercept and coefficients of differentially expressed metabolites for LASSO models 1-10 , and the intercept and coefficients of biomarkers for PCR model of the five comparisons (MDD-HC, BD-HC, SCZ-HC, MDD-SCZ and BD-SCZ).

**Supplementary Table 7: Detailed information of medications in each disorder.**

**Supplementary Table 8. Dietary habit of patients with MDD, BD, SCZ and HCs.**

Continuous variables were presented as the mean±SD(standard deviation).The differences in starchy food, vegetable, meat, tea, coffee, and fruit were analyzed using the Kruskal-Wallis test followed by FDR.

Abbreviation: MDD, major depressive disorder; BD, [bipolar disorder](javascript:;); SCZ, [schizophrenia](javascript:;); HC, health control;
