# Supplementary figures and images for: Identifying plasma metabolic characteristics of major depressive disorder, bipolar disorder, and schizophrenia in adolescents
Source: Transl Psychiatry. 2024 Mar 26;14:163. doi: 10.1038/s41398-024-02886-z (PMC10966062; doi:10.1038/s41398-024-02886-z)

**A**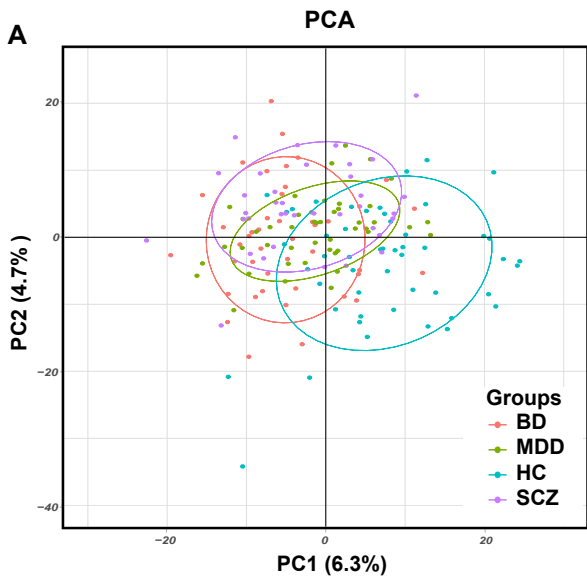**B**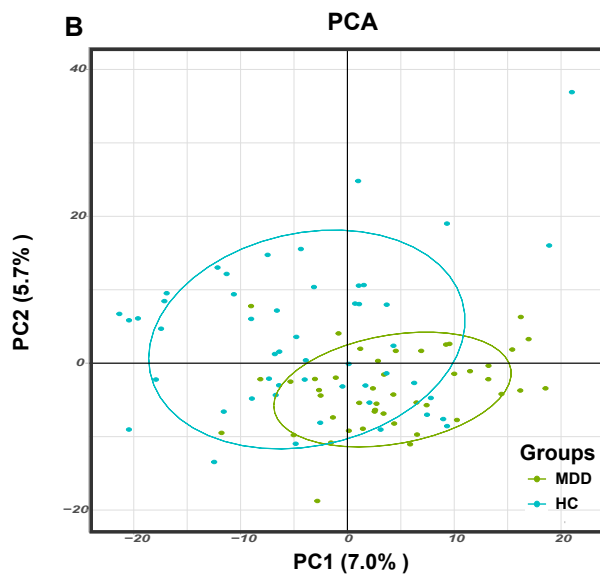**C**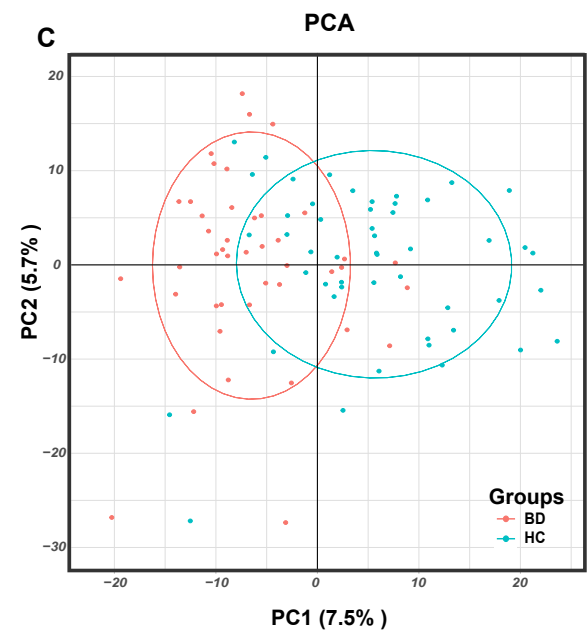**D**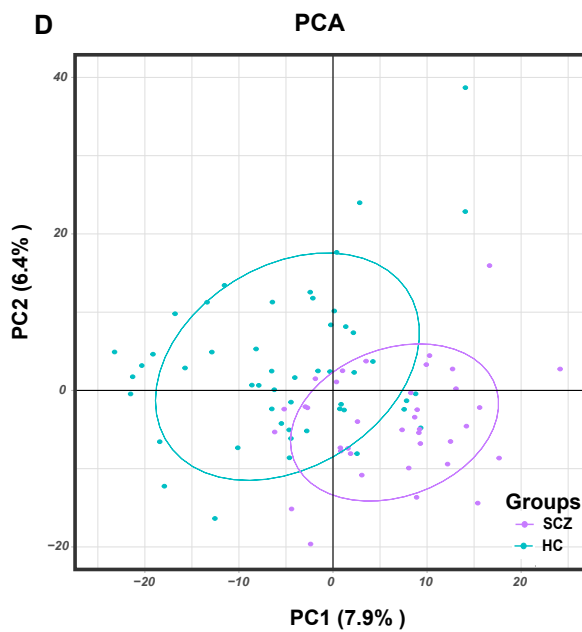

Supplement: Supplementary file 2 — Supplementary Figure 1 [file 41398_2024_2886_MOESM2_ESM.pdf]

PCA

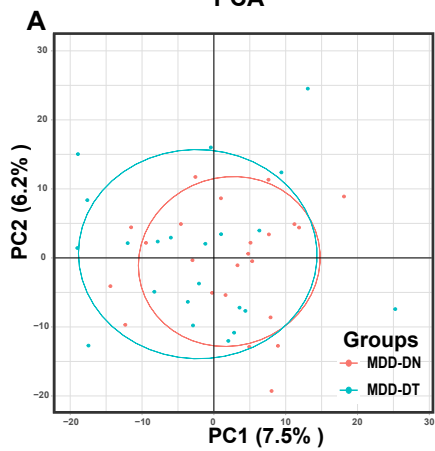

PCA

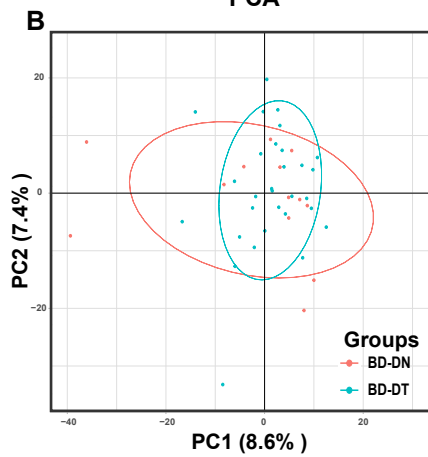

PCA

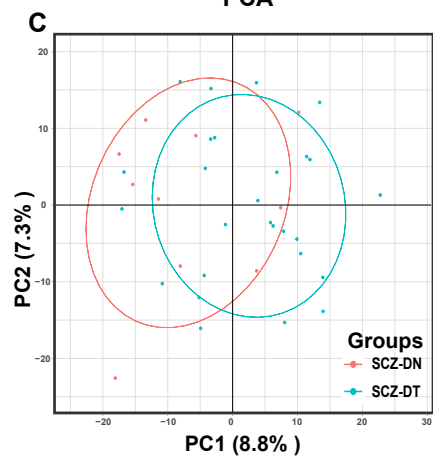

Supplement: Supplementary file 3 — Supplementary Figure 2 [file 41398_2024_2886_MOESM3_ESM.pdf]

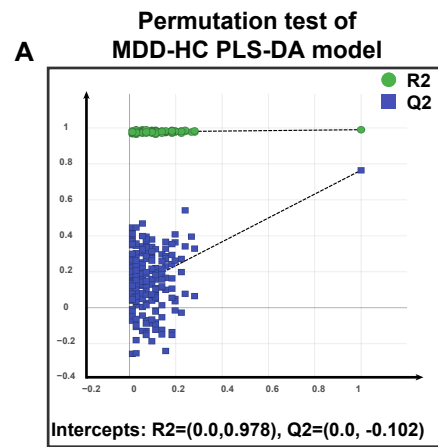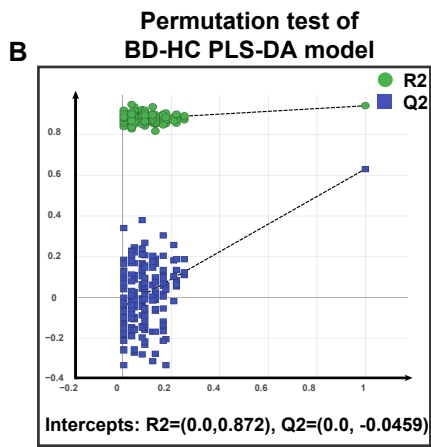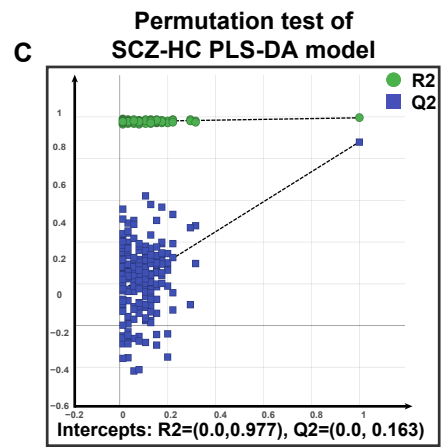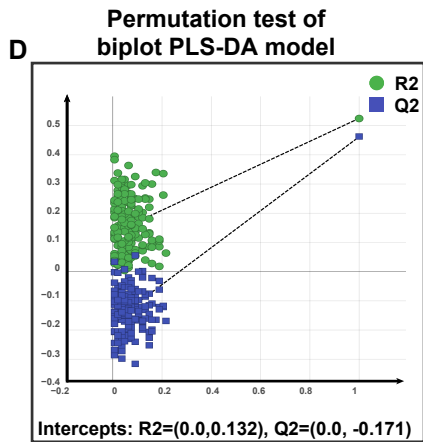

Supplement: Supplementary file 4 — Supplementary Figure 3 [file 41398_2024_2886_MOESM4_ESM.pdf]

**A**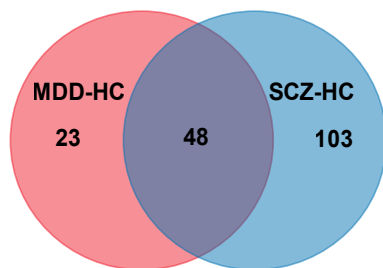**B**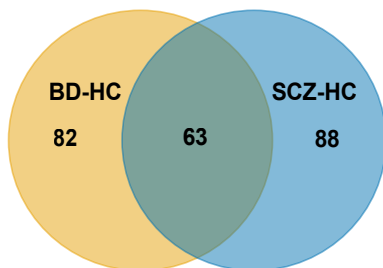**C**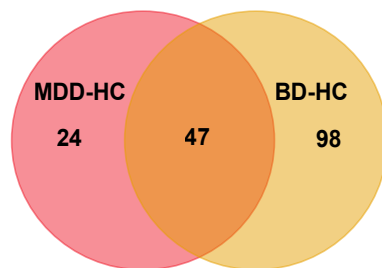**D**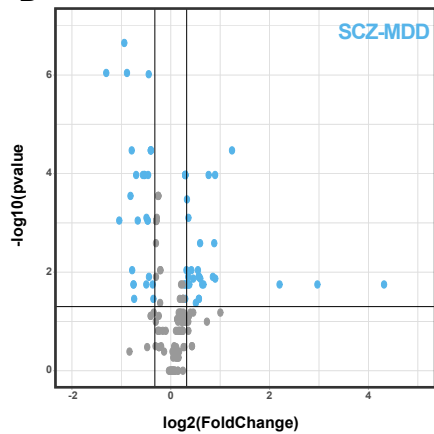**E**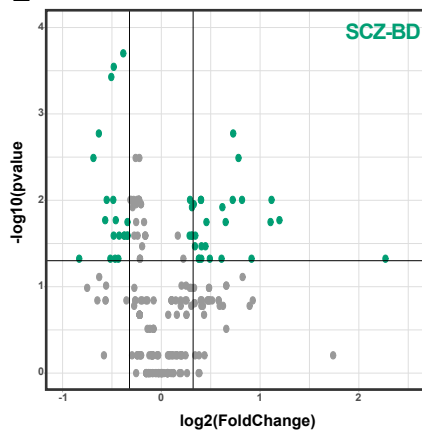**F**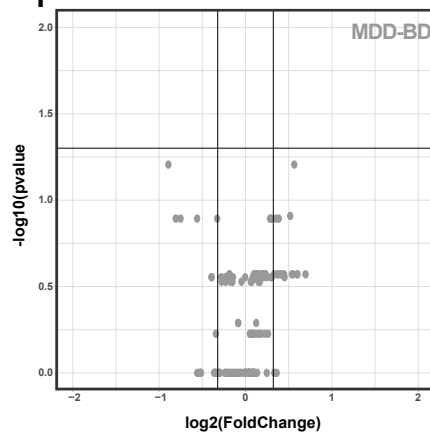**G**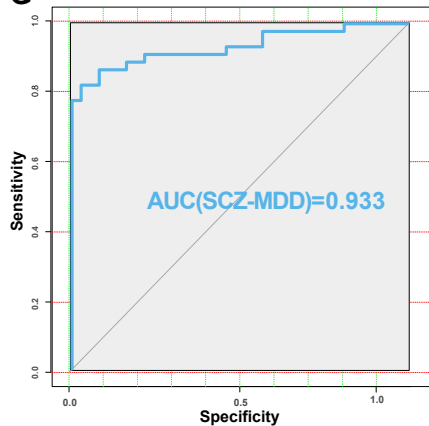**H**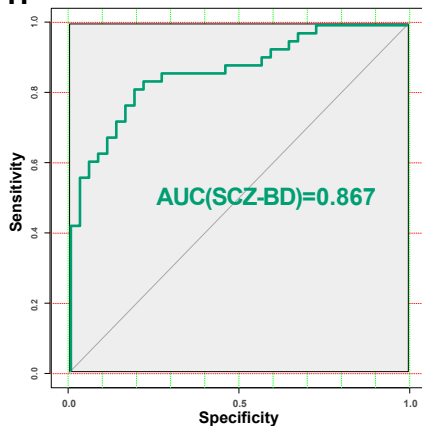

Supplement: Supplementary file 5 — Supplementary Figure 4 [file 41398_2024_2886_MOESM5_ESM.pdf]

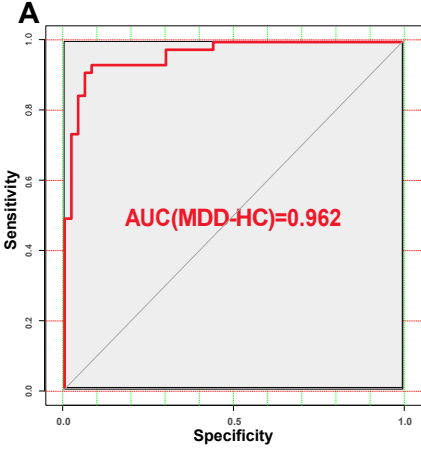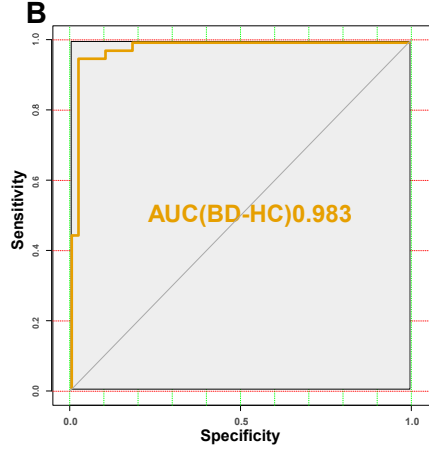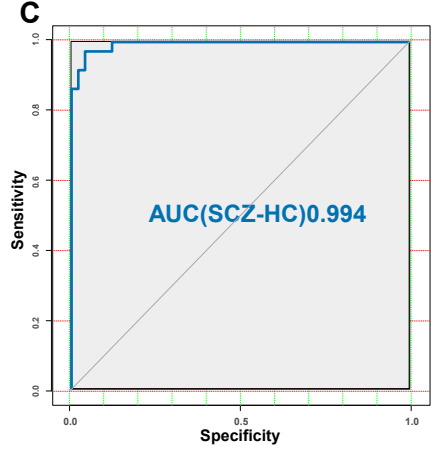

Supplement: Supplementary file 6 — Supplementary Figure 5 [file 41398_2024_2886_MOESM6_ESM.pdf]
