# Supplementary material for: Identifying plasma metabolic characteristics of major depressive disorder, bipolar disorder, and schizophrenia in adolescents
Source: Transl Psychiatry. 2024 Mar 26;14:163. doi: 10.1038/s41398-024-02886-z (PMC10966062; doi:10.1038/s41398-024-02886-z)
Supplement: Supplementary file 7 — Supplementary Table 1 [file 41398_2024_2886_MOESM7_ESM.pdf]

Supplementary Table 1. Parameters of the PLS-DA models.

Comparisons	R2X	R2Y	Q2Y
MDD-BD-SCZ-HC	0.179	0.641	0.28
MDD-HC	0.194	0.99	0.765
BD-HC	0.14	0.941	0.631
SCZ-HC	0.219	0.995	0.88
Biplot	0.34	0.355	0.248

The R2X, R2Y and Q2Y of the PLS-DA models of MDD-BD-SCZ-HC, MDD-HC, BD-HC, SCZ-HC and biplot comparisons.
