# Supplementary material for: Identifying plasma metabolic characteristics of major depressive disorder, bipolar disorder, and schizophrenia in adolescents
Source: Transl Psychiatry. 2024 Mar 26;14:163. doi: 10.1038/s41398-024-02886-z (PMC10966062; doi:10.1038/s41398-024-02886-z)
Supplement: Supplementary file 8 — Supplementary Table 2 [file 41398_2024_2886_MOESM8_ESM.pdf]

**Supplementary Table 2. Detailed information of differentially expressed metabolites from the MDD-BD-SCZ-HC comparison.**

| Name | Vip | FDR P.value | Mean-MDD | Mean-HC | Mean-SCZ | Mean-BD | Formula | Confidence_level |
|---|---|---|---|---|---|---|---|---|
| 2-Aminooctanoic acid | 1.34 | 4.17E-02 | 5964056.84 | 5889788.86 | 5758922.98 | 4164787.20 | C8H17NO2 | level1 |
| 2-Hydroxyoctanoic acid | 1.28 | 1.07E-02 | 13844436.16 | 12641830.07 | 10882101.99 | 9356431.98 | C8H16O3 | level1 |
| 2-Ketocaproic acid | 1.29 | 5.05E-03 | 1092941243.48 | 1309594553.79 | 1084154884.16 | 1025914582.41 | C6H10O3 | level1 |
| 2-Ketoisovaleric acid | 1.35 | 1.66E-03 | 1075311023.51 | 1216266537.55 | 1133249993.34 | 1029459304.43 | C5H8O3 | level1 |
| 3alpha-Hydroxy-12-oxo-5beta-cholan-24-oic acid | 1.07 | 4.17E-02 | 4805818.41 | 6924961.50 | 5444656.76 | 4599739.24 | C24H38O4 | level1 |
| 3alpha-Hydroxy-6-oxo-5alpha-cholan-24-oic acid | 1.07 | 4.17E-02 | 4805818.41 | 6924961.50 | 5444656.76 | 4599739.24 | C24H38O4 | level1 |
| 3-Amino-3-(4-hydroxyphenyl)propanoic acid | 1.02 | 6.96E-03 | 89899705.35 | 104144837.61 | 93890222.52 | 89449270.65 | C9H11NO3 | level1 |
| 3-Hydroxyoctanoic acid | 1.20 | 4.17E-02 | 15813556.69 | 19404214.03 | 15949013.91 | 42778456.64 | C8H16O3 | level1 |
| 3-Methyl-2-oxovaleric acid | 1.29 | 5.05E-03 | 1092941243.48 | 1309594553.79 | 1084154884.16 | 1025914582.41 | C6H10O3 | level1 |
| 3-methylcytidine | 1.83 | 5.03E-08 | 745571.83 | 947807.34 | 558580.47 | 585129.95 | C10H15N3O5 | level1 |
| 4-Vinylphenol | 1.47 | 1.93E-04 | 2270952.88 | 2972394.12 | 1434873.05 | 1893429.94 | C8H8O | level1 |
| 5-Aminopentanoic acid | 1.45 | 1.93E-04 | 12403257.58 | 14431690.93 | 12805646.36 | 11343761.37 | C5H11NO2 | level1 |
| 5-Hydroxytryptophan | 1.09 | 6.10E-03 | 374708.18 | 456423.13 | 370454.26 | 379430.12 | C11H12N2O3 | level1 |
| Acetoin | 1.66 | 6.10E-03 | 30351963.25 | 1642283.43 | 6597120.36 | 31807994.88 | C4H8O2 | level1 |
| Acetylcarnitine (Car(2:0)) | 1.43 | 6.10E-03 | 2308043931.75 | 2832792242.62 | 2081927218.56 | 2326852205.79 | C9H17NO4 | level1 |
| Adenosine 3',5'-cyclic phosphate (cAMP) | 1.45 | 5.75E-07 | 1639901.21 | 2119815.77 | 1444304.04 | 1515858.02 | C10H12N5O6P | level1 |
| Allose | 1.46 | 1.93E-04 | 32512005.69 | 33845243.31 | 30029726.22 | 29922379.24 | C6H12O6 | level1 |
| Apocholic acid | 1.07 | 4.17E-02 | 4805818.41 | 6924961.50 | 5444656.76 | 4599739.24 | C24H38O4 | level1 |
| Arachidonic acid (AA) | 1.78 | 5.75E-07 | 271340334.05 | 384410557.07 | 213083406.79 | 269777522.91 | C20H32O2 | level1 |
| Arginine | 1.83 | 3.07E-07 | 1036197481.83 | 1389037155.09 | 845922006.75 | 893153324.69 | C6H14N4O2 | level1 |
| Asparagine | 1.38 | 1.54E-02 | 35180833.49 | 37130982.31 | 39493664.08 | 33823373.83 | C4H8N2O3 | level1 |

| | | | | | | | | |
|---|---|---|---|---|---|---|---|---|
| Aspartic acid | 1.61 | 4.17E-02 | 23917905.63 | 21071466.42 | 14401758.59 | 16358869.69 | C4H7NO4 | level1 |
| Butyrylcarnitine | 1.04 | 4.17E-02 | 36134697.52 | 45716560.75 | 32658636.81 | 37696322.49 | C11H21NO4 | level1 |
| Capric acid | 1.75 | 6.96E-03 | 32691839.91 | 34756615.86 | 35009672.91 | 43223511.65 | C10H20O2 | level1 |
| Capric acid | 1.10 | 4.08E-04 | 126616763.00 | 177975505.93 | 111202999.25 | 155618350.54 | C10H20O2 | level1 |
| Caproic acid | 1.27 | 1.66E-03 | 30472999.27 | 34295731.16 | 31037478.21 | 32041652.55 | C6H12O2 | level1 |
| Caprylic acid | 1.19 | 1.07E-02 | 36863836.12 | 49362894.69 | 223156443.29 | 884360797.36 | C8H16O2 | level1 |
| Car(10:0)_RT370 | 1.36 | 6.52E-08 | 77057651.97 | 171719962.91 | 59371430.18 | 110323460.14 | C17H33NO4 | level1 |
| Car(10:1)_RT357 | 1.30 | 5.52E-05 | 67125649.95 | 113777987.84 | 54597061.94 | 73925991.64 | C17H31NO4 | level1 |
| Car(10:2)_RT333 | 1.06 | 4.17E-02 | 6835593.85 | 9586234.09 | 5810803.98 | 7335474.22 | C17H29NO4 | level1 |
| Car(10:2)_RT347 | 1.06 | 4.17E-02 | 6835593.85 | 9586234.09 | 5810803.98 | 7335474.22 | C17H29NO4 | level1 |
| Car(11:0)_RT390 | 1.55 | 4.09E-10 | 2460242.60 | 6812602.94 | 1320698.30 | 3025634.09 | C18H35NO4 | level1 |
| Car(11:1)_RT383 | 1.76 | 1.54E-08 | 13830422.59 | 28179220.73 | 11969850.79 | 13792110.49 | C18H33NO4 | level1 |
| Car(12:0)_RT430 | 1.58 | 6.52E-08 | 21332814.66 | 44682067.92 | 14568809.61 | 27440538.66 | C19H37NO4 | level1 |
| Car(12:1)_RT402 | 1.46 | 4.08E-04 | 20764850.21 | 37730497.64 | 16761698.76 | 22622913.89 | C19H35NO4 | level1 |
| Car(12:1-O)_RT364 | 1.35 | 6.46E-05 | 4361231.89 | 5379792.11 | 2415785.90 | 3186759.96 | C19H35NO5 | level1 |
| Car(12:2)_RT382 | 1.73 | 5.03E-08 | 5484377.90 | 9064449.09 | 3676275.51 | 4357279.11 | C19H33NO4 | level1 |
| Car(12:2-O)_RT340 | 1.24 | 6.96E-03 | 859554.54 | 894984.17 | 460615.63 | 583800.70 | C19H33NO5 | level1 |
| Car(14:0)_RT467 | 1.59 | 4.89E-04 | 6846840.02 | 10374904.98 | 6181426.41 | 8685203.36 | C21H41NO4 | level1 |
| Car(14:1)_RT438 | 1.42 | 2.65E-03 | 28737423.46 | 52234326.05 | 24443783.25 | 36552115.51 | C21H39NO4 | level1 |
| Car(14:1)_RT447 | 1.42 | 2.65E-03 | 28737423.46 | 52234326.05 | 24443783.25 | 36552115.51 | C21H39NO4 | level1 |
| Car(14:1-O)_RT416 | 1.73 | 1.67E-06 | 3724557.82 | 7582996.17 | 2716829.47 | 3610660.42 | C21H39NO5 | level1 |
| Car(14:2-O)_RT389 | 1.70 | 5.75E-07 | 1360254.64 | 2562407.88 | 899326.93 | 1284599.90 | C21H37NO5 | level1 |
| Car(16:1-O)_RT453 | 1.57 | 1.66E-03 | 1269455.85 | 2078298.19 | 1071073.86 | 1418291.40 | C23H43NO5 | level1 |
| Car(16:2)_RT456 | 1.16 | 4.17E-02 | 3935524.17 | 5517518.28 | 3140291.79 | 4953149.25 | C23H41NO4 | level1 |
| Car(16:2-O)_RT395 | 1.67 | 6.46E-05 | 2540520.42 | 3795010.65 | 1787274.67 | 2140497.19 | C23H41NO5 | level1 |
| Car(16:3)_RT442 | 1.61 | 1.93E-04 | 3109462.20 | 5243427.03 | 2786972.59 | 3630424.12 | C23H39NO4 | level1 |
| Car(16:4)_RT421 | 1.54 | 1.93E-04 | 1275352.62 | 2587761.83 | 1182372.72 | 1822873.87 | C23H37NO4 | level1 |

| | | | | | | | | |
|---|---|---|---|---|---|---|---|---|
| Car(18:1) | 1.41 | 6.96E-03 | 46674579.21 | 51615217.83 | 68941673.77 | 56341737.61 | C25H47NO4 | level1 |
| Car(3:0)_RT63 | 1.23 | 1.66E-03 | 18162708.56 | 23569695.94 | 17638724.02 | 17576590.08 | C10H19NO4 | level1 |
| Car(4:0)_RT100 | 1.04 | 4.17E-02 | 36134697.52 | 45716560.75 | 32658636.81 | 37696322.49 | C11H21NO4 | level1 |
| Car(6:0)_RT250 | 1.24 | 1.93E-04 | 11666872.39 | 17687969.56 | 10661090.97 | 16819822.95 | C13H25NO4 | level1 |
| Car(6:0)_RT263 | 1.24 | 1.93E-04 | 11666872.39 | 17687969.56 | 10661090.97 | 16819822.95 | C13H25NO4 | level1 |
| Car(8:0)_RT316 | 1.27 | 5.75E-07 | 43294247.33 | 88088858.18 | 33594406.46 | 63811095.82 | C15H29NO4 | level1 |
| Car(9:0)_RT346 | 1.83 | 2.16E-09 | 2777377.15 | 5992444.67 | 1509358.04 | 3270825.59 | C16H31NO4 | level1 |
| Chenodeoxycholic acid | 1.11 | 1.07E-02 | 30186884.17 | 55118063.43 | 50885549.92 | 39637701.15 | C24H40O4 | level1 |
| cis-8,11,14-Eicosatrienoic acid | 1.58 | 6.96E-03 | 72271488.44 | 109296577.57 | 70120542.30 | 83012568.12 | C20H34O2 | level1 |
| cis-Aconitic acid | 1.24 | 2.42E-02 | 56837257.25 | 64129942.25 | 54718169.08 | 51305625.28 | C6H6O6 | level1 |
| Cortisol | 1.49 | 2.63E-02 | 26628784.42 | 31141884.98 | 37420737.99 | 26044845.17 | C21H30O5 | level1 |
| Cortisone/Aldosterone | 1.59 | 1.39E-05 | 6316803.96 | 8673028.85 | 6287183.44 | 5884126.87 | C21H28O5 | level1 |
| Cortisone/Aldosterone | 1.59 | 1.39E-05 | 6316803.96 | 8673028.85 | 6287183.44 | 5884126.87 | C21H28O5 | level1 |
| Deoxycholic acid | 1.11 | 1.07E-02 | 30186884.17 | 55118063.43 | 50885549.92 | 39637701.15 | C24H40O4 | level1 |
| Dihydroxyacetone | 1.36 | 1.39E-05 | 3190855748.53 | 2741849238.80 | 4613504504.71 | 4000194137.42 | C3H6O3 | level1 |
| Dimethylglycine | 1.16 | 2.63E-02 | 70874352.61 | 64157953.58 | 90435237.14 | 74815030.46 | C4H9NO2 | level1 |
| Docosahexaenoic acid (DHA) | 1.73 | 6.46E-05 | 193211881.86 | 291120552.63 | 164607038.94 | 217834986.69 | C22H32O2 | level1 |
| Dodecanoic acid | 1.51 | 1.66E-03 | 228801161.14 | 311672304.26 | 147092596.56 | 259143972.33 | C12H24O2 | level1 |
| Eicosapentaenoic acid | 1.50 | 1.93E-04 | 31036783.54 | 48096104.58 | 25953723.87 | 36482647.43 | C20H30O2 | level1 |
| Epinephrine | 1.11 | 1.07E-02 | 19730156.52 | 6260314.50 | 23525608.68 | 15905822.03 | C9H13NO3 | level1 |
| Erucamide | 1.21 | 2.63E-02 | 3880088.65 | 2194924.31 | 2073555.41 | 4462602.74 | C22H43NO | level1 |
| Formylmethionine | 1.32 | 4.08E-04 | 8531882.03 | 9714933.16 | 8383020.52 | 7801002.66 | C6H11NO3S | level1 |
| Galactose | 1.46 | 1.93E-04 | 32512005.69 | 33845243.31 | 30029726.22 | 29922379.24 | C6H12O6 | level1 |
| gamma-Glutamyllysine | 1.77 | 4.08E-04 | 950124.72 | 1488683.05 | 1339299.09 | 903188.01 | C11H21N3O5 | level1 |
| gamma-Glutamylvaline | 1.22 | 1.54E-02 | 451794.94 | 523770.80 | 485211.48 | 406640.75 | C10H18N2O5 | level1 |
| Glucose | 1.46 | 1.93E-04 | 32512005.69 | 33845243.31 | 30029726.22 | 29922379.24 | C6H12O6 | level1 |
| Glucuronic acid | 1.34 | 4.17E-02 | 11606640.11 | 9908032.30 | 10812463.39 | 12905607.41 | C6H10O7 | level1 |

| | | | | | | | | | |
|---|---|---|---|---|---|---|---|---|---|
| Glutamic acid | 1.34 | 4.17E-02 | 40214376.99 | 31117552.83 | 45011808.91 | 41573473.92 | C5H9NO4 | level1 |
| Glutamine | 1.50 | 4.08E-04 | 715359617.48 | 754861732.79 | 760450022.46 | 703470514.09 | C5H10N2O3 | level1 |
| Glyceraldehyde | 1.36 | 1.39E-05 | 3190855748.53 | 2741849238.80 | 4613504504.71 | 4000194137.42 | C3H6O3 | level1 |
| Glycerophosphocholine | 1.35 | 6.10E-03 | 521835578.19 | 498922828.67 | 634111532.07 | 538194301.32 | C8H20NO6P | level1 |
| Heptadecanoic acid | 1.56 | 6.96E-03 | 63892075.51 | 94209640.54 | 59477651.41 | 81070728.68 | C17H34O2 | level1 |
| Histidine | 1.43 | 2.63E-02 | 520783271.91 | 558747309.85 | 577539801.55 | 564437906.59 | C6H9N3O2 | level1 |
| Histidinol | 1.67 | 1.66E-03 | 254367345.91 | 323228461.25 | 300913873.06 | 236961110.76 | C6H11N3O | level1 |
| Homoarginine | 1.08 | 6.96E-03 | 79624778.39 | 97050948.19 | 88758510.76 | 77590232.59 | C7H16N4O2 | level1 |
| Indole | 1.17 | 6.10E-03 | 3859224.13 | 4328383.47 | 3738931.41 | 3603959.34 | C8H7N | level1 |
| Indole-3-pyruvic acid | 1.10 | 4.17E-02 | 5197455.27 | 6585242.18 | 4500361.05 | 4873590.51 | C11H9NO3 | level1 |
| Indolelactic acid | 1.01 | 4.50E-03 | 33523719.12 | 42322633.10 | 30261388.62 | 31641519.79 | C11H11NO3 | level1 |
| Isocaproic acid | 1.27 | 1.66E-03 | 30472999.27 | 34295731.16 | 31037478.21 | 32041652.55 | C6H12O2 | level1 |
| Isoleucine | 1.44 | 5.05E-03 | 249871310.24 | 292788199.76 | 302556039.71 | 253806201.74 | C6H13NO2 | level1 |
| Isovalerylcarnitine | 1.03 | 2.63E-02 | 64956813.25 | 78428011.39 | 56931879.86 | 60704104.40 | C12H23NO4 | level1 |
| Ketoleucine | 1.29 | 5.05E-03 | 1092941243.48 | 1309594553.79 | 1084154884.16 | 1025914582.41 | C6H10O3 | level1 |
| Kynurenic acid | 1.25 | 1.93E-04 | 7055655.56 | 9279731.50 | 5894432.70 | 6317401.87 | C10H7NO3 | level1 |
| Kynurenine | 1.41 | 4.08E-04 | 8924739.59 | 11560871.10 | 8991990.75 | 8785317.13 | C10H12N2O3 | level1 |
| Lactic acid | 1.45 | 1.67E-06 | 548116656.53 | 425536309.03 | 770632068.06 | 645266805.49 | C3H6O3 | level1 |
| Leucine | 1.44 | 5.05E-03 | 249871310.24 | 292788199.76 | 302556039.71 | 253806201.74 | C6H13NO2 | level1 |
| LPC(15:0) | 1.38 | 4.17E-02 | 20871623.46 | 25505559.33 | 21754338.67 | 28374642.50 | C23H48NO7P | level1 |
| Lysine | 1.69 | 1.93E-04 | 112458713.50 | 159302536.22 | 152698113.84 | 108962998.53 | C6H14N2O2 | level1 |
| Malic acid | 1.16 | 6.96E-03 | 32397871.46 | 37267436.32 | 37022699.75 | 29194486.91 | C4H6O5 | level1 |
| Mannose | 1.46 | 1.93E-04 | 32512005.69 | 33845243.31 | 30029726.22 | 29922379.24 | C6H12O6 | level1 |
| Methionine | 1.61 | 1.39E-05 | 55064055.23 | 70241160.68 | 63653699.23 | 53691477.94 | C5H11NO2S | level1 |
| N,N-Dimethylarginine (ADMA) | 1.57 | 6.96E-03 | 180734054.24 | 193416132.61 | 202385833.78 | 173780762.61 | C8H18N4O2 | level1 |
| N1-Methyl-4-pyridone-3-carboxamide | 1.08 | 6.10E-03 | 12456646.25 | 17632728.03 | 12322577.66 | 11828188.03 | C7H8N2O2 | level1 |
| N2,N2-Dimethylguanosine | 1.07 | 1.20E-02 | 1507191.43 | 1759425.17 | 1590630.71 | 1520707.98 | C12H17N5O5 | level1 |

| | | | | | | | | |
|---|---|---|---|---|---|---|---|---|
| N-Acetylalanine | 1.06 | 6.96E-03 | 5209226.76 | 5327900.66 | 5809858.62 | 4873903.81 | C5H9NO3 | level1 |
| Nicotinamide | 1.18 | 4.08E-04 | 7766529.64 | 10910058.74 | 13979875.41 | 11056575.48 | C6H6N2O | level1 |
| Nordeoxycholic acid | 1.51 | 4.08E-04 | 4424045.41 | 6753245.05 | 4318573.24 | 3515973.52 | C23H38O4 | level1 |
| Norleucine | 1.17 | 2.63E-02 | 161332422.55 | 184108850.53 | 185748473.33 | 160350401.88 | C6H13NO2 | level1 |
| Normetanephrine | 1.11 | 1.07E-02 | 19730156.52 | 6260314.50 | 23525608.68 | 15905822.03 | C9H13NO3 | level1 |
| Oleic acid | 1.32 | 1.24E-02 | 1200092560.62 | 1361837769.36 | 1146611642.99 | 1306002077.85 | C18H34O2 | level1 |
| Ornithine | 1.83 | 6.10E-03 | 31753767.72 | 33579847.27 | 50464705.34 | 31329413.78 | C5H12N2O2 | level1 |
| PC(36:3)_RT642 | 1.16 | 2.42E-02 | 263246858.88 | 268747212.02 | 211173406.32 | 279136301.09 | C44H82NO8P | level1 |
| PC(37:6)_RT608 | 1.32 | 4.17E-02 | 1944907.47 | 2541404.87 | 1650175.26 | 2532791.90 | C45H78NO8P | level1 |
| PC(40:7)_RT616 | 1.17 | 4.17E-02 | 15649230.49 | 17579406.85 | 13963459.25 | 17313045.05 | C48H82NO8P | level1 |
| Pentadecanoic acid | 1.43 | 4.17E-02 | 62726369.78 | 76760112.11 | 60534749.53 | 72017188.28 | C15H30O2 | level1 |
| Phenylalanine | 1.33 | 2.63E-02 | 412917406.89 | 455344775.40 | 431579212.97 | 388394907.88 | C9H11NO2 | level1 |
| Phenylpyruvic acid | 1.44 | 1.39E-05 | 3607862.38 | 4215421.79 | 2816314.77 | 3115783.93 | C9H8O3 | level1 |
| Pristanic acid | 1.46 | 6.96E-03 | 7228412.74 | 9820272.35 | 6212216.82 | 8525264.45 | C19H38O2 | level1 |
| Pyroglutamic acid | 1.62 | 5.75E-07 | 544537643.92 | 500418093.62 | 741956258.20 | 635754207.16 | C5H7NO3 | level1 |
| Pyruvic acid | 1.14 | 1.93E-03 | 2711454365.39 | 1892914781.49 | 3356363751.61 | 3179024939.61 | C3H4O3 | level1 |
| S1P(d18:0) | 1.02 | 6.10E-03 | 4471180.01 | 3855424.14 | 5190917.36 | 4394815.45 | C18H40NO5P | level1 |
| S1P(d18:1) | 1.28 | 1.93E-04 | 23046151.34 | 20833672.66 | 28364598.67 | 23341904.19 | C18H38NO5P | level1 |
| S1P(d18:2)_RT463 | 1.32 | 1.66E-03 | 21375247.84 | 18593195.95 | 26290244.34 | 21271268.92 | C18H36NO5P | level1 |
| S-Adenosylhomocysteine | 1.35 | 4.17E-02 | 594156.76 | 696374.27 | 771556.02 | 616230.55 | C14H20N6O5S | level1 |
| Serine | 1.22 | 6.96E-03 | 82972053.07 | 78014174.25 | 89268426.85 | 83608466.27 | C3H7NO3 | level1 |
| Sorbose | 1.46 | 1.93E-04 | 32512005.69 | 33845243.31 | 30029726.22 | 29922379.24 | C6H12O6 | level1 |
| Stearamide | 1.10 | 1.93E-04 | 854617.39 | 974704.61 | 42893.08 | 427967.37 | C18H37NO | level1 |
| Taurine | 1.51 | 6.96E-03 | 892539439.55 | 998049856.44 | 869394157.99 | 855674977.96 | C2H7NO3S | level1 |
| trans-Vaccenic acid | 1.32 | 1.24E-02 | 1200092560.62 | 1361837769.36 | 1146611642.99 | 1306002077.85 | C18H34O2 | level1 |
| Traumatic acid | 1.09 | 2.42E-02 | 1663261.35 | 1704840.89 | 1125759.28 | 1254148.71 | C12H20O4 | level1 |
| Tryptophan | 1.58 | 6.46E-05 | 995555223.15 | 1242332833.93 | 1057784942.04 | 1000050566.93 | C11H12N2O2 | level1 |

| | | | | | | | | |
|---|---|---|---|---|---|---|---|---|
| Tyrosine | 1.42 | 4.08E-04 | 325973279.39 | 390753477.86 | 310387510.80 | 303405050.24 | C9H11NO3 | level1 |
| Tyrosine O-sulfate | 1.31 | 1.93E-04 | 9572166.37 | 11939652.62 | 9436927.61 | 9106149.66 | C9H11NO6S | level1 |
| Undecanoic acid | 1.48 | 4.08E-04 | 10170081.09 | 12610279.90 | 9798568.47 | 11984303.04 | C11H22O2 | level1 |
| Uric acid | 1.20 | 4.17E-02 | 282954491.18 | 318991947.47 | 297857429.52 | 271955270.12 | C5H4N4O3 | level1 |
| Uridine | 1.68 | 4.50E-03 | 202088874.78 | 235185615.52 | 253365348.04 | 211532129.39 | C9H12N2O6 | level1 |
| Valine | 1.24 | 2.63E-02 | 238503814.30 | 263175036.85 | 260663717.58 | 230396947.86 | C5H11NO2 | level1 |
| Xanthine | 1.16 | 4.08E-04 | 22244445.04 | 29012943.57 | 30564002.53 | 26935183.20 | C5H4N4O2 | level1 |
| Xanthurenic acid | 1.60 | 5.75E-07 | 584878.70 | 797143.38 | 343883.07 | 567523.49 | C10H7NO4 | level1 |
| 2-Hydroxyhexanedioic acid | 1.50 | 9.88E-06 | 16012918.60 | 16422371.93 | 12758151.10 | 14321181.71 | C6H10O5 | level2 |
| 4-Hydroxyglutamic acid | 1.48 | 6.96E-03 | 47532289.88 | 35361087.03 | 37786327.81 | 47958802.13 | C5H9NO5 | level2 |
| Arabinono-1,4-lactone | 1.60 | 4.08E-04 | 1703224.20 | 1879766.68 | 1128553.45 | 1939537.13 | C5H8O5 | level2 |
| Camphor | 1.26 | 4.17E-02 | 32582204.92 | 25653255.94 | 38198251.04 | 28416541.88 | C10H16O | level2 |
| Car(11:1-O2)_RT289 | 2.61 | 1.66E-03 | 13107745.46 | 22801958.95 | 22525568.39 | 24339085.11 | C18H31NO6 | level2 |
| Car(12:1-O2)_RT315 | 1.90 | 2.63E-02 | 14227211.66 | 24700069.34 | 23773797.28 | 24904511.26 | C19H33NO6 | level2 |
| Car(13:0)_RT433 | 1.24 | 1.93E-04 | 6937000.84 | 11135358.88 | 5369264.34 | 8431941.74 | C20H39NO4 | level2 |
| Car(14:2)_RT416 | 1.42 | 1.93E-04 | 49544818.68 | 81567271.94 | 37602451.94 | 56629584.11 | C21H37NO4 | level2 |
| Car(15:1-O)_RT438 | 1.70 | 6.46E-05 | 23862544.71 | 42518277.26 | 24158133.43 | 21732687.70 | C22H41NO5 | level2 |
| Car(16:1-O2)_RT414 | 1.64 | 9.88E-06 | 1021679.89 | 1593770.92 | 842625.95 | 914524.45 | C23H41NO6 | level2 |
| Car(18:1)_RT511 | 1.32 | 4.17E-02 | 50944648.01 | 59303351.87 | 72336556.48 | 60024351.30 | C25H47NO4 | level2 |
| Car(18:2)_RT502 | 1.22 | 6.10E-03 | 83568653.81 | 76035573.90 | 115543069.19 | 92958148.50 | C25H45NO4 | level2 |
| Car(5:1)_RT214 | 1.29 | 6.96E-03 | 4167798.04 | 5419545.61 | 3721592.11 | 3787160.47 | C12H21NO4 | level2 |
| Car(7:0)_RT288 | 1.31 | 1.67E-06 | 2472768.76 | 3703180.09 | 2073269.86 | 3163394.02 | C14H27NO4 | level2 |
| Car(9:1)_RT320 | 1.08 | 4.50E-03 | 10827239.36 | 13745287.37 | 9956157.75 | 9935103.69 | C16H29NO4 | level2 |
| Cer(d18:1/16:0) | 1.14 | 2.42E-02 | 17372204.58 | 17388810.87 | 13598828.72 | 17948566.69 | C34H67NO3 | level2 |
| Cysteine | 1.36 | 1.66E-03 | 2060991.73 | 2916586.46 | 2541990.77 | 1989064.82 | C3H7NO2S | level2 |
| Lactaldehyde | 1.53 | 1.67E-06 | 9028287.39 | 11204485.18 | 8075171.68 | 8722515.25 | C3H6O2 | level2 |
| Lithocholic acid 3-sulfate | 1.08 | 4.17E-02 | 644993.71 | 762122.36 | 923349.54 | 649787.70 | C24H40O6S | level2 |

| | | | | | | | | |
|---|---|---|---|---|---|---|---|---|
| PC(18:0/14:0) | 1.21 | 4.17E-02 | 69892536.44 | 59740546.34 | 40613419.73 | 56481218.12 | C40H80NO8P | level2 |
| PC(35:5)_RT602 | 1.28 | 4.17E-02 | 76913185.14 | 71080390.09 | 58752561.19 | 69160881.70 | C43H76NO8P | level2 |
| PC(39:6)_RT628 | 1.43 | 1.66E-03 | 12587005.59 | 16589099.21 | 10423128.59 | 17259705.13 | C47H82NO8P | level2 |
| PC(40:4)_RT600 | 1.14 | 2.42E-02 | 131530813.64 | 132530182.44 | 109852072.31 | 130366405.65 | C48H88NO8P | level2 |
| PC(42:9)_RT602 | 1.13 | 4.17E-02 | 41964045.95 | 43903394.21 | 37142684.62 | 43347705.27 | C50H82NO8P | level2 |
| (3Z)-Phytochromobilin | 1.26 | 1.66E-03 | 3225378.06 | 6362703.30 | 4274053.37 | 4010517.22 | C33H36N4O6 | level3 |
| (R)-10-Hydroxystearate | 1.70 | 1.93E-04 | 1938460.85 | 2711388.51 | 1840450.16 | 1943591.54 | C18H36O3 | level3 |
| 15,16-Dihydrobiliverdin | 1.26 | 1.66E-03 | 3225378.06 | 6362703.30 | 4274053.37 | 4010517.22 | C33H36N4O6 | level3 |
| 16-Oxopalmitate | 1.06 | 1.07E-02 | 822048.45 | 1016536.77 | 686320.21 | 633333.76 | C16H30O3 | level3 |
| 16-Oxopalmitate | 1.57 | 6.96E-03 | 6437759.42 | 9418559.45 | 5728327.34 | 6564592.20 | C16H30O3 | level3 |
| 16-Oxopalmitate | 1.28 | 1.93E-04 | 12471614.60 | 7520883.17 | 11751257.58 | 13491794.89 | C16H30O3 | level3 |
| 18-Hydroxyoleate | 1.81 | 1.93E-04 | 11559703.23 | 17988377.94 | 9716772.01 | 12042192.57 | C18H34O3 | level3 |
| 18-Hydroxyoleate | 1.20 | 1.20E-02 | 23669245.24 | 16578776.07 | 27910288.79 | 25104759.23 | C18H34O3 | level3 |
| 18-Oxooleate | 1.62 | 4.08E-04 | 6798139.89 | 9868451.09 | 5739985.48 | 7223715.04 | C18H32O3 | level3 |
| 1-Aminocyclopropane-1-carboxylate | 1.19 | 2.42E-02 | 88616224.21 | 96016293.23 | 99293351.85 | 78537723.79 | C4H7NO2 | level3 |
| 2,5-Dioxopentanoate | 1.41 | 1.93E-04 | 25571415.30 | 28613577.78 | 24010006.98 | 23428586.86 | C5H6O4 | level3 |
| 2,6-Dihydroxynicotinate | 1.86 | 5.75E-07 | 4020986.36 | 4867390.27 | 4498129.45 | 3755745.86 | C6H5NO4 | level3 |
| 2-Hydroxy-3-carboxybenzalpyruvate | 1.32 | 6.96E-03 | 22998609.48 | 22628396.47 | 20104212.68 | 20938963.32 | C11H8O6 | level3 |
| 2-Hydroxyhepta-2,4-dienedioate | 1.42 | 1.93E-04 | 8485424.57 | 8050560.58 | 10106718.88 | 9029688.52 | C7H8O5 | level3 |
| 2-Iminobutanoate | 1.19 | 2.42E-02 | 88616224.21 | 96016293.23 | 99293351.85 | 78537723.79 | C4H7NO2 | level3 |
| 2-Oxo-4-hydroxy-5-aminovalerate | 1.41 | 1.93E-04 | 25571415.30 | 28613577.78 | 24010006.98 | 23428586.86 | C5H9NO4 | level3 |
| 2-Oxohept-3-enedioate | 1.42 | 1.93E-04 | 8485424.57 | 8050560.58 | 10106718.88 | 9029688.52 | C7H8O5 | level3 |
| 3-(4-Hydroxyphenyl)pyruvate | 1.34 | 4.08E-04 | 18039127.34 | 27144639.48 | 12153406.22 | 16831036.41 | C9H8O4 | level3 |
| 3-(Uracil-1-yl)-L-alanine | 1.17 | 1.07E-02 | 5228114.78 | 4762662.99 | 5804633.24 | 5259939.10 | C7H9N3O4 | level3 |
| 3alpha,7alpha-Dihydroxy-5beta-cholestanate | 1.40 | 1.67E-06 | 1742404.72 | 1611622.28 | 3013020.24 | 2120497.48 | C27H46O4 | level3 |
| 3-Aminopentanedioate | 1.41 | 1.93E-04 | 25571415.30 | 28613577.78 | 24010006.98 | 23428586.86 | C5H9NO4 | level3 |

| | | | | | | | | |
|---|---|---|---|---|---|---|---|---|
| 3beta,7alpha-Dihydroxy-5-cholestenoate | 1.26 | 3.07E-07 | 1914174.08 | 1833443.17 | 3537608.98 | 2533056.14 | C27H44O4 | level3 |
| 3-Chloro-D-alanine | 1.13 | 4.17E-02 | 81897938.54 | 101692382.67 | 70040598.05 | 80204599.27 | C3H6ClNO2 | level3 |
| 3-Chloro-L-alanine | 1.13 | 4.17E-02 | 81897938.54 | 101692382.67 | 70040598.05 | 80204599.27 | C3H6ClNO2 | level3 |
| 3-Dehydroshikimate | 1.42 | 1.93E-04 | 8485424.57 | 8050560.58 | 10106718.88 | 9029688.52 | C7H8O5 | level3 |
| 3-D-Glucuronosyl-N2,6-disulfo-beta-D-glucosamine | 1.29 | 3.09E-02 | 399434.06 | 415370.04 | 312539.75 | 306721.15 | C12H21NO17S2 | level3 |
| 3-Hydroxy-3-methyl-2-oxobutanoic acid | 1.60 | 1.67E-06 | 87354804.44 | 91733263.39 | 73403825.21 | 78547777.94 | C5H8O4 | level3 |
| 3-Indoleacrylate | 1.83 | 1.67E-06 | 16491504.83 | 22335252.62 | 19097971.59 | 16690961.04 | C11H9NO2 | level3 |
| 3-Indoleacrylate | 1.62 | 6.46E-05 | 82465455.65 | 103661424.80 | 88525036.70 | 82585777.57 | C11H9NO2 | level3 |
| 3-Ketosphingosine | 1.32 | 2.63E-02 | 706989.16 | 593123.12 | 479088.63 | 577080.19 | C18H35NO2 | level3 |
| 4-(L-Alanin-3-yl)-2-hydroxy-cis,cis-muconate 6-semialdehyde | 1.77 | 1.67E-06 | 647049.63 | 571098.87 | 855587.69 | 736435.17 | C9H11NO6 | level3 |
| 4-(L-Alanin-3-yl)-2-hydroxy-cis,cis-muconate 6-semialdehyde | 1.66 | 1.39E-05 | 8234049.55 | 7097221.91 | 10096887.42 | 8633181.47 | C9H11NO6 | level3 |
| 4,5-Dioxopentanoate | 1.41 | 1.93E-04 | 25571415.30 | 28613577.78 | 24010006.98 | 23428586.86 | C5H6O4 | level3 |
| 4,5-seco-Dopa | 1.77 | 1.67E-06 | 647049.63 | 571098.87 | 855587.69 | 736435.17 | C9H11NO6 | level3 |
| 4,5-seco-Dopa | 1.66 | 1.39E-05 | 8234049.55 | 7097221.91 | 10096887.42 | 8633181.47 | C9H11NO6 | level3 |
| 4-Amino-4-deoxychorismate | 1.10 | 2.63E-02 | 1157079.51 | 1543719.02 | 1477322.95 | 1033072.08 | C10H11NO5 | level3 |
| 4-Hydroxy-2-oxopentanoate | 1.60 | 1.67E-06 | 87354804.44 | 91733263.39 | 73403825.21 | 78547777.94 | C5H8O4 | level3 |
| 4-Hydroxy-2-quinolone | 1.03 | 6.10E-03 | 10312606.18 | 13075877.91 | 9074921.96 | 10119763.59 | C9H7NO2 | level3 |
| 4-Hydroxyphenylacetylglutamic acid | 1.44 | 1.93E-04 | 36775535.93 | 41162580.62 | 34800002.99 | 34633832.36 | C13H15NO6 | level3 |
| 4-Imidazolone-5-propanoate | 1.60 | 1.20E-02 | 1247847.92 | 970790.51 | 1066635.08 | 1388810.91 | C6H8N2O3 | level3 |
| 4-Imidazolone-5-propanoate | 1.74 | 1.66E-03 | 276308583.22 | 151951045.08 | 35297207.24 | 117876708.17 | C6H8N2O3 | level3 |
| 5-(L-Alanin-3-yl)-2-hydroxy-cis,cis-muconate 6-semialdehyde | 1.77 | 1.67E-06 | 647049.63 | 571098.87 | 855587.69 | 736435.17 | C9H11NO6 | level3 |

| | | | | | | | | |
|---|---|---|---|---|---|---|---|---|
| 5-(L-Alanin-3-yl)-2-hydroxy-cis,cis-muconate 6-semialdehyde | 1.66 | 1.39E-05 | 8234049.55 | 7097221.91 | 10096887.42 | 8633181.47 | C9H11NO6 | level3 |
| 5-Acetylamino-6-formylamino-3-methyluracil | 1.28 | 6.96E-03 | 78934308.39 | 82105537.13 | 79884516.52 | 78754113.41 | C8H10N4O4 | level3 |
| 5-Hydroxyisourate | 1.24 | 1.07E-02 | 44980971.15 | 53977321.67 | 46214812.62 | 43581540.67 | C5H4N4O4 | level3 |
| 5-Oxo-D-proline | 1.68 | 1.93E-03 | 31725222.40 | 33866587.32 | 35054681.07 | 30247523.01 | C5H7NO3 | level3 |
| 5'-S-Methyl-5'-thioinosine | 1.73 | 1.39E-05 | 3289804.12 | 5490077.26 | 3135023.29 | 2949336.25 | C11H14N4O4S | level3 |
| 5-Ureido-4-imidazole carboxylate | 1.55 | 4.17E-02 | 1181412.88 | 1371969.48 | 1265175.78 | 1095868.33 | C5H6N4O3 | level3 |
| 6-Phospho-beta-D-glucosyl-(1,4)-D-glucose | 1.27 | 4.15E-04 | 6555938.04 | 7574882.77 | 6133343.76 | 6160178.51 | C12H23O14P | level3 |
| 9,10-Dihydroxystearate | 1.20 | 1.20E-02 | 23669245.24 | 16578776.07 | 27910288.79 | 25104759.23 | C18H36O4 | level3 |
| Acetyl-maltose | 1.33 | 4.08E-04 | 15208854.49 | 19321573.49 | 14842528.94 | 14400033.36 | C14H24O12 | level3 |
| Alanopine | 1.40 | 1.93E-04 | 3483342.66 | 4216541.98 | 3646274.78 | 3482061.94 | C6H11NO4 | level3 |
| alpha,alpha'-Trehalose 6-phosphate | 1.27 | 4.15E-04 | 6555938.04 | 7574882.77 | 6133343.76 | 6160178.51 | C12H23O14P | level3 |
| alpha-Maltose 1-phosphate | 1.27 | 4.15E-04 | 6555938.04 | 7574882.77 | 6133343.76 | 6160178.51 | C12H23O14P | level3 |
| Aspirin | 1.34 | 4.08E-04 | 18039127.34 | 27144639.48 | 12153406.22 | 16831036.41 | C9H8O4 | level3 |
| beta-Alanopine | 1.40 | 1.93E-04 | 3483342.66 | 4216541.98 | 3646274.78 | 3482061.94 | C6H11NO4 | level3 |
| beta-Citryl-L-glutamate | 1.54 | 6.46E-05 | 3222116.64 | 4626481.54 | 4452422.24 | 2871660.57 | C11H15NO10 | level3 |
| beta-D-Fructofuranosyl-alpha-D-mannopyranoside 6F-phosphate | 1.27 | 4.15E-04 | 6555938.04 | 7574882.77 | 6133343.76 | 6160178.51 | C12H23O14P | level3 |
| Betalamic acid | 1.66 | 1.39E-05 | 8234049.55 | 7097221.91 | 10096887.42 | 8633181.47 | C9H9NO5 | level3 |
| Betalamic acid | 1.19 | 4.17E-02 | 17933858.58 | 18118632.80 | 18384851.54 | 17670943.76 | C9H9NO5 | level3 |
| CDP-choline | 1.04 | 1.07E-02 | 1724486.03 | 1265563.20 | 1894110.45 | 1980418.46 | C14H26N4O11P2 | level3 |
| cis-9,10-Epoxystearic acid | 1.81 | 1.93E-04 | 11559703.23 | 17988377.94 | 9716772.01 | 12042192.57 | C18H34O3 | level3 |
| cis-9,10-Epoxystearic acid | 1.20 | 1.20E-02 | 23669245.24 | 16578776.07 | 27910288.79 | 25104759.23 | C18H34O3 | level3 |
| D-Erythrulose | 1.30 | 4.08E-04 | 1011031644.17 | 1072831552.45 | 1024748072.35 | 975644318.77 | C4H8O4 | level3 |
| D-Galactarate | 1.52 | 6.96E-03 | 13243689.57 | 18215245.79 | 20979513.93 | 14780452.91 | C6H10O8 | level3 |

| | | | | | | | | | |
|---|---|---|---|---|---|---|---|---|---|
| D-Galacturonate | 1.48 | 6.10E-03 | 10437303.67 | 11737607.79 | 9017953.41 | 8477135.86 | C6H10O7 | level3 |
| D-Glucarate | 1.52 | 6.96E-03 | 13243689.57 | 18215245.79 | 20979513.93 | 14780452.91 | C6H10O8 | level3 |
| D-Glucose | 1.59 | 1.67E-06 | 7449952.54 | 7920760.17 | 6074390.97 | 6527000.84 | C6H12O6 | level3 |
| D-Glucose/alpha-D-Galactose | 1.59 | 1.67E-06 | 7449952.54 | 7920760.17 | 6074390.97 | 6527000.84 | C6H12O6 | level3 |
| D-Glucose/beta-D-Glucose | 1.59 | 1.67E-06 | 7449952.54 | 7920760.17 | 6074390.97 | 6527000.84 | C6H12O6 | level3 |
| D-Glucose/D-Fructose | 1.59 | 1.67E-06 | 7449952.54 | 7920760.17 | 6074390.97 | 6527000.84 | C6H12O6 | level3 |
| D-Glucose/D-Galactose | 1.59 | 1.67E-06 | 7449952.54 | 7920760.17 | 6074390.97 | 6527000.84 | C6H12O6 | level3 |
| D-Glutamate | 1.41 | 1.93E-04 | 25571415.30 | 28613577.78 | 24010006.98 | 23428586.86 | C5H9NO4 | level3 |
| D-Glutamine | 1.46 | 1.24E-02 | 4187581.25 | 3007663.28 | 4080214.63 | 4866724.67 | C5H10N2O3 | level3 |
| Dopamine quinone | 1.17 | 2.63E-02 | 1598790.51 | 1778740.20 | 1513632.24 | 1360268.75 | C8H9NO2 | level3 |
| Ethylenediaminetriacetic acid | 1.60 | 1.20E-02 | 1247847.92 | 970790.51 | 1066635.08 | 1388810.91 | C8H14N2O6 | level3 |
| Ethylenediaminetriacetic acid | 1.29 | 2.42E-02 | 373222429.43 | 515919265.92 | 530936915.71 | 384033059.22 | C8H14N2O6 | level3 |
| Formylisoglutamine | 1.48 | 1.66E-03 | 2092691.01 | 1525690.95 | 1910279.91 | 2334260.29 | C6H10N2O4 | level3 |
| gamma-L-Glutamyl-L-cysteine | 1.17 | 1.54E-02 | 6018519.91 | 8070869.30 | 7128765.49 | 4609036.55 | C8H14N2O5S | level3 |
| Glutamate | 1.41 | 1.93E-04 | 25571415.30 | 28613577.78 | 24010006.98 | 23428586.86 | C5H9NO4 | level3 |
| Glycochenodeoxycholate 7-sulfate | 1.08 | 6.96E-03 | 689073.62 | 1052177.60 | 860238.74 | 711625.70 | C26H43NO8S | level3 |
| IMP | 1.56 | 1.54E-02 | 883344.22 | 722195.85 | 783002.25 | 892337.68 | C10H13N4O8P | level3 |
| Isoglutamine | 1.46 | 1.24E-02 | 4187581.25 | 3007663.28 | 4080214.63 | 4866724.67 | C5H10N2O3 | level3 |
| Isoglutamine | 1.24 | 1.07E-02 | 44980971.15 | 53977321.67 | 46214812.62 | 43581540.67 | C5H10N2O3 | level3 |
| Isoniazid alpha-ketoglutaric acid | 1.55 | 6.52E-08 | 2578451.66 | 1684643.49 | 4189885.84 | 3801024.97 | C11H11N3O5 | level3 |
| L-1-Pyrroline-3-hydroxy-5-carboxylate | 1.68 | 1.93E-03 | 31725222.40 | 33866587.32 | 35054681.07 | 30247523.01 | C5H7NO3 | level3 |
| L-2,4-Diaminobutanoate | 1.19 | 2.42E-02 | 88616224.21 | 96016293.23 | 99293351.85 | 78537723.79 | C4H10N2O2 | level3 |
| L-2-Amino-3-oxobutanoic acid | 1.06 | 1.93E-03 | 802059.65 | 946188.30 | 807658.72 | 776112.36 | C4H7NO3 | level3 |
| L-4-Hydroxyglutamate semialdehyde | 1.41 | 1.93E-04 | 25571415.30 | 28613577.78 | 24010006.98 | 23428586.86 | C5H9NO4 | level3 |
| L-Alanyl-D-glutamate | 1.08 | 4.17E-02 | 1611214.57 | 1755171.30 | 1430002.11 | 1181730.73 | C8H14N2O5 | level3 |
| L-Altarate | 1.52 | 6.96E-03 | 13243689.57 | 18215245.79 | 20979513.93 | 14780452.91 | C6H10O8 | level3 |
| L-Aspartate 4-semialdehyde | 1.06 | 1.93E-03 | 802059.65 | 946188.30 | 807658.72 | 776112.36 | C4H7NO3 | level3 |

| | | | | | | | | | |
|---|---|---|---|---|---|---|---|---|---|
| L-Cystine | 1.40 | 4.50E-03 | 14717113.28 | 20284465.10 | 18683253.93 | 13815080.14 | C6H12N2O4S2 | level3 |
| L-Mimosine | 1.49 | 6.10E-03 | 1188043.14 | 959825.25 | 1147947.74 | 1314032.24 | C8H10N2O4 | level3 |
| Lotaustralin | 1.23 | 4.17E-02 | 8009238.99 | 8413186.73 | 9272130.86 | 7400503.61 | C11H19NO6 | level3 |
| L-Serine-phosphoethanolamine | 1.11 | 4.17E-02 | 183351.74 | 152157.32 | 186769.54 | 230161.75 | C5H13N2O6P | level3 |
| Maltose 6'-phosphate | 1.27 | 4.15E-04 | 6555938.04 | 7574882.77 | 6133343.76 | 6160178.51 | C12H23O14P | level3 |
| Methyl cinnamate | 1.60 | 5.03E-08 | 2991001.99 | 2249308.36 | 7394874.67 | 5038866.36 | C10H10O2 | level3 |
| N-(Carboxymethyl)-D-alanine | 1.41 | 1.93E-04 | 25571415.30 | 28613577.78 | 24010006.98 | 23428586.86 | C5H9NO4 | level3 |
| N5-Hydroxy-L-ornithine | 1.45 | 4.17E-02 | 73364795.81 | 90283350.10 | 70970387.60 | 45299820.45 | C5H12N2O3 | level3 |
| N-Acetyl-L-glutamate | 1.66 | 1.39E-05 | 8234049.55 | 7097221.91 | 10096887.42 | 8633181.47 | C7H11NO5 | level3 |
| N-Acetylneuraminate | 1.26 | 6.96E-03 | 11674821.95 | 12350734.62 | 13852042.11 | 12129306.10 | C11H19NO9 | level3 |
| N-Formiminoglycine | 1.21 | 3.27E-02 | 4951710.95 | 4549803.76 | 4662760.46 | 4840689.48 | C3H6N2O2 | level3 |
| N-Formyl-L-aspartate | 1.35 | 3.27E-02 | 654501.32 | 543569.63 | 567547.09 | 645107.42 | C5H7NO5 | level3 |
| N-Glucosylnicotinate | 1.03 | 2.63E-02 | 339366.94 | 529091.30 | 329664.52 | 223719.30 | C12H16NO7 | level3 |
| Nicotinate D-ribonucleoside | 1.37 | 1.93E-04 | 4036686.24 | 3300327.70 | 7102911.72 | 5604428.45 | C11H14NO6 | level3 |
| Nonane-4,6-dione | 1.57 | 1.67E-06 | 5451380.05 | 5630000.26 | 4954834.93 | 5205826.26 | C9H16O2 | level3 |
| N-Succinyl-LL-2,6-diaminoheptanedioate | 1.11 | 3.27E-02 | 1128853.69 | 923921.25 | 562821.60 | 730938.30 | C11H18N2O7 | level3 |
| O-Acetyl-L-serine | 1.41 | 1.93E-04 | 25571415.30 | 28613577.78 | 24010006.98 | 23428586.86 | C5H9NO4 | level3 |
| Phosphocreatine | 1.54 | 1.39E-05 | 1540980.33 | 2271084.61 | 1985575.73 | 1442010.74 | C4H10N3O5P | level3 |
| Porphobilinogen | 1.62 | 9.88E-06 | 662352.98 | 900467.42 | 637921.07 | 625496.71 | C10H14N2O4 | level3 |
| Sphingosyl-phosphocholine | 1.19 | 3.07E-07 | 2489215.10 | 2300010.77 | 4777876.93 | 3658308.09 | C23H50N2O5P | level3 |
| Sucrose 6'-phosphate | 1.27 | 4.15E-04 | 6555938.04 | 7574882.77 | 6133343.76 | 6160178.51 | C12H23O14P | level3 |
| Sucrose 6-phosphate | 1.27 | 4.15E-04 | 6555938.04 | 7574882.77 | 6133343.76 | 6160178.51 | C12H23O14P | level3 |
| Thiamin monophosphate | 1.27 | 1.93E-03 | 2801658.99 | 2986193.62 | 2573964.07 | 2625122.69 | C12H18N4O4PS | level3 |

VIP: variable importance on projection.

FDR P.value: P.value of FDR-adjusted Wilcoxon–Mann–Whitney test

Mean-XXX: average value for each group
