# Supplementary material for: Identifying plasma metabolic characteristics of major depressive disorder, bipolar disorder, and schizophrenia in adolescents
Source: Transl Psychiatry. 2024 Mar 26;14:163. doi: 10.1038/s41398-024-02886-z (PMC10966062; doi:10.1038/s41398-024-02886-z)
Supplement: Supplementary file 9 — Supplementary Table 3 [file 41398_2024_2886_MOESM9_ESM.pdf]

Supplementary Table 3A: Detailed information of differentially expressed metabolites from the disorder-HC comparisons (MDD-HC).

Name	Biomarker	Vip	FC	FDR P.value	Formula	Confidence_level
Pyruvic acid	YES	1.79	1.43	1.20E-02	C3H4O3	level1
Car(11:1)_RT383	YES	2.36	0.49	8.80E-05	C18H33NO4	level1
5-Aminolevulinic acid	YES	1.62	0.73	1.68E-02	C5H9NO3	level1
Acetoin	YES	2.30	18.48	2.28E-02	C4H8O2	level1
Capric acid	YES	1.53	0.71	1.81E-02	C10H20O2	level1
Salicylic acid	YES	1.56	0.59	2.28E-02	C7H6O3	level1
Car(9:0)_RT346	YES	2.09	0.46	8.80E-05	C16H31NO4	level1
Car(3:0)_RT63	NO	1.63	0.77	1.20E-02	C10H19NO4	level1
Car(12:0)_RT430	NO	1.92	0.48	1.78E-04	C19H37NO4	level1
Car(14:0)_RT467	NO	1.82	0.66	6.37E-03	C21H41NO4	level1
Adenosine 3',5'-cyclic phosphate (cAMP)	NO	1.66	0.77	1.07E-03	C10H12N5O6P	level1
Car(10:1)_RT357	NO	1.74	0.59	2.89E-03	C17H31NO4	level1
Eicosapentaenoic acid	NO	1.75	0.65	6.37E-03	C20H30O2	level1
Arachidonic acid (AA)	NO	2.00	0.71	2.89E-03	C20H32O2	level1
Docosahexaenoic acid (DHA)	NO	1.99	0.66	2.89E-03	C22H32O2	level1
Car(12:1)_RT402	NO	1.68	0.55	1.20E-02	C19H35NO4	level1
Car(14:1)_RT447	NO	1.61	0.55	1.20E-02	C21H39NO4	level1
3-methylcytidine	NO	1.61	0.79	2.28E-02	C10H15N3O5	level1
Car(14:1)_RT438	NO	1.61	0.55	1.20E-02	C21H39NO4	level1
Biliverdin	NO	1.56	0.63	1.81E-02	C33H34N4O6	level1
N1-Methyl-4-pyridone-3-carboxamide	NO	1.38	0.71	1.81E-02	C7H8N2O2	level1
Car(12:2)_RT382	NO	1.93	0.61	3.19E-03	C19H33NO4	level1
cis-8,11,14-Eicosatrienoic acid	NO	1.78	0.66	1.20E-02	C20H34O2	level1
gamma-Glutamyllysine	NO	1.96	0.64	6.37E-03	C11H21N3O5	level1

Car(16:3)_RT442	NO	2.04	0.59	2.89E-03	C23H39NO4	level1
Car(16:4)_RT421	NO	2.14	0.49	6.95E-04	C23H37NO4	level1
Car(14:1-O)_RT416	NO	1.90	0.49	5.47E-03	C21H39NO5	level1
Methionine	NO	1.96	0.78	6.95E-04	C5H11NO2S	level1
Arginine	NO	1.95	0.75	2.89E-03	C6H14N4O2	level1
Car(16:1-O)_RT453	NO	1.80	0.61	1.20E-02	C23H43NO5	level1
Xanthine	NO	1.58	0.77	6.37E-03	C5H4N4O2	level1
Kynurenine	NO	1.92	0.77	6.37E-03	C10H12N2O3	level1
Lysine	NO	1.86	0.71	5.98E-03	C6H14N2O2	level1
Car(14:2-O)_RT389	NO	1.95	0.53	2.89E-03	C21H37NO5	level1
Deoxycholic acid	NO	1.63	0.55	1.20E-02	C24H40O4	level1
Car(16:2-O)_RT395	NO	1.64	0.67	1.20E-02	C23H41NO5	level1
Histidinol	NO	1.72	0.79	1.20E-02	C6H11N3O	level1
Chenodeoxycholic acid	NO	1.63	0.55	1.20E-02	C24H40O4	level1
Car(6:0)_RT250	NO	1.82	0.66	1.20E-02	C13H25NO4	level1
Cortisone/Aldosterone	NO	1.97	0.73	6.95E-04	C21H28O5	level1
Cortisone/Aldosterone	NO	1.97	0.73	6.95E-04	C21H28O5	level1
Car(6:0)_RT263	NO	1.82	0.66	1.20E-02	C13H25NO4	level1
Car(8:0)_RT316	NO	1.92	0.49	5.14E-04	C15H29NO4	level1
Car(10:0)_RT370	NO	2.02	0.45	1.78E-04	C17H33NO4	level1
Car(11:0)_RT390	NO	1.84	0.36	8.77E-06	C18H35NO4	level1
Car(15:1-O)_RT438	NO	2.53	0.56	3.19E-03	C22H41NO5	level2
Car(11:1-O2)_RT289	NO	2.08	0.57	2.89E-03	C18H31NO6	level2
Car(7:0)_RT288	NO	1.94	0.67	6.95E-04	C14H27NO4	level2
Car(13:0)_RT433	NO	1.52	0.62	6.37E-03	C20H39NO4	level2
Car(14:2)_RT416	NO	1.62	0.61	1.20E-02	C21H37NO4	level2

Car(12:1-O2)_RT315	NO	1.84	0.58	1.20E-02	C19H33NO6	level2
Car(16:1-O2)_RT414	NO	1.92	0.64	3.19E-03	C23H41NO6	level2
4-Hydroxyglutamic acid	NO	1.62	1.34	2.75E-02	C5H9NO5	level2
Cysteine	NO	1.74	0.71	3.19E-03	C3H7NO2S	level2
(R)-10-Hydroxystearate	NO	1.87	0.71	5.47E-03	C18H36O3	level3
3-Indoleacrylate	NO	2.33	0.74	8.80E-05	C11H9NO2	level3
cis-9,10-Epoxystearic acid	NO	1.80	0.64	1.56E-02	C18H34O3	level3
18-Hydroxyoleate	NO	1.80	0.64	1.56E-02	C18H34O3	level3
16-Oxopalmitate	NO	1.98	1.66	1.07E-03	C16H30O3	level3
3-Indoleacrylate	NO	2.17	0.80	5.14E-04	C11H9NO2	level3
Porphobilinogen	NO	2.09	0.74	6.95E-04	C10H14N2O4	level3
18-Oxooleate	NO	1.73	0.69	2.75E-02	C18H32O3	level3
Formylisoglutamine	NO	1.62	1.37	1.20E-02	C6H10N2O4	level3
15,16-Dihydrobiliverdin	NO	1.74	0.51	1.07E-03	C33H36N4O6	level3
Phosphocreatine	NO	1.91	0.68	6.95E-04	C4H10N3O5P	level3
beta-Citryl-L-glutamate	NO	1.54	0.70	1.20E-02	C11H15NO10	level3
5'-S-Methyl-5'-thioinosine	NO	1.96	0.60	1.07E-03	C11H14N4O4S	level3
Acetyl-maltose	NO	1.71	0.79	1.20E-02	C14H24O12	level3
L-Cystine	NO	1.69	0.73	1.20E-02	C6H12N2O4S2	level3
(3Z)-Phytochromobilin	NO	1.74	0.51	1.07E-03	C33H36N4O6	level3
Miraxanthin-V	NO	1.11	0.58	1.20E-02	C17H18N2O6	level3

VIP: variable importance on projection; FC: fold change of disorder / HC; FDR P.value: P.value of FDR-adjusted Wilcoxon–Mann–Whitney test.

Supplementary Table 3B: Detailed information of differentially expressed metabolites from the disorder-HC comparisons(BD-HC).

Name	Biomarker	Vip	FC	FDR P.value	Formula	Confidence_level
Glucuronic acid	YES	2.03	1.30	1.02E-02	C6H10O7	level1
3-methylcytidine	YES	2.42	0.62	8.82E-06	C10H15N3O5	level1
Acetoin	YES	2.56	19.37	6.50E-03	C4H8O2	level1
Car(12:2)_RT382	YES	2.08	0.48	5.75E-05	C19H33NO4	level1
Nordeoxycholic acid	YES	2.13	0.52	3.21E-04	C23H38O4	level1
Methionine	YES	1.97	0.76	3.21E-04	C5H11NO2S	level1
Cortisone/Aldosterone	YES	2.08	0.68	5.75E-05	C21H28O5	level1
Cortisone/Aldosterone	YES	2.08	0.68	5.75E-05	C21H28O5	level1
Car(3:0)_RT63	NO	1.59	0.75	1.58E-03	C10H19NO4	level1
Car(12:0)_RT430	NO	1.30	0.61	2.90E-03	C19H37NO4	level1
Adenosine 3',5'-cyclic phosphate (cAMP)	NO	1.77	0.72	1.34E-04	C10H12N5O6P	level1
Phenylpyruvic acid	NO	1.54	0.74	2.90E-03	C9H8O3	level1
2-Ketocaproic acid	NO	1.38	0.78	4.80E-03	C6H10O3	level1
Car(10:1)_RT357	NO	1.23	0.65	2.90E-03	C17H31NO4	level1
3-Methyl-2-oxovaleric acid	NO	1.38	0.78	4.80E-03	C6H10O3	level1
Car(11:1)_RT383	NO	2.13	0.49	8.82E-06	C18H33NO4	level1
Indolelactic acid	NO	1.21	0.75	2.90E-03	C11H11NO3	level1
Arachidonic acid (AA)	NO	1.75	0.70	4.80E-03	C20H32O2	level1
Docosahexaenoic acid (DHA)	NO	1.30	0.75	4.38E-02	C22H32O2	level1
Car(12:1)_RT402	NO	1.27	0.60	1.83E-02	C19H35NO4	level1
Ketoleucine	NO	1.38	0.78	4.80E-03	C6H10O3	level1
Erucamide	NO	1.53	2.03	1.83E-02	C22H43NO	level1
Mesaconic acid	NO	1.06	0.71	4.38E-02	C5H6O4	level1
gamma-Glutamylvaline	NO	1.67	0.78	4.80E-03	C10H18N2O5	level1

N1-Methyl-4-pyridone-3-carboxamide	NO	1.49	0.67	6.50E-03	C7H8N2O2	level1
gamma-Glutamyllysine	NO	1.95	0.61	1.19E-03	C11H21N3O5	level1
3alpha-Hydroxy-6-oxo-5alpha-cholan-24-oic acid	NO	1.41	0.66	1.77E-02	C24H38O4	level1
Dehydrolithocholic acid	NO	1.29	0.77	1.77E-02	C24H38O3	level1
3alpha-Hydroxy-12-oxo-5beta-cholan-24-oic acid	NO	1.41	0.66	1.77E-02	C24H38O4	level1
Apocholic acid	NO	1.41	0.66	1.77E-02	C24H38O4	level1
Allolithocholic acid	NO	1.28	0.64	4.80E-03	C24H40O3	level1
4-Vinylphenol	NO	1.84	0.64	2.90E-03	C8H8O	level1
Isovalerylcarnitine	NO	1.42	0.77	1.83E-02	C12H23NO4	level1
Car(16:3)_RT442	NO	1.43	0.69	1.83E-02	C23H39NO4	level1
Tyrosine O-sulfate	NO	1.86	0.76	3.21E-04	C9H11NO6S	level1
Malic acid	NO	1.12	0.78	2.90E-03	C4H6O5	level1
Xanthurenic acid	NO	1.37	0.71	1.83E-02	C10H7NO4	level1
Car(12:1-O)_RT364	NO	1.53	0.59	2.90E-03	C19H35NO5	level1
Car(14:1-O)_RT416	NO	1.71	0.48	1.19E-03	C21H39NO5	level1
Aspartic acid	NO	1.21	0.78	1.83E-02	C4H7NO4	level1
Arginine	NO	2.35	0.64	8.82E-06	C6H14N4O2	level1
Tyrosine	NO	1.91	0.78	1.19E-03	C9H11NO3	level1
Car(16:1-O)_RT453	NO	1.32	0.68	4.27E-02	C23H43NO5	level1
Lactic acid	NO	1.47	1.52	1.77E-02	C3H6O3	level1
Kynurenic acid	NO	1.56	0.68	1.17E-03	C10H7NO3	level1
Kynurenine	NO	1.78	0.76	2.90E-03	C10H12N2O3	level1
Lysine	NO	1.80	0.68	1.19E-03	C6H14N2O2	level1
Car(14:2-O)_RT389	NO	1.79	0.50	3.21E-04	C21H37NO5	level1

Car(16:2-O)_RT395	NO	1.81	0.56	1.19E-03	C23H41NO5	level1
2-Aminooctanoic acid	NO	1.44	0.71	1.83E-02	C8H17NO2	level1
Histidinol	NO	1.75	0.73	1.19E-03	C6H11N3O	level1
Urocanic acid	NO	1.36	0.76	1.83E-02	C6H6N2O2	level1
5-Aminopentanoic acid	NO	2.11	0.79	5.75E-05	C5H11NO2	level1
Lithocholic acid	NO	1.28	0.64	4.80E-03	C24H40O3	level1
gamma-Glutamylalanine	NO	1.18	0.62	1.83E-02	C8H14N2O5	level1
Cysteic acid	NO	1.42	1.31	4.27E-02	C3H7NO5S	level1
Salicylic acid	NO	1.14	0.59	4.90E-04	C7H6O3	level1
Homoarginine	NO	1.51	0.80	4.80E-03	C7H16N4O2	level1
2-Hydroxyoctanoic acid	NO	1.43	0.74	1.77E-02	C8H16O3	level1
Car(9:0)_RT346	NO	1.47	0.55	6.50E-04	C16H31NO4	level1
Car(11:0)_RT390	NO	1.46	0.44	8.82E-06	C18H35NO4	level1
Car(15:1-O)_RT438	NO	2.56	0.51	3.21E-04	C22H41NO5	level2
Lactaldehyde	NO	1.80	0.78	3.21E-04	C3H6O2	level2
Car(5:1)_RT214	NO	1.56	0.70	1.06E-02	C12H21NO4	level2
Car(9:1)_RT320	NO	1.40	0.72	2.90E-03	C16H29NO4	level2
Car(10:3-O)_RT335	NO	1.48	0.64	1.83E-02	C17H27NO5	level2
Car(16:1-O2)_RT414	NO	1.90	0.57	1.34E-04	C23H41NO6	level2
4-Hydroxyglutamic acid	NO	1.57	1.36	1.77E-02	C5H9NO5	level2
Ursocholic acid	NO	1.37	0.75	4.38E-02	C24H40O5	level2
alpha-Muricholic acid	NO	1.37	0.75	4.38E-02	C24H40O5	level2
beta-Muricholic acid	NO	1.37	0.75	4.38E-02	C24H40O5	level2
omega-Muricholic acid	NO	1.37	0.75	4.38E-02	C24H40O5	level2
Cysteine	NO	1.67	0.68	1.19E-03	C3H7NO2S	level2
3-Methylhistidine	NO	1.16	0.55	1.83E-02	C7H11N3O2	level2

4-(L-Alanin-3-yl)-2-hydroxy-cis,cis-muconate 6-semialdehyde	NO	1.73	1.29	1.77E-02	C9H11NO6	level3
2,6-Dihydroxynicotinate	NO	2.39	0.77	5.75E-05	C6H5NO4	level3
4-Amino-4-deoxychorismate	NO	1.29	0.67	6.50E-03	C10H11NO5	level3
Ethylenediaminetriacetic acid	NO	1.71	1.43	6.50E-03	C8H14N2O6	level3
N5-Hydroxy-L-ornithine	NO	1.54	0.50	4.80E-03	C5H12N2O3	level3
(R)-10-Hydroxystearate	NO	1.65	0.72	2.90E-03	C18H36O3	level3
4,5-seco-Dopa	NO	1.73	1.29	1.77E-02	C9H11NO6	level3
3-Indoleacrylate	NO	2.05	0.75	3.21E-04	C11H9NO2	level3
3-Indoleacrylate	NO	1.93	0.80	3.21E-04	C11H9NO2	level3
Dopamine quinone	NO	1.85	0.76	4.80E-03	C8H9NO2	level3
5-Ureido-4-imidazole carboxylate	NO	1.81	0.80	1.77E-02	C5H6N4O3	level3
Porphobilinogen	NO	2.11	0.69	3.21E-04	C10H14N2O4	level3
2-Dehydro-3-deoxy-D-glucarate	NO	1.50	0.73	1.83E-02	C6H8O7	level3
5-Dehydro-4-deoxy-D-glucarate	NO	1.50	0.73	1.83E-02	C6H8O7	level3
gamma-L-Glutamyl-L-cysteine	NO	1.55	0.57	2.90E-03	C8H14N2O5S	level3
CDP-choline	NO	1.65	1.56	4.80E-03	C14H26N4O11P2	level3
L-beta-Phenylalanine	NO	1.38	0.62	1.83E-02	C9H11NO2	level3
D-beta-Phenylalanine	NO	1.38	0.62	1.83E-02	C9H11NO2	level3
L-Serine-phosphoethanolamine	NO	1.50	1.51	6.50E-03	C5H13N2O6P	level3
2-Hydroxyethylenedicarboxylate	NO	1.50	0.73	1.83E-02	C4H4O5	level3
9,10-Dihydroxystearate	NO	1.55	1.51	6.50E-03	C18H36O4	level3
L-Alanyl-D-glutamate	NO	1.57	0.67	2.90E-03	C8H14N2O5	level3
L-1-Pyrroline-3-hydroxy-5-carboxylate	NO	1.24	1.46	1.83E-02	C5H7NO3	level3
Formylisoglutamine	NO	1.85	1.53	3.21E-04	C6H10N2O4	level3
L-Mimosine	NO	1.96	1.37	1.19E-03	C8H10N2O4	level3

D-Galactaro-1,4-lactone	NO	1.50	0.73	1.83E-02	C6H8O7	level3
D-Glucaro-1,4-lactone	NO	1.50	0.73	1.83E-02	C6H8O7	level3
D-Galactaro-1,5-lactone	NO	1.50	0.73	1.83E-02	C6H8O7	level3
D-Glucaro-1,5-lactone	NO	1.50	0.73	1.83E-02	C6H8O7	level3
Nicotinurate	NO	1.27	0.69	1.83E-02	C8H8N2O3	level3
D-Glutamine	NO	1.79	1.62	1.19E-03	C5H10N2O3	level3
Acetyl-CoA	NO	1.30	0.55	1.83E-02	C23H38N7O17P3S	level3
Isoglutamine	NO	1.79	1.62	1.19E-03	C5H10N2O3	level3
D-Galacturonate	NO	1.73	0.72	2.90E-03	C6H10O7	level3
1-Pyrroline-4-hydroxy-2-carboxylate	NO	1.24	1.46	1.83E-02	C5H7NO3	level3
5-(L-Alanin-3-yl)-2-hydroxy-cis,cis-muconate 6-semialdehyde	NO	1.73	1.29	1.77E-02	C9H11NO6	level3
Aspirin	NO	1.30	0.62	1.83E-02	C9H8O4	level3
Isoniazid alpha-ketoglutaric acid	NO	1.83	2.26	5.75E-05	C11H11N3O5	level3
Methyl cinnamate	NO	1.52	2.24	2.90E-03	C10H10O2	level3
15,16-Dihydrobiliverdin	NO	1.08	0.63	6.50E-03	C33H36N4O6	level3
Carboxymethyloxysuccinate	NO	1.50	0.73	1.83E-02	C6H8O7	level3
D-Phenylalanine	NO	1.38	0.62	1.83E-02	C9H11NO2	level3
3-D-Glucuronosyl-N2,6-disulfo-beta-D-glucosamine	NO	1.26	0.74	1.83E-02	C12H21NO17S2	level3
N-Glucosylnicotinate	NO	1.31	0.42	4.80E-03	C12H16NO7	level3
Phosphocreatine	NO	1.98	0.63	5.75E-05	C4H10N3O5P	level3
beta-Citryl-L-glutamate	NO	1.74	0.62	1.19E-03	C11H15NO10	level3
(4R,5S)-4,5,6-Trihydroxy-2,3-dioxohexanoate	NO	1.50	0.73	1.83E-02	C6H8O7	level3
Glycochenodeoxycholate 7-sulfate	NO	1.17	0.68	6.50E-03	C26H43NO8S	level3
5'-S-Methyl-5'-thioinosine	NO	1.96	0.54	5.75E-05	C11H14N4O4S	level3

4-Hydroxy-2-quinolone	NO	1.20	0.77	4.27E-02	C9H7NO2	level3
4-Imidazolone-5-propanoate	NO	1.71	1.43	6.50E-03	C6H8N2O3	level3
4-Oxoproline	NO	1.24	1.46	1.83E-02	C5H7NO3	level3
Isonicotinylglycine	NO	1.27	0.69	1.83E-02	C8H8N2O3	level3
Ethylenediamine-N,N'-diacetic acid	NO	1.21	0.77	1.83E-02	C6H12N2O4	level3
Acetyl-maltose	NO	1.74	0.75	4.90E-04	C14H24O12	level3
D-Octopine	NO	1.74	0.75	4.80E-03	C9H18N4O4	level3
cis-9,10-Epoxystearic acid	NO	1.62	0.67	1.83E-02	C18H34O3	level3
cis-9,10-Epoxystearic acid	NO	1.55	1.51	6.50E-03	C18H34O3	level3
cis-9,10-Epoxystearic acid	NO	1.60	0.69	1.77E-02	C18H34O3	level3
D-Alanyl-D-serine	NO	1.21	0.77	1.83E-02	C6H12N2O4	level3
9,10-Epoxy-18-hydroxystearate	NO	1.29	0.78	1.77E-02	C18H34O4	level3
Oxaloacetate	NO	1.50	0.73	1.83E-02	C4H4O5	level3
3-(4-Hydroxyphenyl)pyruvate	NO	1.30	0.62	1.83E-02	C9H8O4	level3
Indole-3-carboxylate	NO	1.10	0.80	4.80E-03	C9H7NO2	level3
L-Cystine	NO	1.65	0.68	4.80E-03	C6H12N2O4S2	level3
18-Hydroxyoleate	NO	1.55	1.51	6.50E-03	C18H34O3	level3
18-Hydroxyoleate	NO	1.62	0.67	1.83E-02	C18H34O3	level3
(3Z)-Phytochromobilin	NO	1.08	0.63	6.50E-03	C33H36N4O6	level3
16-Oxopalmitate	NO	1.56	0.62	1.77E-02	C16H30O3	level3
16-Oxopalmitate	NO	1.43	1.79	2.90E-03	C16H30O3	level3
5-Oxo-D-proline	NO	1.24	1.46	1.83E-02	C5H7NO3	level3

VIP: variable importance on projection.

FDR P.value: P.value of FDR-adjusted Wilcoxon–Mann–Whitney test

FC: fold change of disorder / HC

.

Supplementary Table 3C: Detailed information of differentially expressed metabolites from the disorder-HC comparisons (SCZ-HC).

Name	Biomarker	Vip	FC	FDR P.value	Formula	Confidence_level
3-methylcytidine	YES	2.34	0.59	3.69E-07	C10H15N3O5	level1
Adenosine 3',5'-cyclic phosphate (cAMP)	YES	1.93	0.68	7.63E-06	C10H12N5O6P	level1
Car(11:1)_RT383	YES	2.07	0.42	2.44E-07	C18H33NO4	level1
Car(16:2-O)_RT395	YES	2.15	0.47	4.09E-05	C23H41NO5	level1
Car(9:0)_RT346	YES	2.42	0.25	8.08E-08	C16H31NO4	level1
Pyroglutamic acid	YES	2.82	1.48	8.08E-08	C5H7NO3	level1
Xanthurenic acid	YES	2.29	0.43	2.44E-07	C10H7NO4	level1
2-Aminoadipic acid	NO	1.59	0.70	9.26E-03	C6H11NO4	level1
4-Methylsalicylic acid	NO	1.36	0.35	4.91E-02	C8H8O3	level1
4-Vinylphenol	NO	2.11	0.48	4.09E-05	C8H8O	level1
5-Aminolevulinic acid	NO	1.11	0.75	4.91E-02	C5H9NO3	level1
Acetylcarnitine (Car(2:0))	NO	1.63	0.73	1.44E-03	C9H17NO4	level1
Arachidonic acid (AA)	NO	2.31	0.55	3.33E-07	C20H32O2	level1
Arginine	NO	2.35	0.61	6.74E-06	C6H14N4O2	level1
Aspartic acid	NO	1.43	0.68	1.16E-02	C4H7NO4	level1
Butyrylcarnitine	NO	1.49	0.71	9.26E-03	C11H21NO4	level1
Capric acid	NO	1.67	0.62	1.98E-04	C10H20O2	level1
Car(10:0)_RT370	NO	1.95	0.35	2.44E-07	C17H33NO4	level1
Car(10:1)_RT357	NO	1.82	0.48	7.63E-06	C17H31NO4	level1
Car(10:2)_RT333	NO	1.32	0.61	9.58E-03	C17H29NO4	level1
Car(10:2)_RT347	NO	1.32	0.61	9.58E-03	C17H29NO4	level1
Car(11:0)_RT390	NO	1.95	0.19	1.71E-09	C18H35NO4	level1
Car(12:0)_RT430	NO	2.02	0.33	2.06E-07	C19H37NO4	level1
Car(12:1)_RT402	NO	1.68	0.44	3.80E-04	C19H35NO4	level1

Car(12:1-O)_RT364	NO	1.93	0.45	2.38E-05	C19H35NO5	level1
Car(12:2)_RT382	NO	2.25	0.41	2.44E-07	C19H33NO4	level1
Car(12:2-O)_RT340	NO	1.54	0.51	9.26E-03	C19H33NO5	level1
Car(14:0)_RT467	NO	1.84	0.60	1.98E-04	C21H41NO4	level1
Car(14:1)_RT438	NO	1.54	0.47	1.44E-03	C21H39NO4	level1
Car(14:1)_RT447	NO	1.54	0.47	1.44E-03	C21H39NO4	level1
Car(14:1-O)_RT416	NO	1.92	0.36	7.63E-06	C21H39NO5	level1
Car(14:2-O)_RT389	NO	2.18	0.35	4.74E-07	C21H37NO5	level1
Car(16:1-O)_RT453	NO	1.84	0.52	1.44E-03	C23H43NO5	level1
Car(16:2)_RT456	NO	1.41	0.57	9.26E-03	C23H41NO4	level1
Car(16:3)_RT442	NO	1.94	0.53	9.02E-04	C23H39NO4	level1
Car(16:4)_RT421	NO	1.87	0.46	1.98E-04	C23H37NO4	level1
Car(18:1)	NO	1.49	1.34	2.60E-02	C25H47NO4	level1
Car(3:0)_RT63	NO	1.61	0.75	9.26E-03	C10H19NO4	level1
Car(4:0)_RT100	NO	1.49	0.71	9.26E-03	C11H21NO4	level1
Car(6:0)_RT250	NO	1.52	0.60	1.40E-04	C13H25NO4	level1
Car(6:0)_RT263	NO	1.52	0.60	1.40E-04	C13H25NO4	level1
Car(8:0)_RT316	NO	1.88	0.38	6.74E-06	C15H29NO4	level1
cis-8,11,14-Eicosatrienoic acid	NO	1.56	0.64	9.26E-03	C20H34O2	level1
Cortisone/Aldosterone	NO	1.71	0.72	1.44E-03	C21H28O5	level1
Cortisone/Aldosterone	NO	1.71	0.72	1.44E-03	C21H28O5	level1
Dehydrolithocholic acid	NO	1.04	0.79	4.91E-02	C24H38O3	level1
Dihydroxyacetone	NO	2.05	1.68	6.74E-06	C3H6O3	level1
Dimethylglycine	NO	1.36	1.41	8.11E-03	C4H9NO2	level1
Docosahexaenoic acid (DHA)	NO	2.04	0.57	1.40E-04	C22H32O2	level1
Dodecanoic acid	NO	1.80	0.47	1.44E-03	C12H24O2	level1

Eicosapentaenoic acid	NO	1.88	0.54	9.02E-04	C20H30O2	level1
Epinephrine	NO	1.92	3.76	2.94E-03	C9H13NO3	level1
Glutamic acid	NO	1.71	1.45	9.26E-03	C5H9NO4	level1
Glyceraldehyde	NO	2.05	1.68	6.74E-06	C3H6O3	level1
Glycerophosphocholine	NO	1.90	1.27	2.94E-03	C8H20NO6P	level1
Heptadecanoic acid	NO	1.60	0.63	9.26E-03	C17H34O2	level1
Histamine	NO	1.48	0.60	9.26E-03	C5H9N3	level1
Indole-3-pyruvic acid	NO	1.34	0.68	1.79E-02	C11H9NO3	level1
Indolelactic acid	NO	1.30	0.72	8.11E-03	C11H11NO3	level1
Isovalerylcarnitine	NO	1.36	0.73	2.60E-02	C12H23NO4	level1
Kynurenic acid	NO	1.78	0.64	9.02E-04	C10H7NO3	level1
Kynurenine	NO	1.66	0.78	4.14E-03	C10H12N2O3	level1
Lactic acid	NO	2.34	1.81	4.74E-07	C3H6O3	level1
Myristic acid	NO	1.48	0.71	4.91E-02	C14H28O2	level1
N1-Methyl-4-pyridone-3-carboxamide	NO	1.31	0.70	1.27E-02	C7H8N2O2	level1
Nordeoxycholic acid	NO	1.28	0.64	9.58E-03	C23H38O4	level1
Normetanephrine	NO	1.92	3.76	2.94E-03	C9H13NO3	level1
Ornithine	NO	1.76	1.50	9.26E-03	C5H12N2O2	level1
PC(35:4)_RT617	NO	1.25	0.76	4.91E-02	C43H78NO8P	level1
PC(36:3)_RT642	NO	1.34	0.79	9.58E-03	C44H82NO8P	level1
PC(37:6)_RT608	NO	1.42	0.65	1.79E-02	C45H78NO8P	level1
PC(40:6)_RT642	NO	1.48	0.78	4.91E-02	C48H84NO8P	level1
PC(40:7)_RT616	NO	1.49	0.79	2.81E-02	C48H82NO8P	level1
PC(42:8)_RT615	NO	1.52	0.75	9.26E-03	C50H84NO8P	level1
Pentadecanoic acid	NO	1.43	0.79	4.91E-02	C15H30O2	level1
Phenylpyruvic acid	NO	1.82	0.67	2.38E-05	C9H8O3	level1

Pristanic acid	NO	1.53	0.63	4.14E-03	C19H38O2	level1
Pyruvic acid	NO	1.85	1.77	1.44E-03	C3H4O3	level1
S1P(d18:0)	NO	1.17	1.35	1.44E-03	C18H40NO5P	level1
S1P(d18:1)	NO	1.45	1.36	1.50E-04	C18H38NO5P	level1
S1P(d18:2)_RT463	NO	1.74	1.41	1.98E-04	C18H36NO5P	level1
Stearamide	NO	1.42	0.04	3.77E-06	C18H37NO	level1
Traumatic acid	NO	1.12	0.66	4.91E-02	C12H20O4	level1
Tyrosine	NO	1.75	0.79	2.94E-03	C9H11NO3	level1
Tyrosine O-sulfate	NO	1.55	0.79	9.26E-03	C9H11NO6S	level1
Undecanoic acid	NO	1.64	0.78	1.44E-03	C11H22O2	level1
2-Hydroxyhexanedioic acid	NO	2.47	0.78	3.33E-07	C6H10O5	level2
Arabinono-1,4-lactone	NO	1.94	0.60	1.50E-04	C5H8O5	level2
Camphor	NO	1.69	1.49	2.60E-02	C10H16O	level2
Car(13:0)_RT433	NO	1.72	0.48	1.50E-04	C20H39NO4	level2
Car(14:2)_RT416	NO	1.75	0.46	1.40E-04	C21H37NO4	level2
Car(15:0)_RT486	NO	1.90	2.32	1.27E-02	C22H43NO4	level2
Car(15:1-O)_RT438	NO	2.16	0.57	1.44E-03	C22H41NO5	level2
Car(16:1-O2)_RT414	NO	2.11	0.53	4.09E-05	C23H41NO6	level2
Car(18:2)_RT502	NO	1.65	1.52	1.44E-03	C25H45NO4	level2
Car(5:1)_RT214	NO	1.55	0.69	1.16E-02	C12H21NO4	level2
Car(7:0)_RT288	NO	2.15	0.56	4.74E-07	C14H27NO4	level2
Car(9:1)_RT320	NO	1.39	0.72	9.26E-03	C16H29NO4	level2
Cer(d18:1/16:0)	NO	1.59	0.78	9.26E-03	C34H67NO3	level2
Lactaldehyde	NO	2.15	0.72	6.74E-06	C3H6O2	level2
PC(39:6)_RT628	NO	1.40	0.63	2.60E-02	C47H82NO8P	level2
(R)-10-Hydroxystearate	NO	1.74	0.68	1.44E-03	C18H36O3	level3

(R)-4'-Phosphopantothenoyl-L-cysteine	NO	1.49	0.78	2.81E-02	C12H23N2O9PS	level3
16-Oxopalmitate	NO	1.14	0.68	2.60E-02	C16H30O3	level3
16-Oxopalmitate	NO	1.54	1.56	1.44E-03	C16H30O3	level3
16-Oxopalmitate	NO	1.58	0.61	8.11E-03	C16H30O3	level3
18-Hydroxyoleate	NO	1.75	1.68	2.81E-02	C18H34O3	level3
18-Hydroxyoleate	NO	1.87	0.54	1.50E-04	C18H34O3	level3
18-Oxooleate	NO	1.82	0.58	1.98E-04	C18H32O3	level3
1-Aminocyclopropane-1-carboxylate	NO	1.26	0.68	4.91E-02	C4H7NO2	level3
2-Hydroxyhepta-2,4-dienedioate	NO	2.00	1.26	1.40E-04	C7H8O5	level3
2-Oxohept-3-enedioate	NO	2.00	1.26	1.40E-04	C7H8O5	level3
3-(4-Hydroxyphenyl)pyruvate	NO	1.72	0.45	1.98E-04	C9H8O4	level3
3alpha,7alpha-Dihydroxy-5beta-cholestanate	NO	2.27	1.87	2.44E-07	C27H46O4	level3
3beta,7alpha-Dihydroxy-5-cholestenoate	NO	1.92	1.93	8.08E-08	C27H44O4	level3
3-Chloro-D-alanine	NO	1.52	0.69	9.26E-03	C3H6ClNO2	level3
3-Chloro-L-alanine	NO	1.52	0.69	9.26E-03	C3H6ClNO2	level3
3-Dehydroshikimate	NO	2.00	1.26	1.40E-04	C7H8O5	level3
3-D-Glucuronosyl-N2,6-disulfo-beta-D-glucosamine	NO	1.20	0.75	4.58E-02	C12H21NO17S2	level3
4-(L-Alanin-3-yl)-2-hydroxy-cis,cis-muconate 6-semialdehyde	NO	2.28	1.42	1.83E-05	C9H11NO6	level3
4-(L-Alanin-3-yl)-2-hydroxy-cis,cis-muconate 6-semialdehyde	NO	2.57	1.50	2.44E-07	C9H11NO6	level3
4,5-seco-Dopa	NO	2.28	1.42	1.83E-05	C9H11NO6	level3
4,5-seco-Dopa	NO	2.57	1.50	2.44E-07	C9H11NO6	level3
4-Hydroxy-2-quinolone	NO	1.42	0.69	2.94E-03	C9H7NO2	level3
4-Imidazolone-5-propanoate	NO	1.55	0.23	1.83E-05	C6H8N2O3	level3

5-(L-Alanin-3-yl)-2-hydroxy-cis,cis-muconate 6-semialdehyde	NO	2.28	1.42	1.83E-05	C9H11NO6	level3
5-(L-Alanin-3-yl)-2-hydroxy-cis,cis-muconate 6-semialdehyde	NO	2.57	1.50	2.44E-07	C9H11NO6	level3
5'-S-Methyl-5'-thioinosine	NO	1.83	0.57	3.80E-04	C11H14N4O4S	level3
9,10-Dihydroxystearate	NO	1.75	1.68	2.81E-02	C18H36O4	level3
Acetyl-maltose	NO	1.63	0.77	9.26E-03	C14H24O12	level3
Aspirin	NO	1.72	0.45	1.98E-04	C9H8O4	level3
Betalamic acid	NO	2.28	1.42	1.83E-05	C9H9NO5	level3
CDP-choline	NO	1.59	1.50	4.58E-02	C14H26N4O11P2	level3
cis-9,10-Epoxystearic acid	NO	1.75	1.68	2.81E-02	C18H34O3	level3
cis-9,10-Epoxystearic acid	NO	1.87	0.54	1.50E-04	C18H34O3	level3
D-Galacturonate	NO	1.41	0.77	2.81E-02	C6H10O7	level3
D-Glucose	NO	2.17	0.77	3.77E-06	C6H12O6	level3
D-Glucose/alpha-D-Galactose	NO	2.17	0.77	3.77E-06	C6H12O6	level3
D-Glucose/beta-D-Glucose	NO	2.17	0.77	3.77E-06	C6H12O6	level3
D-Glucose/D-Fructose	NO	2.17	0.77	3.77E-06	C6H12O6	level3
D-Glucose/D-Galactose	NO	2.17	0.77	3.77E-06	C6H12O6	level3
D-Octopine	NO	1.36	0.80	4.91E-02	C9H18N4O4	level3
Fumarate	NO	1.48	0.69	4.91E-02	C4H4O4	level3
Isoniazid alpha-ketoglutaric acid	NO	2.69	2.49	2.06E-07	C11H11N3O5	level3
Maleic acid	NO	1.48	0.69	4.91E-02	C4H4O4	level3
Methyl cinnamate	NO	2.23	3.29	1.26E-07	C10H10O2	level3
N-Acetyl-L-glutamate	NO	2.28	1.42	1.83E-05	C7H11NO5	level3
Nicotinate D-ribonucleoside	NO	2.15	2.15	4.09E-05	C11H14NO6	level3
N-Succinyl-LL-2,6-diaminoheptanedioate	NO	1.50	0.61	8.11E-03	C11H18N2O7	level3

Porphobilinogen	NO	2.02	0.71	1.50E-04	$C_{10}H_{14}N_2O_4$	level3
Sphingosyl-phosphocholine	NO	2.35	2.08	8.08E-08	$C_{23}H_{50}N_2O_5P$	level3

VIP: variable importance on projection.

FDR P.value: P.value of FDR-adjusted Wilcoxon–Mann–Whitney test

FC: fold change of disorder / HC
