# Supplementary material for: Identifying plasma metabolic characteristics of major depressive disorder, bipolar disorder, and schizophrenia in adolescents
Source: Transl Psychiatry. 2024 Mar 26;14:163. doi: 10.1038/s41398-024-02886-z (PMC10966062; doi:10.1038/s41398-024-02886-z)
Supplement: Supplementary file 10 — Supplementary Table 4 [file 41398_2024_2886_MOESM10_ESM.pdf]

Supplementary Table 4A: Detailed information of differentially expressed acetylcarnitine metabolites from the disorder-HC comparisons (MDD-HC).

Name	Biomarker	Vip	FC	FDR P.value	Formula	Confidence_level
Car(11:1)_RT383	YES	2.36	0.49	8.80E-05	C18H33NO4	level1
Car(9:0)_RT346	YES	2.09	0.46	8.80E-05	C16H31NO4	level1
Car(3:0)_RT63	NO	1.63	0.77	1.20E-02	C10H19NO4	level1
Car(12:0)_RT430	NO	1.92	0.48	1.78E-04	C19H37NO4	level1
Car(14:0)_RT467	NO	1.82	0.66	6.37E-03	C21H41NO4	level1
Car(10:1)_RT357	NO	1.74	0.59	2.89E-03	C17H31NO4	level1
Car(12:1)_RT402	NO	1.68	0.55	1.20E-02	C19H35NO4	level1
Car(14:1)_RT447	NO	1.61	0.55	1.20E-02	C21H39NO4	level1
Car(14:1)_RT438	NO	1.61	0.55	1.20E-02	C21H39NO4	level1
Car(12:2)_RT382	NO	1.93	0.61	3.19E-03	C19H33NO4	level1
Car(16:3)_RT442	NO	2.04	0.59	2.89E-03	C23H39NO4	level1
Car(16:4)_RT421	NO	2.14	0.49	6.95E-04	C23H37NO4	level1
Car(14:1-O)_RT416	NO	1.90	0.49	5.47E-03	C21H39NO5	level1
Car(16:1-O)_RT453	NO	1.80	0.61	1.20E-02	C23H43NO5	level1
Car(14:2-O)_RT389	NO	1.95	0.53	2.89E-03	C21H37NO5	level1
Car(16:2-O)_RT395	NO	1.64	0.67	1.20E-02	C23H41NO5	level1
Car(6:0)_RT250	NO	1.82	0.66	1.20E-02	C13H25NO4	level1
Car(6:0)_RT263	NO	1.82	0.66	1.20E-02	C13H25NO4	level1
Car(8:0)_RT316	NO	1.92	0.49	5.14E-04	C15H29NO4	level1
Car(10:0)_RT370	NO	2.02	0.45	1.78E-04	C17H33NO4	level1
Car(11:0)_RT390	NO	1.84	0.36	8.77E-06	C18H35NO4	level1
Car(15:1-O)_RT438	NO	2.53	0.56	3.19E-03	C22H41NO5	level2
Car(11:1-O2)_RT289	NO	2.08	0.57	2.89E-03	C18H31NO6	level2
Car(7:0)_RT288	NO	1.94	0.67	6.95E-04	C14H27NO4	level2

Car(13:0)_RT433	NO	1.52	0.62	6.37E-03	C20H39NO4	level2
Car(14:2)_RT416	NO	1.62	0.61	1.20E-02	C21H37NO4	level2
Car(12:1-O2)_RT315	NO	1.84	0.58	1.20E-02	C19H33NO6	level2
Car(16:1-O2)_RT414	NO	1.92	0.64	3.19E-03	C23H41NO6	level2

VIP: variable importance on projection.

FDR P.value: P.value of FDR-adjusted Wilcoxon–Mann–Whitney test

FC: fold change of disorder / HC

Supplementary Table 4B:Detailed information of differentially expressed acetylcarnitine metabolites from the disorder-HC comparisons (BD-HC).

Name	Biomarker	Vip	FC	FDR P.value	Formula	Confidence_level
Car(12:2)_RT382	YES	2.08	0.48	5.75E-05	C19H33NO4	level1
Car(3:0)_RT63	NO	1.59	0.75	1.58E-03	C10H19NO4	level1
Car(12:0)_RT430	NO	1.30	0.61	2.90E-03	C19H37NO4	level1
Car(10:1)_RT357	NO	1.23	0.65	2.90E-03	C17H31NO4	level1
Car(11:1)_RT383	NO	2.13	0.49	8.82E-06	C18H33NO4	level1
Car(12:1)_RT402	NO	1.27	0.60	1.83E-02	C19H35NO4	level1
Car(16:3)_RT442	NO	1.43	0.69	1.83E-02	C23H39NO4	level1
Car(12:1-O)_RT364	NO	1.53	0.59	2.90E-03	C19H35NO5	level1
Car(14:1-O)_RT416	NO	1.71	0.48	1.19E-03	C21H39NO5	level1
Car(16:1-O)_RT453	NO	1.32	0.68	4.27E-02	C23H43NO5	level1
Car(14:2-O)_RT389	NO	1.79	0.50	3.21E-04	C21H37NO5	level1
Car(16:2-O)_RT395	NO	1.81	0.56	1.19E-03	C23H41NO5	level1
Car(9:0)_RT346	NO	1.47	0.55	6.50E-04	C16H31NO4	level1
Car(11:0)_RT390	NO	1.46	0.44	8.82E-06	C18H35NO4	level1
Car(15:1-O)_RT438	NO	2.56	0.51	3.21E-04	C22H41NO5	level2
Car(5:1)_RT214	NO	1.56	0.70	1.06E-02	C12H21NO4	level2
Car(9:1)_RT320	NO	1.40	0.72	2.90E-03	C16H29NO4	level2
Car(16:1-O2)_RT414	NO	1.90	0.57	1.34E-04	C23H41NO6	level2

VIP: variable importance on projection.

FDR P.value: P.value of FDR-adjusted Wilcoxon–Mann–Whitney test

FC: fold change of disorder / HC

Supplementary Table 4C: Detailed information of differentially expressed acetylcarnitine metabolites from the disorder-HC comparisons (SCZ-HC).

Name	Biomarker	Vip	FC	FDR P.value	Formula	Confidence_level
Car(11:1)_RT383	YES	2.07	0.42	2.44E-07	C18H33NO4	level1
Car(16:2-O)_RT395	YES	2.15	0.47	4.09E-05	C23H41NO5	level1
Car(9:0)_RT346	YES	2.42	0.25	8.08E-08	C16H31NO4	level1
Car(3:0)_RT63	NO	1.61	0.75	9.26E-03	C10H19NO4	level1
Car(12:0)_RT430	NO	2.02	0.33	2.06E-07	C19H37NO4	level1
Car(14:0)_RT467	NO	1.84	0.60	1.98E-04	C21H41NO4	level1
Car(10:1)_RT357	NO	1.82	0.48	7.63E-06	C17H31NO4	level1
Car(12:1)_RT402	NO	1.68	0.44	3.80E-04	C19H35NO4	level1
Car(14:1)_RT447	NO	1.54	0.47	1.44E-03	C21H39NO4	level1
Car(14:1)_RT438	NO	1.54	0.47	1.44E-03	C21H39NO4	level1
Car(10:2)_RT333	NO	1.32	0.61	9.58E-03	C17H29NO4	level1
Car(4:0)_RT100	NO	1.49	0.71	9.26E-03	C11H21NO4	level1
Car(10:2)_RT347	NO	1.32	0.61	9.58E-03	C17H29NO4	level1
Car(12:2)_RT382	NO	2.25	0.41	2.44E-07	C19H33NO4	level1
Car(16:2)_RT456	NO	1.41	0.57	9.26E-03	C23H41NO4	level1
Car(16:3)_RT442	NO	1.94	0.53	9.02E-04	C23H39NO4	level1
Car(18:1)	NO	1.49	1.34	2.60E-02	C25H47NO4	level1
Car(16:4)_RT421	NO	1.87	0.46	1.98E-04	C23H37NO4	level1
Car(12:1-O)_RT364	NO	1.93	0.45	2.38E-05	C19H35NO5	level1
Car(14:1-O)_RT416	NO	1.92	0.36	7.63E-06	C21H39NO5	level1
Car(16:1-O)_RT453	NO	1.84	0.52	1.44E-03	C23H43NO5	level1
Car(12:2-O)_RT340	NO	1.54	0.51	9.26E-03	C19H33NO5	level1
Car(14:2-O)_RT389	NO	2.18	0.35	4.74E-07	C21H37NO5	level1
Car(6:0)_RT250	NO	1.52	0.60	1.40E-04	C13H25NO4	level1

Car(6:0)_RT263	NO	1.52	0.60	1.40E-04	C13H25NO4	level1
Car(8:0)_RT316	NO	1.88	0.38	6.74E-06	C15H29NO4	level1
Car(10:0)_RT370	NO	1.95	0.35	2.44E-07	C17H33NO4	level1
Car(11:0)_RT390	NO	1.95	0.19	1.71E-09	C18H35NO4	level1
Car(7:0)_RT288	NO	2.15	0.56	4.74E-07	C14H27NO4	level2
Car(13:0)_RT433	NO	1.72	0.48	1.50E-04	C20H39NO4	level2
Car(5:1)_RT214	NO	1.55	0.69	1.16E-02	C12H21NO4	level2
Car(9:1)_RT320	NO	1.39	0.72	9.26E-03	C16H29NO4	level2
Car(14:2)_RT416	NO	1.75	0.46	1.40E-04	C21H37NO4	level2
Car(18:2)_RT502	NO	1.65	1.52	1.44E-03	C25H45NO4	level2
Car(15:1-O)_RT438	NO	2.16	0.57	1.44E-03	C22H41NO5	level2
Car(16:1-O2)_RT414	NO	2.11	0.53	4.09E-05	C23H41NO6	level2

VIP: variable importance on projection.

FDR P.value: P.value of FDR-adjusted Wilcoxon–Mann–Whitney test

FC: fold change of disorder / HC
