# Supplementary material for: Identifying plasma metabolic characteristics of major depressive disorder, bipolar disorder, and schizophrenia in adolescents
Source: Transl Psychiatry. 2024 Mar 26;14:163. doi: 10.1038/s41398-024-02886-z (PMC10966062; doi:10.1038/s41398-024-02886-z)
Supplement: Supplementary file 11 — Supplementary Table 5 [file 41398_2024_2886_MOESM11_ESM.pdf]

Supplementary Table 5A: Detailed information of the disorder-disorder differentially expressed metabolites selected from respective union-set of differentially expressed metabolites from the disorder-HC comparisons.(MDD-SCZ from MDD-HC & SCZ-HC)

Name	Biomarker	FDR P.value	FC	Formula	Confidence_level
Pyroglutamic acid	YES	9.62E-07	0.73	C5H7NO3	level1
Car(11:0)_RT390	YES	1.07E-04	1.86	C18H35NO4	level1
Xanthine	YES	1.07E-04	0.73	C5H4N4O2	level1
Phenylpyruvic acid	YES	7.89E-04	1.28	C9H8O3	level1
Car(12:0)_RT430	YES	9.11E-03	1.46	C19H37NO4	level1
Car(12:1-O)_RT364	YES	1.24E-02	1.81	C19H35NO5	level1
Dodecanoic acid	YES	1.78E-02	1.56	C12H24O2	level1
Car(16:2-O)_RT395	YES	4.21E-02	1.42	C23H41NO5	level1
4-Methylsalicylic acid	NO	3.38E-05	2.36	C8H8O3	level1
Car(18:1)	NO	1.07E-04	0.68	C25H47NO4	level1
Dihydroxyacetone	NO	1.07E-04	0.69	C3H6O3	level1
Glyceraldehyde	NO	1.07E-04	0.69	C3H6O3	level1
Xanthurenic acid	NO	1.07E-04	1.70	C10H7NO4	level1
Glycerophosphocholine	NO	7.89E-04	0.82	C8H20NO6P	level1
Lactic acid	NO	7.89E-04	0.71	C3H6O3	level1
Ornithine	NO	8.96E-04	0.63	C5H12N2O2	level1
Car(9:0)_RT346	NO	2.58E-03	1.84	C16H31NO4	level1
S1P(d18:1)	NO	2.58E-03	0.81	C18H38NO5P	level1
3-methylcytidine	NO	9.11E-03	1.33	C10H15N3O5	level1
Methionine	NO	9.11E-03	0.87	C5H11NO2S	level1
PC(36:3)_RT642	NO	9.11E-03	1.25	C44H82NO8P	level1
Car(12:2)_RT382	NO	1.24E-02	1.49	C19H33NO4	level1
Lysine	NO	1.24E-02	0.74	C6H14N2O2	level1

S1P(d18:2)_RT463	NO	1.24E-02	0.81	C18H36NO5P	level1
Traumatic acid	NO	1.24E-02	1.48	C12H20O4	level1
Car(12:2-O)_RT340	NO	1.35E-02	1.87	C19H33NO5	level1
Car(14:1-O)_RT416	NO	1.35E-02	1.37	C21H39NO5	level1
Car(14:2-O)_RT389	NO	1.35E-02	1.51	C21H37NO5	level1
4-Vinylphenol	NO	1.78E-02	1.58	C8H8O	level1
Acetoin	NO	1.78E-02	4.60	C4H8O2	level1
Arachidonic acid (AA)	NO	1.78E-02	1.27	C20H32O2	level1
Car(10:0)_RT370	NO	1.78E-02	1.30	C17H33NO4	level1
Car(11:1)_RT383	NO	1.78E-02	1.16	C18H33NO4	level1
Car(8:0)_RT316	NO	1.78E-02	1.29	C15H29NO4	level1
Chenodeoxycholic acid	NO	1.78E-02	0.59	C24H40O4	level1
Deoxycholic acid	NO	1.78E-02	0.59	C24H40O4	level1
gamma-Glutamyllysine	NO	1.78E-02	0.71	C11H21N3O5	level1
PC(40:6)_RT642	NO	1.78E-02	1.21	C48H84NO8P	level1
PC(42:8)_RT615	NO	1.78E-02	1.22	C50H84NO8P	level1
Stearamide	NO	1.78E-02	19.92	C18H37NO	level1
Adenosine 3',5'-cyclic phosphate (cAMP)	NO	3.48E-02	1.14	C10H12N5O6P	level1
Arginine	NO	3.48E-02	1.22	C6H14N4O2	level1
Dimethylglycine	NO	3.48E-02	0.78	C4H9NO2	level1
S1P(d18:0)	NO	4.21E-02	0.86	C18H40NO5P	level1
Capric acid	NO	6.61E-02	1.14	C10H20O2	level1
Histamine	NO	6.61E-02	1.37	C5H9N3	level1
Indole-3-pyruvic acid	NO	6.61E-02	1.15	C11H9NO3	level1
Histidinol	NO	7.71E-02	0.85	C6H11N3O	level1

Kynurenic acid	NO	7.71E-02	1.20	C10H7NO3	level1
PC(40:7)_RT616	NO	7.71E-02	1.12	C48H82NO8P	level1
Pristanic acid	NO	7.71E-02	1.16	C19H38O2	level1
Car(6:0)_RT250	NO	9.01E-02	1.09	C13H25NO4	level1
Car(6:0)_RT263	NO	9.01E-02	1.09	C13H25NO4	level1
Aspartic acid	NO	1.01E-01	1.66	C4H7NO4	level1
Car(10:1)_RT357	NO	1.01E-01	1.23	C17H31NO4	level1
Car(12:1)_RT402	NO	1.01E-01	1.24	C19H35NO4	level1
Car(16:2)_RT456	NO	1.01E-01	1.25	C23H41NO4	level1
Docosahexaenoic acid (DHA)	NO	1.01E-01	1.17	C22H32O2	level1
Indolelactic acid	NO	1.01E-01	1.11	C11H11NO3	level1
PC(37:6)_RT608	NO	1.01E-01	1.18	C45H78NO8P	level1
Acetylcarnitine (Car(2:0))	NO	1.55E-01	1.11	C9H17NO4	level1
Car(10:2)_RT333	NO	1.55E-01	1.18	C17H29NO4	level1
Car(10:2)_RT347	NO	1.55E-01	1.18	C17H29NO4	level1
Car(14:1)_RT438	NO	1.55E-01	1.18	C21H39NO4	level1
Car(14:1)_RT447	NO	1.55E-01	1.18	C21H39NO4	level1
Car(16:1-O)_RT453	NO	1.55E-01	1.19	C23H43NO5	level1
Epinephrine	NO	1.55E-01	0.84	C9H13NO3	level1
Glutamic acid	NO	1.55E-01	0.89	C5H9NO4	level1
Isovalerylcarnitine	NO	1.55E-01	1.14	C12H23NO4	level1
Normetanephrine	NO	1.55E-01	0.84	C9H13NO3	level1
PC(35:4)_RT617	NO	1.55E-01	1.08	C43H78NO8P	level1
Biliverdin	NO	3.18E-01	0.87	C33H34N4O6	level1
Pyruvic acid	NO	3.18E-01	0.81	C3H4O3	level1
Tyrosine	NO	3.18E-01	1.05	C9H11NO3	level1

Car(16:4)_RT421	NO	3.31E-01	1.08	C23H37NO4	level1
Eicosapentaenoic acid	NO	3.31E-01	1.20	C20H30O2	level1
Nordeoxycholic acid	NO	4.09E-01	1.02	C23H38O4	level1
Salicylic acid	NO	4.09E-01	0.56	C7H6O3	level1
Undecanoic acid	NO	4.09E-01	1.04	C11H22O2	level1
Myristic acid	NO	4.98E-01	1.09	C14H28O2	level1
Car(14:0)_RT467	NO	5.42E-01	1.11	C21H41NO4	level1
Car(16:3)_RT442	NO	5.42E-01	1.12	C23H39NO4	level1
Dehydrolithocholic acid	NO	5.42E-01	1.04	C24H38O3	level1
Heptadecanoic acid	NO	5.42E-01	1.07	C17H34O2	level1
Butyrylcarnitine	NO	1	1.11	C11H21NO4	level1
Car(4:0)_RT100	NO	1	1.11	C11H21NO4	level1
Pentadecanoic acid	NO	1	1.04	C15H30O2	level1
2-Aminoadipic acid	NO	1	1.06	C6H11NO4	level1
5-Aminolevulinic acid	NO	1	0.98	C5H9NO3	level1
Cortisone/Aldosterone	NO	1	1.00	C21H28O5	level1
Car(3:0)_RT63	NO	1	1.03	C10H19NO4	level1
cis-8,11,14-Eicosatrienoic acid	NO	1	1.03	C20H34O2	level1
Cortisone/Aldosterone	NO	1	1.00	C21H28O5	level1
Kynurenine	NO	1	0.99	C10H12N2O3	level1
N1-Methyl-4-pyridone-3-carboxamide	NO	1	1.01	C7H8N2O2	level1
Tyrosine O-sulfate	NO	1	1.01	C9H11NO6S	level1
2-Hydroxyhexanedioic acid	NO	3.34E-04	1.26	C6H10O5	level2
Car(18:2)_RT502	NO	8.96E-04	0.72	C25H45NO4	level2
Arabinono-1,4-lactone	NO	2.58E-03	1.51	C5H8O5	level2
Car(11:1-O2)_RT289	NO	9.11E-03	0.58	C18H31NO6	level2

Cer(d18:1/16:0)	NO	1.24E-02	1.28	C34H67NO3	level2
4-Hydroxyglutamic acid	NO	1.78E-02	1.26	C5H9NO5	level2
Car(12:1-O2)_RT315	NO	3.48E-02	0.60	C19H33NO6	level2
Car(14:2)_RT416	NO	6.61E-02	1.32	C21H37NO4	level2
Car(7:0)_RT288	NO	6.61E-02	1.19	C14H27NO4	level2
Lactaldehyde	NO	6.61E-02	1.12	C3H6O2	level2
Car(13:0)_RT433	NO	7.71E-02	1.29	C20H39NO4	level2
Car(16:1-O2)_RT414	NO	1.01E-01	1.21	C23H41NO6	level2
Cysteine	NO	1.01E-01	0.81	C3H7NO2S	level2
Camphor	NO	1.55E-01	0.85	C10H16O	level2
Car(5:1)_RT214	NO	1.55E-01	1.12	C12H21NO4	level2
Car(15:0)_RT486	NO	3.31E-01	0.72	C22H43NO4	level2
PC(39:6)_RT628	NO	3.31E-01	1.21	C47H82NO8P	level2
Car(9:1)_RT320	NO	5.42E-01	1.09	C16H29NO4	level2
Car(15:1-O)_RT438	NO	1	0.99	C22H41NO5	level2
Sphingosyl-phosphocholine	NO	2.23E-07	0.52	C23H50N2O5P	level3
Methyl cinnamate	NO	9.06E-07	0.40	C10H10O2	level3
3beta,7alpha-Dihydroxy-5-cholestenoate	NO	9.06E-07	0.54	C27H44O4	level3
3alpha,7alpha-Dihydroxy-5beta-cholestanate	NO	3.38E-05	0.58	C27H46O4	level3
4-(L-Alanin-3-yl)-2-hydroxy-cis,cis-muconate 6-semialdehyde	NO	3.38E-05	0.76	C9H11NO6	level3
4,5-seco-Dopa	NO	3.38E-05	0.76	C9H11NO6	level3
5-(L-Alanin-3-yl)-2-hydroxy-cis,cis-muconate 6-semialdehyde	NO	3.38E-05	0.76	C9H11NO6	level3
alpha-D-Galactose	NO	1.07E-04	1.23	C6H12O6	level3

beta-D-Glucose	NO	1.07E-04	1.23	C6H12O6	level3
D-Fructose	NO	1.07E-04	1.23	C6H12O6	level3
D-Galactose	NO	1.07E-04	1.23	C6H12O6	level3
D-Glucose	NO	1.07E-04	1.23	C6H12O6	level3
Isoniazid alpha-ketoglutaric acid	NO	1.07E-04	0.62	C11H11N3O5	level3
2-Hydroxyhepta-2,4-dienedioate	NO	2.83E-04	0.84	C7H8O5	level3
2-Oxohept-3-enedioate	NO	2.83E-04	0.84	C7H8O5	level3
3-Dehydroshikimate	NO	2.83E-04	0.84	C7H8O5	level3
Nicotinate D-ribonucleoside	NO	2.83E-04	0.57	C11H14NO6	level3
beta-Citryl-L-glutamate	NO	8.96E-04	0.72	C11H15NO10	level3
Miraxanthin-V	NO	8.96E-04	0.49	C17H18N2O6	level3
4-(L-Alanin-3-yl)-2-hydroxy-cis,cis-muconate 6-semialdehyde	NO	9.11E-04	0.82	C9H11NO6	level3
4,5-seco-Dopa	NO	9.11E-04	0.82	C9H11NO6	level3
5-(L-Alanin-3-yl)-2-hydroxy-cis,cis-muconate 6-semialdehyde	NO	9.11E-04	0.82	C9H11NO6	level3
Betalamic acid	NO	9.11E-04	0.82	C9H9NO5	level3
N-Acetyl-L-glutamate	NO	9.11E-04	0.82	C7H11NO5	level3
3-Indoleacrylate	NO	9.11E-03	0.86	C11H9NO2	level3
4-Imidazolone-5-propanoate	NO	1.78E-02	7.83	C6H8N2O3	level3
(R)-4'-Phosphopantothenoyl-L-cysteine	NO	1.78E-02	1.18	C12H23N2O9PS	level3
Phosphocreatine	NO	1.78E-02	0.78	C4H10N3O5P	level3
18-Hydroxyoleate	NO	3.48E-02	1.19	C18H34O3	level3
18-Oxooleate	NO	3.48E-02	1.18	C18H32O3	level3
3-(4-Hydroxyphenyl)pyruvate	NO	3.48E-02	1.48	C9H8O4	level3
Aspirin	NO	3.48E-02	1.48	C9H8O4	level3

cis-9,10-Epoxystearic acid	NO	3.48E-02	1.19	C18H34O3	level3
L-Cystine	NO	6.61E-02	0.79	C6H12N2O4S2	level3
N-Succinyl-LL-2,6-diaminoheptanedioate	NO	6.61E-02	2.01	C11H18N2O7	level3
(3Z)-Phytochromobilin	NO	7.71E-02	0.75	C33H36N4O6	level3
15,16-Dihydrobiliverdin	NO	7.71E-02	0.75	C33H36N4O6	level3
16-Oxopalmitate	NO	7.71E-02	1.20	C16H30O3	level3
3-D-Glucuronosyl-N2,6-disulfo-beta-D-glucosamine	NO	1.01E-01	1.28	C12H21NO17S2	level3
D-Galacturonate	NO	1.01E-01	1.16	C6H10O7	level3
1-Aminocyclopropane-1-carboxylate	NO	1.55E-01	1.28	C4H7NO2	level3
3-Chloro-D-alanine	NO	1.55E-01	1.17	C3H6ClNO2	level3
3-Chloro-L-alanine	NO	1.55E-01	1.17	C3H6ClNO2	level3
3-Indoleacrylate	NO	1.55E-01	0.93	C11H9NO2	level3
4-Hydroxy-2-quinolone	NO	1.55E-01	1.14	C9H7NO2	level3
Fumarate	NO	3.18E-01	1.35	C4H4O4	level3
Maleic acid	NO	3.18E-01	1.35	C4H4O4	level3
(R)-10-Hydroxystearate	NO	3.31E-01	1.05	C18H36O3	level3
18-Hydroxyoleate	NO	3.31E-01	0.85	C18H34O3	level3
9,10-Dihydroxystearate	NO	3.31E-01	0.85	C18H36O4	level3
cis-9,10-Epoxystearic acid	NO	3.31E-01	0.85	C18H34O3	level3
16-Oxopalmitate	NO	4.09E-01	1.12	C16H30O3	level3
CDP-choline	NO	4.09E-01	0.91	C14H26N4O11P2	level3
Formylisoglutamine	NO	4.09E-01	1.10	C6H10N2O4	level3
5'-S-Methyl-5'-thioinosine	NO	1	1.05	C11H14N4O4S	level3
Porphobilinogen	NO	1	1.04	C10H14N2O4	level3

16-Oxopalmitate	NO	1	1.06	C16H30O3	level3
Acetyl-maltose	NO	1	1.02	C14H24O12	level3
D-Octopine	NO	1	1.19	C9H18N4O4	level3

VIP: variable importance on projection;

FC: fold change of MDD / SCZ;

FDR P.value: P.value of FDR-adjusted Wilcoxon–Mann–Whitney test.

Supplementary Table 5B: Detailed information of the disorder-disorder differentially expressed metabolites selected from respective union-set of differentially expressed metabolites from the disorder-HC comparisons.(BD-SCZ from BD-HC & SCZ-HC)

Name	Biomarker	FDR P.value	FC	Formula	Confidence_level
Car(9:0)_RT346	YES	9.89E-03	2.17	C16H31NO4	level1
Dodecanoic acid	YES	9.89E-03	1.76	C12H24O2	level1
Xanthurenic acid	YES	9.89E-03	1.65	C10H7NO4	level1
2-Aminooctanoic acid	YES	4.74E-02	0.72	C8H17NO2	level1
Ornithine	NO	3.23E-03	0.62	C5H12N2O2	level1
Pyroglutamic acid	NO	3.23E-03	0.86	C5H7NO3	level1
Glycerophosphocholine	NO	9.89E-03	0.85	C8H20NO6P	level1
Lysine	NO	9.89E-03	0.71	C6H14N2O2	level1
Methionine	NO	9.89E-03	0.84	C5H11NO2S	level1
PC(36:3)_RT642	NO	9.89E-03	1.32	C44H82NO8P	level1
S1P(d18:1)	NO	9.89E-03	0.82	C18H38NO5P	level1
S1P(d18:2)_RT463	NO	9.89E-03	0.81	C18H36NO5P	level1
Undecanoic acid	NO	9.89E-03	1.22	C11H22O2	level1
Dihydroxyacetone	NO	1.11E-02	0.87	C3H6O3	level1
Glyceraldehyde	NO	1.11E-02	0.87	C3H6O3	level1
Lactic acid	NO	1.11E-02	0.84	C3H6O3	level1
PC(40:6)_RT642	NO	1.11E-02	1.26	C48H84NO8P	level1
Car(18:1)	NO	1.20E-02	0.82	C25H47NO4	level1
PC(37:6)_RT608	NO	1.20E-02	1.53	C45H78NO8P	level1
PC(40:7)_RT616	NO	1.20E-02	1.24	C48H82NO8P	level1
Car(11:0)_RT390	NO	1.70E-02	2.29	C18H35NO4	level1
gamma-Glutamyllysine	NO	1.70E-02	0.67	C11H21N3O5	level1
5-Aminopentanoic acid	NO	1.78E-02	0.89	C5H11NO2	level1

Erucamide	NO	1.78E-02	2.15	C22H43NO	level1
gamma-Glutamylvaline	NO	1.78E-02	0.84	C10H18N2O5	level1
Malic acid	NO	1.78E-02	0.79	C4H6O5	level1
Pristanic acid	NO	1.78E-02	1.37	C19H38O2	level1
gamma-Glutamylalanine	NO	2.56E-02	0.72	C8H14N2O5	level1
Histidinol	NO	2.56E-02	0.79	C6H11N3O	level1
S1P(d18:0)	NO	2.56E-02	0.85	C18H40NO5P	level1
Arachidonic acid (AA)	NO	3.41E-02	1.27	C20H32O2	level1
Heptadecanoic acid	NO	3.41E-02	1.36	C17H34O2	level1
PC(35:4)_RT617	NO	3.41E-02	1.33	C43H78NO8P	level1
Acetoin	NO	4.74E-02	4.82	C4H8O2	level1
Car(12:0)_RT430	NO	4.74E-02	1.88	C19H37NO4	level1
Docosahexaenoic acid (DHA)	NO	4.74E-02	1.32	C22H32O2	level1
Eicosapentaenoic acid	NO	4.74E-02	1.41	C20H30O2	level1
PC(42:8)_RT615	NO	4.74E-02	1.17	C50H84NO8P	level1
Salicylic acid	NO	4.74E-02	0.56	C7H6O3	level1
4-Methylsalicylic acid	NO	7.72E-02	1.77	C8H8O3	level1
Car(11:1)_RT383	NO	9.71E-02	1.15	C18H33NO4	level1
Car(6:0)_RT250	NO	9.71E-02	1.58	C13H25NO4	level1
Car(6:0)_RT263	NO	9.71E-02	1.58	C13H25NO4	level1
Pentadecanoic acid	NO	9.71E-02	1.19	C15H30O2	level1
Dimethylglycine	NO	1.03E-01	0.83	C4H9NO2	level1
4-Vinylphenol	NO	1.44E-01	1.32	C8H8O	level1
Capric acid	NO	1.44E-01	1.40	C10H20O2	level1
Car(12:1-O)_RT364	NO	1.44E-01	1.32	C19H35NO5	level1
Car(14:0)_RT467	NO	1.44E-01	1.41	C21H41NO4	level1

Car(14:1)_RT438	NO	1.44E-01	1.50	C21H39NO4	level1
Car(14:1)_RT447	NO	1.44E-01	1.50	C21H39NO4	level1
Car(14:1-O)_RT416	NO	1.44E-01	1.33	C21H39NO5	level1
Car(14:2-O)_RT389	NO	1.44E-01	1.43	C21H37NO5	level1
Car(8:0)_RT316	NO	1.44E-01	1.90	C15H29NO4	level1
cis-8,11,14-Eicosatrienoic acid	NO	1.44E-01	1.18	C20H34O2	level1
Epinephrine	NO	1.44E-01	0.68	C9H13NO3	level1
Glucuronic acid	NO	1.44E-01	1.19	C6H10O7	level1
Homoarginine	NO	1.44E-01	0.87	C7H16N4O2	level1
Normetanephrine	NO	1.44E-01	0.68	C9H13NO3	level1
Phenylpyruvic acid	NO	1.44E-01	1.11	C9H8O3	level1
Pyruvic acid	NO	1.44E-01	0.95	C3H4O3	level1
Stearamide	NO	1.44E-01	9.98	C18H37NO	level1
Car(10:2)_RT333	NO	1.55E-01	1.26	C17H29NO4	level1
Car(10:2)_RT347	NO	1.55E-01	1.26	C17H29NO4	level1
Car(10:0)_RT370	NO	1.66E-01	1.86	C17H33NO4	level1
Car(16:1-O)_RT453	NO	1.66E-01	1.32	C23H43NO5	level1
Car(16:4)_RT421	NO	1.66E-01	1.54	C23H37NO4	level1
Histamine	NO	1.66E-01	1.34	C5H9N3	level1
Myristic acid	NO	1.66E-01	1.23	C14H28O2	level1
Adenosine 3',5'-cyclic phosphate (cAMP)	NO	2.11E-01	1.05	C10H12N5O6P	level1
Car(12:1)_RT402	NO	2.11E-01	1.35	C19H35NO4	level1
2-Ketocaproic acid	NO	3.07E-01	0.95	C6H10O3	level1
3-Methyl-2-oxovaleric acid	NO	3.07E-01	0.95	C6H10O3	level1
Car(16:2)_RT456	NO	3.07E-01	1.58	C23H41NO4	level1

Glutamic acid	NO	3.07E-01	0.92	C5H9NO4	level1
Ketoleucine	NO	3.07E-01	0.95	C6H10O3	level1
2-Hydroxyoctanoic acid	NO	6.22E-01	0.86	C8H16O3	level1
3alpha-Hydroxy-12-oxo-5beta-cholan-24-oic acid	NO	6.22E-01	0.84	C24H38O4	level1
3alpha-Hydroxy-6-oxo-5alpha-cholan-24-oic acid	NO	6.22E-01	0.84	C24H38O4	level1
3-methylcytidine	NO	6.22E-01	1.05	C10H15N3O5	level1
Acetylcarnitine (Car(2:0))	NO	6.22E-01	1.12	C9H17NO4	level1
Cortisone/Aldosterone	NO	6.22E-01	0.94	C21H28O5	level1
Allolithocholic acid	NO	6.22E-01	0.87	C24H40O3	level1
Apocholic acid	NO	6.22E-01	0.84	C24H38O4	level1
Arginine	NO	6.22E-01	1.06	C6H14N4O2	level1
Butyrylcarnitine	NO	6.22E-01	1.15	C11H21NO4	level1
Car(10:1)_RT357	NO	6.22E-01	1.35	C17H31NO4	level1
Car(12:2)_RT382	NO	6.22E-01	1.19	C19H33NO4	level1
Car(12:2-O)_RT340	NO	6.22E-01	1.27	C19H33NO5	level1
Car(16:3)_RT442	NO	6.22E-01	1.30	C23H39NO4	level1
Car(4:0)_RT100	NO	6.22E-01	1.15	C11H21NO4	level1
Cortisone/Aldosterone	NO	6.22E-01	0.94	C21H28O5	level1
Cysteic acid	NO	6.22E-01	1.10	C3H7NO5S	level1
Indole-3-pyruvic acid	NO	6.22E-01	1.08	C11H9NO3	level1
Isovalerylcarnitine	NO	6.22E-01	1.07	C12H23NO4	level1
Kynurenine	NO	6.22E-01	0.98	C10H12N2O3	level1
Lithocholic acid	NO	6.22E-01	0.87	C24H40O3	level1
Nordeoxycholic acid	NO	6.22E-01	0.81	C23H38O4	level1

Traumatic acid	NO	6.22E-01	1.11	C12H20O4	level1
Tyrosine	NO	6.22E-01	0.98	C9H11NO3	level1
Urocanic acid	NO	6.22E-01	0.86	C6H6N2O2	level1
Car(16:2-O)_RT395	NO	1	1.20	C23H41NO5	level1
2-Aminoadipic acid	NO	1	1.31	C6H11NO4	level1
5-Aminolevulinic acid	NO	1	1.09	C5H9NO3	level1
Aspartic acid	NO	1	1.14	C4H7NO4	level1
Car(3:0)_RT63	NO	1	1.00	C10H19NO4	level1
Dehydrolithocholic acid	NO	1	0.98	C24H38O3	level1
Indolelactic acid	NO	1	1.05	C11H11NO3	level1
Kynurenic acid	NO	1	1.07	C10H7NO3	level1
Mesaconic acid	NO	1	0.98	C5H6O4	level1
N1-Methyl-4-pyridone-3-carboxamide	NO	1	0.96	C7H8N2O2	level1
Tyrosine O-sulfate	NO	1	0.96	C9H11NO6S	level1
PC(39:6)_RT628	NO	1.68E-03	1.66	C47H82NO8P	level2
Arabinono-1,4-lactone	NO	3.23E-03	1.72	C5H8O5	level2
Cer(d18:1/16:0)	NO	9.89E-03	1.32	C34H67NO3	level2
Car(18:2)_RT502	NO	9.89E-03	0.80	C25H45NO4	level2
Car(13:0)_RT433	NO	1.78E-02	1.57	C20H39NO4	level2
Camphor	NO	2.56E-02	0.74	C10H16O	level2
2-Hydroxyhexanedioic acid	NO	2.56E-02	1.12	C6H10O5	level2
4-Hydroxyglutamic acid	NO	2.56E-02	1.27	C5H9NO5	level2
Car(7:0)_RT288	NO	4.74E-02	1.53	C14H27NO4	level2
Cysteine	NO	1.44E-01	0.78	C3H7NO2S	level2
Car(14:2)_RT416	NO	1.66E-01	1.51	C21H37NO4	level2
3-Methylhistidine	NO	6.22E-01	0.92	C7H11N3O2	level2

Car(15:0)_RT486	NO	6.22E-01	0.67	C22H43NO4	level2
Lactaldehyde	NO	6.22E-01	1.08	C3H6O2	level2
alpha-Muricholic acid	NO	1	1.00	C24H40O5	level2
beta-Muricholic acid	NO	1	1.00	C24H40O5	level2
Car(10:3-O)_RT335	NO	1	0.92	C17H27NO5	level2
Car(15:1-O)_RT438	NO	1	0.90	C22H41NO5	level2
Car(16:1-O2)_RT414	NO	1	1.09	C23H41NO6	level2
Car(5:1)_RT214	NO	1	1.02	C12H21NO4	level2
Car(9:1)_RT320	NO	1	1.00	C16H29NO4	level2
omega-Muricholic acid	NO	1	1.00	C24H40O5	level2
Ursocholic acid	NO	1	1.00	C24H40O5	level2
Sphingosyl-phosphocholine	NO	1.99E-04	0.77	C23H50N2O5P	level3
3beta,7alpha-Dihydroxy-5-cholestenoate	NO	2.85E-04	0.72	C27H44O4	level3
3alpha,7alpha-Dihydroxy-5beta-cholestanate	NO	3.74E-04	0.70	C27H46O4	level3
beta-Citryl-L-glutamate	NO	1.68E-03	0.64	C11H15NO10	level3
2,6-Dihydroxynicotinate	NO	3.23E-03	0.83	C6H5NO4	level3
4-(L-Alanin-3-yl)-2-hydroxy-cis,cis-muconate 6-semialdehyde	NO	9.89E-03	0.86	C9H11NO6	level3
4,5-seco-Dopa	NO	9.89E-03	0.86	C9H11NO6	level3
5-(L-Alanin-3-yl)-2-hydroxy-cis,cis-muconate 6-semialdehyde	NO	9.89E-03	0.86	C9H11NO6	level3
Betalamic acid	NO	9.89E-03	0.86	C9H9NO5	level3
Methyl cinnamate	NO	9.89E-03	0.68	C10H10O2	level3
N-Acetyl-L-glutamate	NO	9.89E-03	0.86	C7H11NO5	level3
Phosphocreatine	NO	1.70E-02	0.73	C4H10N3O5P	level3

(R)-4'-Phosphopantothenoyl-L-cysteine	NO	2.56E-02	1.22	C12H23N2O9PS	level3
18-Hydroxyoleate	NO	2.56E-02	1.24	C18H34O3	level3
2-Hydroxyhepta-2,4-dienedioate	NO	2.56E-02	0.89	C7H8O5	level3
2-Oxohept-3-enedioate	NO	2.56E-02	0.89	C7H8O5	level3
3-Dehydroshikimate	NO	2.56E-02	0.89	C7H8O5	level3
cis-9,10-Epoxystearic acid	NO	2.56E-02	1.24	C18H34O3	level3
Glycochenodeoxycholate 7-sulfate	NO	2.56E-02	0.83	C26H43NO8S	level3
Isonicotinylglycine	NO	2.56E-02	0.77	C8H8N2O3	level3
Nicotinate D-ribonucleoside	NO	2.56E-02	0.79	C11H14NO6	level3
Nicotinurate	NO	2.56E-02	0.77	C8H8N2O3	level3
3-Indoleacrylate	NO	3.41E-02	0.87	C11H9NO2	level3
4-(L-Alanin-3-yl)-2-hydroxy-cis,cis-muconate 6-semialdehyde	NO	4.74E-02	0.86	C9H11NO6	level3
4,5-seco-Dopa	NO	4.74E-02	0.86	C9H11NO6	level3
4-Amino-4-deoxychorismate	NO	4.74E-02	0.70	C10H11NO5	level3
4-Imidazolone-5-propanoate	NO	4.74E-02	1.30	C6H8N2O3	level3
5-(L-Alanin-3-yl)-2-hydroxy-cis,cis-muconate 6-semialdehyde	NO	4.74E-02	0.86	C9H11NO6	level3
Ethylenediaminetriacetic acid	NO	4.74E-02	1.30	C8H14N2O6	level3
L-Cystine	NO	4.74E-02	0.74	C6H12N2O4S2	level3
gamma-L-Glutamyl-L-cysteine	NO	7.72E-02	0.65	C8H14N2O5S	level3
N-Glucosylnicotinate	NO	9.71E-02	0.68	C12H16NO7	level3
18-Oxooleate	NO	1.03E-01	1.26	C18H32O3	level3
Acetyl-CoA	NO	1.03E-01	0.59	C23H38N7O17P3S	level3
Formylisoglutamine	NO	1.03E-01	1.22	C6H10N2O4	level3
Fumarate	NO	1.03E-01	1.40	C4H4O4	level3

L-Serine-phosphoethanolamine	NO	1.03E-01	1.23	C5H13N2O6P	level3
Maleic acid	NO	1.03E-01	1.40	C4H4O4	level3
3-(4-Hydroxyphenyl)pyruvate	NO	1.44E-01	1.38	C9H8O4	level3
5-Ureido-4-imidazole carboxylate	NO	1.44E-01	0.87	C5H6N4O3	level3
alpha-D-Galactose	NO	1.44E-01	1.07	C6H12O6	level3
Aspirin	NO	1.44E-01	1.38	C9H8O4	level3
beta-D-Glucose	NO	1.44E-01	1.07	C6H12O6	level3
D-Alanyl-D-serine	NO	1.44E-01	0.83	C6H12N2O4	level3
D-Fructose	NO	1.44E-01	1.07	C6H12O6	level3
D-Galactose	NO	1.44E-01	1.07	C6H12O6	level3
D-Glucose	NO	1.44E-01	1.07	C6H12O6	level3
Dopamine quinone	NO	1.44E-01	0.90	C8H9NO2	level3
Ethylenediamine-N,N'-diacetic acid	NO	1.44E-01	0.83	C6H12N2O4	level3
Isoniazid alpha-ketoglutaric acid	NO	1.44E-01	0.91	C11H11N3O5	level3
L-Mimosine	NO	1.44E-01	1.14	C8H10N2O4	level3
N5-Hydroxy-L-ornithine	NO	1.44E-01	0.64	C5H12N2O3	level3
16-Oxopalmitate	NO	1.66E-01	1.15	C16H30O3	level3
L-Alanyl-D-glutamate	NO	1.66E-01	0.83	C8H14N2O5	level3
(4R,5S)-4,5,6-Trihydroxy-2,3-dioxohexanoate	NO	2.11E-01	0.86	C6H8O7	level3
2-Dehydro-3-deoxy-D-glucarate	NO	2.11E-01	0.86	C6H8O7	level3
2-Hydroxyethylenedicarboxylate	NO	2.11E-01	0.86	C4H4O5	level3
4-Hydroxy-2-quinolone	NO	2.11E-01	1.12	C9H7NO2	level3
5-Dehydro-4-deoxy-D-glucarate	NO	2.11E-01	0.86	C6H8O7	level3
Carboxymethyloxysuccinate	NO	2.11E-01	0.86	C6H8O7	level3
D-Galactaro-1,4-lactone	NO	2.11E-01	0.86	C6H8O7	level3

D-Galactaro-1,5-lactone	NO	2.11E-01	0.86	C6H8O7	level3
D-Glucaro-1,4-lactone	NO	2.11E-01	0.86	C6H8O7	level3
D-Glucaro-1,5-lactone	NO	2.11E-01	0.86	C6H8O7	level3
D-Glutamine	NO	2.11E-01	1.19	C5H10N2O3	level3
Isoglutamine	NO	2.11E-01	1.19	C5H10N2O3	level3
Oxaloacetate	NO	2.11E-01	0.86	C4H4O5	level3
Indole-3-carboxylate	NO	3.07E-01	0.91	C9H7NO2	level3
(3Z)-Phytochromobilin	NO	6.22E-01	0.94	C33H36N4O6	level3
(R)-10-Hydroxystearate	NO	6.22E-01	1.06	C18H36O3	level3
15,16-Dihydrobiliverdin	NO	6.22E-01	0.94	C33H36N4O6	level3
3-Chloro-D-alanine	NO	6.22E-01	1.15	C3H6ClNO2	level3
3-Chloro-L-alanine	NO	6.22E-01	1.15	C3H6ClNO2	level3
3-Indoleacrylate	NO	6.22E-01	0.93	C11H9NO2	level3
4-Imidazolone-5-propanoate	NO	6.22E-01	3.34	C6H8N2O3	level3
5'-S-Methyl-5'-thioinosine	NO	6.22E-01	0.94	C11H14N4O4S	level3
9,10-Epoxy-18-hydroxystearate	NO	6.22E-01	0.94	C18H34O4	level3
Acetyl-maltose	NO	6.22E-01	0.97	C14H24O12	level3
D-Galacturonate	NO	1	0.94	C6H10O7	level3
D-Octopine	NO	1	0.94	C9H18N4O4	level3
16-Oxopalmitate	NO	1	0.92	C16H30O3	level3
16-Oxopalmitate	NO	1	1.15	C16H30O3	level3
18-Hydroxyoleate	NO	1	0.90	C18H34O3	level3
1-Aminocyclopropane-1-carboxylate	NO	1	1.04	C4H7NO2	level3
1-Pyrroline-4-hydroxy-2-carboxylate	NO	1	1.05	C5H7NO3	level3
3-D-Glucuronosyl-N2,6-disulfo-beta-D-glucosamine	NO	1	0.98	C12H21NO17S2	level3

4-Oxoproline	NO	1	1.05	C5H7NO3	level3
5-Oxo-D-proline	NO	1	1.05	C5H7NO3	level3
9,10-Dihydroxystearate	NO	1	0.90	C18H36O4	level3
CDP-choline	NO	1	1.05	C14H26N4O11P2	level3
cis-9,10-Epoxystearic acid	NO	1	0.90	C18H34O3	level3
cis-9,10-Epoxystearic acid	NO	1	0.84	C18H34O3	level3
D-beta-Phenylalanine	NO	1	0.91	C9H11NO2	level3
D-Phenylalanine	NO	1	0.91	C9H11NO2	level3
L-1-Pyrroline-3-hydroxy-5-carboxylate	NO	1	1.05	C5H7NO3	level3
L-beta-Phenylalanine	NO	1	0.91	C9H11NO2	level3
N-Succinyl-LL-2,6-diaminoheptanedioate	NO	1	1.30	C11H18N2O7	level3
Porphobilinogen	NO	1	0.98	C10H14N2O4	level3

VIP: variable importance on projection.

FDR P.value: P.value of FDR-adjusted Wilcoxon–Mann–Whitney test

FC: fold change of BD / SCZ

.

Supplementary Table 5C: Detailed information of the disorder-disorder differentially expressed metabolites selected from respective union-set of differentially expressed metabolites from the disorder-HC comparisons.(MDD-BD from MDD-HC & BD-HC)

Name	FDR P.value	FC	Formula	Confidence_level
2-Hydroxyoctanoic acid	6.22E-02	1.48	C8H16O3	level1
2-Aminooctanoic acid	1.23E-01	1.43	C8H17NO2	level1
3-methylcytidine	1.28E-01	1.27	C10H15N3O5	level1
Urocanic acid	1.28E-01	1.22	C6H6N2O2	level1
5-Aminopentanoic acid	2.69E-01	1.09	C5H11NO2	level1
Allolithocholic acid	2.69E-01	1.14	C24H40O3	level1
Arginine	2.69E-01	1.16	C6H14N4O2	level1
Aspartic acid	2.69E-01	1.46	C4H7NO4	level1
Car(12:2)_RT382	2.69E-01	1.26	C19H33NO4	level1
gamma-Glutamylalanine	2.69E-01	1.17	C8H14N2O5	level1
gamma-Glutamylvaline	2.69E-01	1.11	C10H18N2O5	level1
Lithocholic acid	2.69E-01	1.14	C24H40O3	level1
Nordeoxycholic acid	2.69E-01	1.26	C23H38O4	level1
Phenylpyruvic acid	2.69E-01	1.16	C9H8O3	level1
Tyrosine	2.69E-01	1.07	C9H11NO3	level1
2-Ketocaproic acid	2.79E-01	1.07	C6H10O3	level1
3-Methyl-2-oxovaleric acid	2.79E-01	1.07	C6H10O3	level1
Car(12:1-O)_RT364	2.79E-01	1.37	C19H35NO5	level1
Car(16:2-O)_RT395	2.79E-01	1.19	C23H41NO5	level1
Chenodeoxycholic acid	2.79E-01	0.76	C24H40O4	level1
cis-8,11,14-Eicosatrienoic acid	2.79E-01	0.87	C20H34O2	level1
Cysteic acid	2.79E-01	0.87	C3H7NO5S	level1
Deoxycholic acid	2.79E-01	0.76	C24H40O4	level1

Eicosapentaenoic acid	2.79E-01	0.85	C20H30O2	level1
Histidinol	2.79E-01	1.07	C6H11N3O	level1
Indolelactic acid	2.79E-01	1.06	C11H11NO3	level1
Ketoleucine	2.79E-01	1.07	C6H10O3	level1
Kynurenic acid	2.79E-01	1.12	C10H7NO3	level1
Mesaconic acid	2.79E-01	1.16	C5H6O4	level1
Salicylic acid	2.79E-01	1.00	C7H6O3	level1
3alpha-Hydroxy-12-oxo-5beta-cholan-24-oic acid	2.97E-01	1.04	C24H38O4	level1
3alpha-Hydroxy-6-oxo-5alpha-cholan-24-oic acid	2.97E-01	1.04	C24H38O4	level1
Apocholic acid	2.97E-01	1.04	C24H38O4	level1
Dehydrolithocholic acid	2.97E-01	1.05	C24H38O3	level1
Docosahexaenoic acid (DHA)	2.97E-01	0.89	C22H32O2	level1
Glucuronic acid	2.97E-01	0.90	C6H10O7	level1
Malic acid	2.97E-01	1.11	C4H6O5	level1
Pyruvic acid	2.97E-01	0.85	C3H4O3	level1
Xanthine	2.97E-01	0.83	C5H4N4O2	level1
4-Vinylphenol	5.94E-01	1.20	C8H8O	level1
Adenosine 3',5'-cyclic phosphate (cAMP)	5.94E-01	1.08	C10H12N5O6P	level1
Car(14:0)_RT467	5.94E-01	0.79	C21H41NO4	level1
Tyrosine O-sulfate	5.94E-01	1.05	C9H11NO6S	level1
5-Aminolevulinic acid	1	0.89	C5H9NO3	level1
Acetoin	1	0.95	C4H8O2	level1
Cortisone/Aldosterone	1	1.07	C21H28O5	level1

Arachidonic acid (AA)	1	1.01	C20H32O2	level1
Biliverdin	1	0.80	C33H34N4O6	level1
Capric acid	1	0.81	C10H20O2	level1
Car(10:0)_RT370	1	0.70	C17H33NO4	level1
Car(10:1)_RT357	1	0.91	C17H31NO4	level1
Car(11:0)_RT390	1	0.81	C18H35NO4	level1
Car(11:1)_RT383	1	1.00	C18H33NO4	level1
Car(12:0)_RT430	1	0.78	C19H37NO4	level1
Car(12:1)_RT402	1	0.92	C19H35NO4	level1
Car(14:1)_RT438	1	0.79	C21H39NO4	level1
Car(14:1)_RT447	1	0.79	C21H39NO4	level1
Car(14:1-O)_RT416	1	1.03	C21H39NO5	level1
Car(14:2-O)_RT389	1	1.06	C21H37NO5	level1
Car(16:1-O)_RT453	1	0.90	C23H43NO5	level1
Car(16:3)_RT442	1	0.86	C23H39NO4	level1
Car(16:4)_RT421	1	0.70	C23H37NO4	level1
Car(3:0)_RT63	1	1.03	C10H19NO4	level1
Car(6:0)_RT250	1	0.69	C13H25NO4	level1
Car(6:0)_RT263	1	0.69	C13H25NO4	level1
Car(8:0)_RT316	1	0.68	C15H29NO4	level1
Car(9:0)_RT346	1	0.85	C16H31NO4	level1
Cortisone/Aldosterone	1	1.07	C21H28O5	level1
Erucamide	1	0.87	C22H43NO	level1
gamma-Glutamyllysine	1	1.05	C11H21N3O5	level1
Homoarginine	1	1.03	C7H16N4O2	level1
Isovalerylcarnitine	1	1.07	C12H23NO4	level1

Kynurenine	1	1.02	C10H12N2O3	level1
Lactic acid	1	0.85	C3H6O3	level1
Lysine	1	1.03	C6H14N2O2	level1
Methionine	1	1.03	C5H11NO2S	level1
N1-Methyl-4-pyridone-3-carboxamide	1	1.05	C7H8N2O2	level1
Xanthurenic acid	1	1.03	C10H7NO4	level1
Car(11:1-O2)_RT289	6.22E-02	0.54	C18H31NO6	level2
Car(12:1-O2)_RT315	1.28E-01	0.57	C19H33NO6	level2
alpha-Muricholic acid	2.69E-01	1.15	C24H40O5	level2
beta-Muricholic acid	2.69E-01	1.15	C24H40O5	level2
Car(10:3-O)_RT335	2.69E-01	1.52	C17H27NO5	level2
omega-Muricholic acid	2.69E-01	1.15	C24H40O5	level2
Ursocholic acid	2.69E-01	1.15	C24H40O5	level2
Car(13:0)_RT433	2.79E-01	0.82	C20H39NO4	level2
Car(16:1-O2)_RT414	2.79E-01	1.12	C23H41NO6	level2
Car(9:1)_RT320	5.14E-01	1.09	C16H29NO4	level2
Car(5:1)_RT214	5.94E-01	1.10	C12H21NO4	level2
Lactaldehyde	5.94E-01	1.04	C3H6O2	level2
3-Methylhistidine	1	1.28	C7H11N3O2	level2
4-Hydroxyglutamic acid	1	0.99	C5H9NO5	level2
Car(14:2)_RT416	1	0.87	C21H37NO4	level2
Car(15:1-O)_RT438	1	1.10	C22H41NO5	level2
Car(7:0)_RT288	1	0.78	C14H27NO4	level2
Cysteine	1	1.04	C3H7NO2S	level2
D-Galacturonate	1.28E-01	1.23	C6H10O7	level3
gamma-L-Glutamyl-L-cysteine	1.28E-01	1.31	C8H14N2O5S	level3

Isoniazid alpha-ketoglutaric acid	1.28E-01	0.68	C11H11N3O5	level3
L-Serine-phosphoethanolamine	1.28E-01	0.80	C5H13N2O6P	level3
Methyl cinnamate	1.28E-01	0.59	C10H10O2	level3
Miraxanthin-V	1.28E-01	0.12	C17H18N2O6	level3
16-Oxopalmitate	2.69E-01	1.30	C16H30O3	level3
2,6-Dihydroxynicotinate	2.69E-01	1.07	C6H5NO4	level3
3-D-Glucuronosyl-N2,6-disulfo-beta-D-glucosamine	2.69E-01	1.30	C12H21NO17S2	level3
4-(L-Alanin-3-yl)-2-hydroxy-cis,cis-muconate 6-semialdehyde	2.69E-01	0.88	C9H11NO6	level3
4,5-seco-Dopa	2.69E-01	0.88	C9H11NO6	level3
5-(L-Alanin-3-yl)-2-hydroxy-cis,cis-muconate 6-semialdehyde	2.69E-01	0.88	C9H11NO6	level3
Acetyl-CoA	2.69E-01	1.45	C23H38N7O17P3S	level3
Dopamine quinone	2.69E-01	1.18	C8H9NO2	level3
Isonicotinylglycine	2.69E-01	1.33	C8H8N2O3	level3
L-Alanyl-D-glutamate	2.69E-01	1.36	C8H14N2O5	level3
N5-Hydroxy-L-ornithine	2.69E-01	1.62	C5H12N2O3	level3
N-Glucosylnicotinate	2.69E-01	1.52	C12H16NO7	level3
Nicotinurate	2.69E-01	1.33	C8H8N2O3	level3
(4R,5S)-4,5,6-Trihydroxy-2,3-dioxohexanoate	2.79E-01	1.12	C6H8O7	level3
1-Pyrroline-4-hydroxy-2-carboxylate	2.79E-01	0.87	C5H7NO3	level3
2-Dehydro-3-deoxy-D-glucarate	2.79E-01	1.12	C6H8O7	level3
2-Hydroxyethylenedicarboxylate	2.79E-01	1.12	C4H4O5	level3
4-Oxoproline	2.79E-01	0.87	C5H7NO3	level3

5-Dehydro-4-deoxy-D-glucarate	2.79E-01	1.12	C6H8O7	level3
5-Oxo-D-proline	2.79E-01	0.87	C5H7NO3	level3
5'-S-Methyl-5'-thioinosine	2.79E-01	1.12	C11H14N4O4S	level3
5-Ureido-4-imidazole carboxylate	2.79E-01	1.08	C5H6N4O3	level3
9,10-Epoxy-18-hydroxystearate	2.79E-01	1.09	C18H34O4	level3
Carboxymethyloxysuccinate	2.79E-01	1.12	C6H8O7	level3
CDP-choline	2.79E-01	0.87	C14H26N4O11P2	level3
D-beta-Phenylalanine	2.79E-01	1.23	C9H11NO2	level3
D-Galactaro-1,4-lactone	2.79E-01	1.12	C6H8O7	level3
D-Galactaro-1,5-lactone	2.79E-01	1.12	C6H8O7	level3
D-Glucaro-1,4-lactone	2.79E-01	1.12	C6H8O7	level3
D-Glucaro-1,5-lactone	2.79E-01	1.12	C6H8O7	level3
D-Glutamine	2.79E-01	0.86	C5H10N2O3	level3
D-Phenylalanine	2.79E-01	1.23	C9H11NO2	level3
Formylisoglutamine	2.79E-01	0.90	C6H10N2O4	level3
Isoglutamine	2.79E-01	0.86	C5H10N2O3	level3
L-1-Pyrroline-3-hydroxy-5-carboxylate	2.79E-01	0.87	C5H7NO3	level3
L-beta-Phenylalanine	2.79E-01	1.23	C9H11NO2	level3
L-Mimosine	2.79E-01	0.90	C8H10N2O4	level3
Oxaloacetate	2.79E-01	1.12	C4H4O5	level3
4-Amino-4-deoxychorismate	2.97E-01	1.12	C10H11NO5	level3
4-Imidazolone-5-propanoate	2.97E-01	0.90	C6H8N2O3	level3
beta-Citryl-L-glutamate	2.97E-01	1.12	C11H15NO10	level3
Ethylenediaminetriacetic acid	2.97E-01	0.90	C8H14N2O6	level3
Glycochenodeoxycholate 7-sulfate	2.97E-01	0.97	C26H43NO8S	level3
18-Hydroxyoleate	5.14E-01	0.94	C18H34O3	level3

9,10-Dihydroxystearate	5.14E-01	0.94	C18H36O4	level3
cis-9,10-Epoxystearic acid	5.14E-01	0.94	C18H34O3	level3
cis-9,10-Epoxystearic acid	5.94E-01	1.17	C18H34O3	level3
D-Alanyl-D-serine	5.94E-01	1.13	C6H12N2O4	level3
Ethylenediamine-N,N'-diacetic acid	5.94E-01	1.13	C6H12N2O4	level3
Porphobilinogen	5.94E-01	1.06	C10H14N2O4	level3
(3Z)-Phytochromobilin	1	0.80	C33H36N4O6	level3
(R)-10-Hydroxystearate	1	1.00	C18H36O3	level3
15,16-Dihydrobiliverdin	1	0.80	C33H36N4O6	level3
16-Oxopalmitate	1	0.92	C16H30O3	level3
18-Hydroxyoleate	1	0.96	C18H34O3	level3
18-Oxooleate	1	0.94	C18H32O3	level3
3-(4-Hydroxyphenyl)pyruvate	1	1.07	C9H8O4	level3
3-Indoleacrylate	1	0.99	C11H9NO2	level3
3-Indoleacrylate	1	1.00	C11H9NO2	level3
4-Hydroxy-2-quinolone	1	1.02	C9H7NO2	level3
Acetyl-maltose	1	1.06	C14H24O12	level3
Aspirin	1	1.07	C9H8O4	level3
cis-9,10-Epoxystearic acid	1	0.96	C18H34O3	level3
D-Octopine	1	1.26	C9H18N4O4	level3
Indole-3-carboxylate	1	1.19	C9H7NO2	level3
L-Cystine	1	1.07	C6H12N2O4S2	level3
Phosphocreatine	1	1.07	C4H10N3O5P	level3

VIP: variable importance on projection.

FDR P.value: P.value of FDR-adjusted Wilcoxon–Mann–Whitney test

FC: fold change of MDD / BD
