# Supplementary material for: Identifying plasma metabolic characteristics of major depressive disorder, bipolar disorder, and schizophrenia in adolescents
Source: Transl Psychiatry. 2024 Mar 26;14:163. doi: 10.1038/s41398-024-02886-z (PMC10966062; doi:10.1038/s41398-024-02886-z)
Supplement: Supplementary file 12 — Supplementary Table 6 [file 41398_2024_2886_MOESM12_ESM.pdf]

Supplementary Table 6A:The detailed parameters of LASSO and PCR models. (MDD-HC)

Name	model1	model2	model3	model4	model5	model6	model7	model8	model9	model10	PCR model	Confidence_level
Lambda.1se	0.04957	0.05971	0.05971	0.05971	0.07893	0.04957	0.05440	0.07893	0.06553	0.04517		
Intercept	-5.34E+00	-4.67E+00	-4.67E+00	-4.67E+00	-3.82E+00	-5.34E+00	-5.00E+00	-3.82E+00	-4.36E+00	-5.68E+00	0.07981	
5-Aminolevulinic acid	1.29E-08	9.76E-09	9.76E-09	9.76E-09	5.10E-09	1.29E-08	1.13E-08	5.10E-09	8.25E-09	1.47E-08	3.94E-09	level1
Acetoin	-3.60E-09	-2.76E-09	-2.76E-09	-2.76E-09	-1.29E-09	-3.60E-09	-3.20E-09	-1.29E-09	-2.23E-09	-4.05E-09	-4.45E-09	level1
Capric acid	1.63E-09	1.23E-09	1.23E-09	1.23E-09	4.97E-10	1.63E-09	1.45E-09	4.97E-10	9.73E-10	1.79E-09	9.83E-10	level1
Car(11:1)_RT383	2.38E-08	2.39E-08	2.39E-08	2.39E-08	2.28E-08	2.38E-08	2.37E-08	2.28E-08	2.40E-08	2.41E-08	8.05E-09	level1
Car(9:0)_RT346	1.02E-07	8.91E-08	8.91E-08	8.91E-08	7.47E-08	1.02E-07	9.56E-08	7.47E-08	8.35E-08	1.08E-07	3.33E-08	level1
Pyruvic acid	-2.17E-10	-1.78E-10	-1.78E-10	-1.78E-10	-1.01E-10	-2.17E-10	-1.99E-10	-1.01E-10	-1.54E-10	-2.35E-10	-8.12E-11	level1
Salicylic acid	1.16E-08	8.53E-09	8.53E-09	8.53E-09	3.79E-09	1.16E-08	1.01E-08	3.79E-09	6.98E-09	1.30E-08	6.50E-09	level1
Car(11:1-O2)_RT289	3.02E-08	2.67E-08	2.67E-08	2.67E-08	2.16E-08	3.02E-08	2.85E-08	2.16E-08	2.50E-08	3.18E-08		level2
Car(15:1-O)_RT438	2.70E-08	2.56E-08	2.56E-08	2.56E-08	2.41E-08	2.70E-08	2.62E-08	2.41E-08	2.51E-08	2.77E-08		level2
(R)-10-Hydroxystearate	1.04E-07	1.35E-07	1.35E-07	1.35E-07	1.27E-07	1.04E-07	1.23E-07	1.27E-07	1.41E-07	7.22E-08		level3
16-Oxopalmitate	-3.83E-08	-3.37E-08	-3.37E-08	-3.37E-08	-2.49E-08	-3.83E-08	-3.59E-08	-2.49E-08	-3.12E-08	-4.13E-08		level3
18-Hydroxyoleate	6.53E-23	1.45E-22	1.45E-22	1.45E-22	9.63E-23	6.53E-23	7.39E-23	9.63E-23	1.52E-22	1.11E-22		level3
cis-9,10-Epoxystearic acid	3.14E-08	2.40E-08	2.40E-08	2.40E-08	1.25E-08	3.14E-08	2.83E-08	1.25E-08	1.94E-08	3.21E-08		level3
3-Indoleacrylate	1.02E-07	9.39E-08	9.39E-08	9.39E-08	7.77E-08	1.02E-07	9.81E-08	7.77E-08	8.94E-08	1.07E-07		level3
3-methylcytidine	2.66E-07	1.36E-07	1.36E-07	1.36E-07	0	2.66E-07	2.04E-07	0	6.17E-08	3.25E-07		level1
Adenosine 3',5'-cyclic phosphate (cAMP)	0	0	0	0	0	0	0	0	0	0		level1
Arachidonic acid (AA)	0	0	0	0	0	0	0	0	0	0		level1
Arginine	0	0	0	0	0	0	0	0	0	0		level1
Biliverdin	0	0	0	0	0	0	0	0	0	0		level1
Car(10:0)_RT370	0	0	0	0	0	0	0	0	0	0		level1
Car(10:1)_RT357	0	0	0	0	0	0	0	0	0	0		level1

Car(11:0)_RT390	0	0	0	0	0	0	0	0	0	0	level1
Car(12:0)_RT430	0	0	0	0	0	0	0	0	0	0	level1
Car(12:1)_RT402	0	0	0	0	0	0	0	0	0	0	level1
Car(12:2)_RT382	0	0	0	0	0	0	0	0	0	0	level1
Car(14:0)_RT467	0	0	0	0	0	0	0	0	0	0	level1
Car(14:1)_RT438	0	0	0	0	0	0	0	0	0	0	level1
Car(14:1)_RT447	0	0	0	0	0	0	0	0	0	0	level1
Car(14:1-O)_RT416	0	0	0	0	0	0	0	0	0	0	level1
Car(14:2-O)_RT389	0	0	0	0	0	0	0	0	0	0	level1
Car(16:1-O)_RT453	0	0	0	0	0	0	0	0	0	0	level1
Car(16:2-O)_RT395	0	0	0	0	0	0	0	0	0	0	level1
Car(16:3)_RT442	0	0	0	0	0	0	0	0	0	0	level1
Car(16:4)_RT421	0	0	0	0	0	0	0	0	0	0	level1
Car(3:0)_RT63	0	0	0	0	0	0	0	0	0	0	level1
Car(6:0)_RT250	0	0	0	0	0	0	0	0	0	0	level1
Car(6:0)_RT263	0	0	0	0	0	0	0	0	0	0	level1
Car(8:0)_RT316	0	0	0	0	0	0	0	0	0	0	level1
Chenodeoxycholic acid	6.38E-14	0	0	0	0	6.38E-14	0	0	0	1.93E-11	level1
cis-8,11,14-Eicosatrienoic acid	0	0	0	0	0	0	0	0	0	0	level1
Cortisone/Aldosterone	1.65E-08	6.05E-09	6.05E-09	6.05E-09	0	1.65E-08	1.14E-08	0	8.35E-10	2.10E-08	level1
Cortisone/Aldosterone	1.16E-22	2.12E-22	2.12E-22	2.12E-22	0	1.16E-22	2.22E-22	0	0	1.83E-22	level1
Deoxycholic acid	1.94E-09	1.05E-09	1.05E-09	1.05E-09	0	1.94E-09	1.51E-09	0	5.66E-10	2.44E-09	level1
Docosahexaenoic acid (DHA)	0	1.33E-10	1.33E-10	1.33E-10	6.53E-10	0	0	6.53E-10	3.39E-10	0	level1
Eicosapentaenoic acid	0	0	0	0	0	0	0	0	0	0	level1
gamma-Glutamyllysine	0	0	0	0	0	0	0	0	0	0	level1
Histidinol	0	0	0	0	0	0	0	0	0	0	level1

Kynurenine	0	0	0	0	0	0	0	0	0	0	level1
Lysine	0	0	0	0	0	0	0	0	0	0	level1
Methionine	0	0	0	0	0	0	0	0	0	0	level1
N1-Methyl-4-pyridone-3-carboxamide	5.19E-09	9.87E-10	9.87E-10	9.87E-10	0	5.19E-09	3.14E-09	0	0	6.87E-09	level1
Xanthine	0	0	0	0	0	0	0	0	0	0	level1
4-Hydroxyglutamic acid	0	0	0	0	0	0	0	0	0	0	level2
Car(12:1-O2)_RT315	0	0	0	0	0	0	0	0	0	0	level2
Car(13:0)_RT433	0	0	0	0	0	0	0	0	0	0	level2
Car(14:2)_RT416	0	0	0	0	0	0	0	0	0	0	level2
Car(16:1-O2)_RT414	0	0	0	0	0	0	0	0	0	0	level2
Car(7:0)_RT288	0	0	0	0	0	0	0	0	0	0	level2
Cysteine	0	0	0	0	0	0	0	0	0	0	level2
(3Z)-Phytochromobilin	0	0	0	0	0	0	0	0	0	0	level3
15,16-Dihydrobiliverdin	0	0	0	0	0	0	0	0	0	0	level3
18-Oxooleate	1.20E-09	0	0	0	0	1.20E-09	0	0	0	1.03E-08	level3
3-Indoleacrylate	0	0	0	0	0	0	0	0	0	0	level3
5'-S-Methyl-5'-thioinosine	0	0	0	0	0	0	0	0	0	1.72E-09	level3
Acetyl-maltose	0	0	0	0	0	0	0	0	0	0	level3
beta-Citryl-L-glutamate	0	0	0	0	0	0	0	0	0	0	level3
Formylisoglutamine	0	0	0	0	0	0	0	0	0	0	level3
L-Cystine	0	0	0	0	0	0	0	0	0	0	level3
Miraxanthin-V	0	0	0	0	0	0	0	0	0	0	level3
Phosphocreatine	0	0	0	0	0	0	0	0	0	0	level3
Porphobilinogen	0	0	0	0	0	0	0	0	0	0	level3

The lambda.1se, intercept and coefficients of differentially expressed metabolites for LASSO models 1-10 of MDD-HC comparison.

The intercept and coefficients of biomarkers for PCR model of MDD-HC comparison.

Supplementary Table 6B:The detailed parameters of LASSO and PCR models. (BD-HC)

Name	model1	model2	model3	model4	model5	model6	model7	model8	model9	model10	PCR model	Confidence_level
Lambda.1se	0.07169	0.05681	0.10896	0.04298	0.04717	0.06532	0.04502	0.04717	0.04502	0.04717		
Intercept	-4.87E+00	-5.37E+00	-4.06E+00	-6.15E+00	-5.87E+00	-5.04E+00	-6.02E+00	-5.87E+00	-6.02E+00	-5.87E+00	-0.2668289	
3-methylcytidine	1.73E-06	1.78E-06	1.61E-06	1.89E-06	1.85E-06	1.74E-06	1.87E-06	1.85E-06	1.87E-06	1.85E-06	4.37E-07	level1
Acetoin	-6.17E-09	-8.16E-09	-1.36E-09	-1.09E-08	-9.94E-09	-6.97E-09	-1.04E-08	-9.94E-09	-1.04E-08	-9.94E-09	-4.59E-09	level1
Car(12:2)_RT382	2.86E-08	3.18E-08	1.63E-08	2.22E-08	2.57E-08	3.09E-08	2.37E-08	2.57E-08	2.37E-08	2.57E-08	1.61E-08	level1
Glucuronic acid	-1.65E-08	-1.51E-08	-4.23E-09	-1.31E-08	-1.38E-08	-1.62E-08	-1.33E-08	-1.38E-08	-1.33E-08	-1.38E-08	-2.17E-08	level1
Methionine	5.04E-09	7.58E-09	1.62E-09	9.06E-09	8.75E-09	6.05E-09	8.89E-09	8.75E-09	8.89E-09	8.75E-09	5.21E-09	level1
Nordeoxycholic acid	6.32E-08	7.33E-08	3.80E-08	8.22E-08	7.94E-08	6.78E-08	8.07E-08	7.94E-08	8.07E-08	7.94E-08	3.17E-08	level1
Cortisone/Aldosterone	5.86E-08	8.22E-08	2.36E-08	1.06E-07	9.81E-08	6.89E-08	1.02E-07	9.81E-08	1.02E-07	9.81E-08	2.39E-08	level1
Cortisone/Aldosterone	6.76E-23	2.75E-22	1.60E-22	1.88E-22	1.99E-22	7.18E-23	9.14E-23	1.99E-22	9.14E-23	1.99E-22		level1
Car(15:1-O)_RT438	2.40E-08	2.47E-08	2.38E-08	2.56E-08	2.54E-08	2.41E-08	2.56E-08	2.54E-08	2.56E-08	2.54E-08		level2
2,6-Dihydroxynicotinate	3.68E-07	3.93E-07	3.34E-07	4.31E-07	4.17E-07	3.79E-07	4.24E-07	4.17E-07	4.24E-07	4.17E-07		level3
4-(L-Alanin-3-yl)-2-hydroxy-cis,cis-muconate 6-semialdehyde	-2.89E-07	-3.88E-07	-2.79E-08	-4.58E-07	-4.43E-07	-3.35E-07	-4.53E-07	-4.43E-07	-4.53E-07	-4.43E-07		level3
2-Aminooctanoic acid	0	0	0	0	0	0	0	0	0	0		level1
2-Hydroxyoctanoic acid	0	0	0	0	0	0	0	0	0	0		level1
2-Ketocaproic acid	0	0	0	0	0	0	0	0	0	0		level1
3alpha-Hydroxy-12-oxo-5beta-cholan-24-oic acid	0	0	0	0	0	0	0	0	0	0		level1
3alpha-Hydroxy-6-oxo-5alpha-cholan-24-oic acid	0	0	0	0	0	0	0	0	0	0		level1
3-Methyl-2-oxovaleric acid	0	0	0	0	0	0	0	0	0	0		level1
4-Vinylphenol	4.61E-08	9.02E-08	0	1.37E-07	1.21E-07	6.44E-08	1.29E-07	1.21E-07	1.29E-07	1.21E-07		level1
5-Aminopentanoic acid	0	0	0	0	0	0	0	0	0	0		level1

Adenosine 3',5'-cyclic phosphate (cAMP)	0	0	0	0	0	0	0	0	0	0	level1
Allolithocholic acid	0	0	0	0	0	0	0	0	0	0	level1
Apocholic acid	0	0	0	0	0	0	0	0	0	0	level1
Arachidonic acid (AA)	0	0	0	0	0	0	0	0	0	0	level1
Arginine	0	0	0	0	0	0	0	0	0	0	level1
Aspartic acid	0	0	0	0	0	0	0	0	0	0	level1
Car(10:1)_RT357	0	0	0	0	0	0	0	0	0	0	level1
Car(11:0)_RT390	0	0	0	0	0	0	0	0	0	0	level1
Car(11:1)_RT383	3.71E-09	0	9.81E-09	0	0	1.53E-09	0	0	0	0	level1
Car(12:0)_RT430	0	0	0	0	0	0	0	0	0	0	level1
Car(12:1)_RT402	0	0	0	0	0	0	0	0	0	0	level1
Car(12:1-O)_RT364	0	0	0	1.87E-08	1.13E-08	0	1.51E-08	1.13E-08	1.51E-08	1.13E-08	level1
Car(14:1-O)_RT416	0	0	0	0	0	0	0	0	0	0	level1
Car(14:2-O)_RT389	0	0	0	0	0	0	0	0	0	0	level1
Car(16:1-O)_RT453	0	0	0	0	0	0	0	0	0	0	level1
Car(16:2-O)_RT395	0	0	0	0	0	0	0	0	0	0	level1
Car(16:3)_RT442	0	0	0	0	0	0	0	0	0	0	level1
Car(3:0)_RT63	0	0	0	0	0	0	0	0	0	0	level1
Car(9:0)_RT346	0	0	0	0	0	0	0	0	0	0	level1
Cysteic acid	0	0	0	0	0	0	0	0	0	0	level1
Dehydrolithocholic acid	0	0	0	0	0	0	0	0	0	0	level1
Docosahexaenoic acid (DHA)	0	0	0	0	0	0	0	0	0	0	level1
Erucamide	-1.24E-08	-2.76E-08	0	-4.57E-08	-3.96E-08	-1.83E-08	-4.27E-08	-3.96E-08	-4.27E-08	-3.96E-08	level1
gamma-Glutamylalanine	0	0	0	0	0	0	0	0	0	0	level1
gamma-Glutamyllysine	0	0	0	0	0	0	0	0	0	0	level1

gamma-Glutamylvaline	0	0	0	0	0	0	0	0	0	0	level1
Histidinol	0	0	0	0	0	0	0	0	0	0	level1
Homoarginine	0	0	0	0	0	0	0	0	0	0	level1
Indolelactic acid	0	0	0	0	0	0	0	0	0	0	level1
Isovalerylcarnitine	0	0	0	0	0	0	0	0	0	0	level1
Ketoleucine	0	0	0	0	0	0	0	0	0	0	level1
Kynurenic acid	0	0	0	0	0	0	0	0	0	0	level1
Kynurenine	0	0	0	0	0	0	0	0	0	0	level1
Lactic acid	0	0	0	0	0	0	0	0	0	0	level1
Lithocholic acid	0	0	0	0	0	0	0	0	0	0	level1
Lysine	0	0	0	0	0	0	0	0	0	0	level1
Malic acid	0	0	0	0	0	0	0	0	0	0	level1
Mesaconic acid	0	0	0	0	0	0	0	0	0	0	level1
N1-Methyl-4-pyridone-3-carboxamide	0	0	0	0	0	0	0	0	0	0	level1
Phenylpyruvic acid	0	0	0	0	0	0	0	0	0	0	level1
Salicylic acid	0	0	0	0	0	0	0	0	0	0	level1
Tyrosine	0	0	0	0	0	0	0	0	0	0	level1
Tyrosine O-sulfate	9.42E-09	2.68E-09	6.12E-09	0	0	7.3946E-09	0	0	0	0	level1
Urocanic acid	0	0	0	0	0	0	0	0	0	0	level1
Xanthurenic acid	0	0	0	0	0	0	0	0	0	0	level1
3-Methylhistidine	0	0	0	0	0	0	0	0	0	0	level2
4-Hydroxyglutamic acid	0	0	0	0	0	0	0	0	0	0	level2
alpha-Muricholic acid	0	0	0	0	0	0	0	0	0	0	level2
beta-Muricholic acid	0	0	0	0	0	0	0	0	0	0	level2

Car(10:3-O)_RT335	8.08E-08	1.51E-07	0	1.94E-07	1.82E-07	1.10E-07	1.88E-07	1.82E-07	1.88E-07	1.82E-07	level2
Car(16:1-O2)_RT414	0	0	0	0	0	0	0	0	0	0	level2
Car(5:1)_RT214	0	0	0	0	0	0	0	0	0	0	level2
Car(9:1)_RT320	0	0	0	0	0	0	0	0	0	0	level2
Cysteine	0	0	0	0	0	0	0	0	0	0	level2
Lactaldehyde	1.61E-08	2.10E-08	0	2.64E-08	2.46E-08	1.83E-08	2.55E-08	2.46E-08	2.55E-08	2.46E-08	level2
omega-Muricholic acid	0	0	0	0	0	0	0	0	0	0	level2
Ursocholic acid	0	0	0	0	0	0	0	0	0	0	level2
(3Z)-Phytochromobilin	0	0	0	0	0	0	0	0	0	0	level3
(4R,5S)-4,5,6-Trihydroxy-2,3-dioxohexanoate	0	4.85E-23	0	1.17E-22	1.10E-22	0	1.13E-22	1.10E-22	1.13E-22	1.10E-22	level3
(R)-10-Hydroxystearate	0	0	0	0	0	0	0	0	0	0	level3
15,16-Dihydrobiliverdin	0	0	0	0	0	0	0	0	0	0	level3
16-Oxopalmitate	0	0	0	-8.46024E-10	0	0	0	0	0	0	level3
16-Oxopalmitate	7.29E-08	1.24E-07	0	1.99E-07	1.75E-07	9.21E-08	1.88E-07	1.75E-07	1.88E-07	1.75E-07	level3
18-Hydroxyoleate	0	0	0	0	0	0	0	0	0	0	level3
18-Hydroxyoleate	0	0	0	0	0	0	0	0	0	0	level3
1-Pyrroline-4-hydroxy-2-carboxylate	0	0	0	0	0	0	0	0	0	0	level3
2-Dehydro-3-deoxy-D-glucarate	6.37E-08	1.08E-07	0	1.65E-07	1.47E-07	8.05E-08	1.56E-07	1.47E-07	1.56E-07	1.47E-07	level3
2-Hydroxyethylenedicarboxylate	1.50E-11	1.53E-10	0	2.31E-10	2.56E-10	1.54E-11	2.44E-10	2.56E-10	2.44E-10	2.56E-10	level3
3-(4-Hydroxyphenyl)pyruvate	0	0	0	0	0	0	0	0	0	0	level3
3-D-Glucuronosyl-N2,6-disulfo-beta-D-glucosamine	0	0	0	0	0	0	0	0	0	0	level3
3-Indoleacrylate	0	0	0	0	0	0	0	0	0	0	level3

3-Indoleacrylate	0	0	0	0	0	0	0	0	0	0	level3
4,5-seco-Dopa	-6.28E-21	-7.96E-21	0	-5.18E-21	-5.39E-21	-6.23E-21	-5.55E-21	-5.39E-21	-5.55E-21	-5.39E-21	level3
4-Amino-4-deoxychorismate	0	0	0	0	0	0	0	0	0	0	level3
4-Hydroxy-2-quinolone	0	0	0	0	0	0	0	0	0	0	level3
4-Imidazolone-5-propanoate	0	0	0	0	0	0	0	0	0	0	level3
4-Oxoproline	0	0	0	0	0	0	0	0	0	0	level3
5-(L-Alanin-3-yl)-2-hydroxy-cis,cis-muconate 6-semialdehyde	0	0	0	-1.06E-09	-3.90E-10	0.00E+00	-2.90E-10	-3.90E-10	-2.90E-10	-3.90E-10	level3
5-Dehydro-4-deoxy-D-glucarate	9.44E-12	3.31E-11	0	1.67E-11	3.48E-11	9.64E-12	3.41E-11	3.48E-11	3.41E-11	3.48E-11	level3
5-Oxo-D-proline	0	0	0	0	0	0	0	0	0	0	level3
5'-S-Methyl-5'-thioinosine	0	0	0	0	0	0	0	0	0	0	level3
5-Ureido-4-imidazole carboxylate	0	0	0	0	0	0	0	0	0	0	level3
9,10-Dihydroxystearate	-7.82E-09	-1.34E-08	0	-2.03E-08	-1.81E-08	-1.01E-08	-1.93E-08	-1.81E-08	-1.93E-08	-1.81E-08	level3
9,10-Epoxy-18-hydroxystearate	0	0	0	0	0	0	0	0	0	0	level3
Acetyl-CoA	0	0	0	0	0	0	0	0	0	0	level3
Acetyl-maltose	0	0	0	3.42E-09	1.46E-09	0	2.50E-09	1.46E-09	2.50E-09	1.46E-09	level3
Aspirin	0	0	0	0	0	0	0	0	0	0	level3
beta-Citryl-L-glutamate	0	0	0	0	0	0	0	0	0	0	level3
Carboxymethyloxysuccinate	0	6.44E-11	0	2.03E-10	2.89E-10	0	2.80E-10	2.89E-10	2.80E-10	2.89E-10	level3
CDP-choline	0	0	0	0	0	0	0	0	0	0	level3
cis-9,10-Epoxystearic acid	0.00E+00	1.84E-09	0	5.30E-09	4.33E-09	0.00E+00	4.91E-09	4.33E-09	4.91E-09	4.33E-09	level3
cis-9,10-Epoxystearic acid	0	0	0	-3.27E-12	-4.77E-12	0.00E+00	-4.56E-12	-4.77E-12	-4.56E-12	-4.77E-12	level3
cis-9,10-Epoxystearic acid	9.86E-08	1.60E-07	0	2.18E-07	1.98E-07	1.27E-07	2.08E-07	1.98E-07	2.08E-07	1.98E-07	level3
D-Alanyl-D-serine	0	0	0	0	0	0	0	0	0	0	level3
D-beta-Phenylalanine	0	0	0	0	0	0	0	0	0	0	level3
D-Galactaro-1,4-lactone	0	0	0	0	0	0	0	0	0	0	level3

D-Galactaro-1,5-lactone	0	0	0	0	0	0	0	0	0	0	level3
D-Galacturonate	0	0	0	0	0	0	0	0	0	0	level3
D-Glucaro-1,4-lactone	0	0	0	0	0	0	0	0	0	0	level3
D-Glucaro-1,5-lactone	0	0	0	0	0	0	0	0	0	0	level3
D-Glutamine	0	0	0	0	0	0	0	0	0	0	level3
D-Octopine	0	0	0	0	0	0	0	0	0	0	level3
Dopamine quinone	0	0	0	0	0	0	0	0	0	0	level3
D-Phenylalanine	0	0	0	0	0	0	0	0	0	0	level3
Ethylenediamine-N,N'-diacetic acid	0	0	0	0	0	0	0	0	0	0	level3
Ethylenediaminetriacetic acid	0	0	0	0	0	0	0	0	0	0	level3
Formylisoglutamine	0	0	0	0	0	0	0	0	0	0	level3
gamma-L-Glutamyl-L-cysteine	0	0	0	0	0	0	0	0	0	0	level3
Glycochenodeoxycholate 7-sulfate	0	0	0	0	0	0	0	0	0	0	level3
Indole-3-carboxylate	0	0	0	0	0	0	0	0	0	0	level3
Isoglutamine	0	0	0	0	0	0	0	0	0	0	level3
Isoniazid alpha-ketoglutaric acid	0	0	0	0	0	0	0	0	0	0	level3
Isonicotinylglycine	0	0	0	0	0	0	0	0	0	0	level3
L-1-Pyrroline-3-hydroxy-5-carboxylate	0	0	0	0	0	0	0	0	0	0	level3
L-Alanyl-D-glutamate	0	0	0	0	0	0	0	0	0	0	level3
L-beta-Phenylalanine	0	0	0	0	0	0	0	0	0	0	level3
L-Cystine	0	0	0	0	0	0	0	0	0	0	level3
L-Mimosine	-1.48E-07	-3.08E-07	0	-4.13E-07	-3.82E-07	-2.25E-07	-3.98E-07	-3.82E-07	-3.98E-07	-3.82E-07	level3
L-Serine-phosphoethanolamine	0	0	0	0	0	0	0	0	0	0	level3
Methyl cinnamate	0	0	0	0	0	0	0	0	0	0	level3

N5-Hydroxy-L-ornithine	0	0	0	0	0	0	0	0	0	0	level3
N-Glucosylnicotinate	0	0	0	0	0	0	0	0	0	0	level3
Nicotinurate	0	0	0	0	0	0	0	0	0	0	level3
Oxaloacetate	0	9.25E-11	0	9.61E-12	4.80E-11	0	5.25E-11	4.80E-11	5.25E-11	4.80E-11	level3
Phosphocreatine	0	0	0	0	0	0	0	0	0	0	level3
Porphobilinogen	0	0	0	0	0	0	0	0	0	0	level3

The lambda.1se, intercept and coefficients of differentially expressed metabolites for LASSO models 1-10 of BD-HC comparison.

The intercept and coefficients of biomarkers for PCR model of BD-HC comparison.

.

Supplementary Table 6C:The detailed parameters of LASSO and PCR models. (SCZ-HC)

Name	model1	model2	model3	model4	model5	model6	model7	model8	model9	model10	PCR model	Confidence_level
Lambda.1se	0.03281	0.05224	0.07580	0.03772	0.09130	0.03437	0.04544	0.06007	0.03132	0.04987		
Intercept	-2.64E-01	-7.61E-01	-1.41E+00	-3.87E-01	-1.57E+00	-3.04E-01	-5.87E-01	-9.65E-01	-2.27E-01	-7.01E-01	0.75386	
3-methylcytidine	-4.95E-07	-4.17E-07	-3.23E-07	-4.90E-07	-2.28E-07	-4.94E-07	-4.52E-07	-3.82E-07	-4.97E-07	-4.29E-07	-3.17E-07	level1
Adenosine 3',5'-cyclic phosphate (cAMP)	-7.44E-09	-3.77E-08	-3.43E-08	-2.54E-08	-1.38E-08	-1.35E-08	-3.34E-08	-4.03E-08	-1.50E-09	-3.62E-08	-4.68E-08	level1
Car(11:1)_RT383	-9.30E-09	-6.77E-09	-4.18E-09	-8.58E-09	-2.01E-09	-9.06E-09	-7.53E-09	-5.99E-09	-9.54E-09	-7.02E-09	-4.21E-09	level1
Car(16:2-O)_RT395	-7.75E-08	-5.31E-08	-4.57E-08	-7.16E-08	-4.47E-08	-7.55E-08	-6.05E-08	-4.97E-08	-7.97E-08	-5.54E-08	-2.60E-08	level1
Car(9:0)_RT346	-2.07E-07	-1.75E-07	-1.45E-07	-1.97E-07	-1.20E-07	-2.03E-07	-1.84E-07	-1.66E-07	-2.11E-07	-1.78E-07	-2.97E-08	level1
Pyroglutamic acid	3.82E-09	3.25E-09	2.81E-09	3.55E-09	2.56E-09	3.73E-09	3.36E-09	3.13E-09	3.92E-09	3.28E-09	7.57E-10	level1
Xanthurenic acid	-7.21E-07	-6.97E-07	-6.65E-07	-7.12E-07	-5.95E-07	-7.18E-07	-7.01E-07	-6.97E-07	-7.25E-07	-6.98E-07	-2.71E-07	level1
Car(16:1-O2)_RT414	-2.78E-07	-2.75E-07	-2.69E-07	-2.68E-07	-2.71E-07	-2.75E-07	-2.71E-07	-2.70E-07	-2.81E-07	-2.74E-07		level2
Isoniazid alpha-ketoglutaric acid	1.98E-07	1.87E-07	1.80E-07	1.98E-07	1.80E-07	1.98E-07	1.92E-07	1.83E-07	1.99E-07	1.89E-07		level3
Methyl cinnamate	5.42E-08	4.76E-08	3.95E-08	5.19E-08	3.19E-08	5.34E-08	4.93E-08	4.58E-08	5.49E-08	4.82E-08		level3
18-Hydroxyoleate	-6.53E-11	-8.82E-23	-3.42E-23	-9.21E-12	-1.00E-23	-4.07E-11	-1.38E-22	-4.01E-12	-9.91E-11	-9.12E-23		level3
4-(L-Alanin-3-yl)-2-hydroxy-cis,cis-muconate 6-semialdehyde	1.29E-06	1.31E-06	1.33E-06	1.30E-06	1.19E-06	1.30E-06	1.30E-06	1.33E-06	1.29E-06	1.30E-06		level3
cis-9,10-Epoxystearic acid	-5.41E-08	-3.77E-08	-2.51E-08	-4.88E-08	-1.79E-08	-5.23E-08	-4.25E-08	-3.28E-08	-5.58E-08	-3.93E-08		level3
2-Aminoadipic acid	0	0	0	0	0	0	0	0	0	0		level1
4-Methylsalicylic acid	0	0	0	0	0	0	0	0	0	0		level1
4-Vinylphenol	0	0	0	0	0	0	0	0	0	0		level1
5-Aminolevulinic acid	0	0	0	0	0	0	0	0	0	0		level1
Acetylcarnitine (Car(2:0))	0	0	0	0	0	0	0	0	0	0		level1
Arachidonic acid (AA)	0	0	0	0	0	0	0	0	0	0		level1
Arginine	0	0	0	0	0	0	0	0	0	0		level1

Aspartic acid	-5.79E-09	0	0	-1.13E-09	0	-4.22E-09	0	0	-7.29E-09	0	level1
Butyrylcarnitine	0	0	0	0	0	0	0	0	0	0	level1
Capric acid	-1.66E-09	-8.12E-10	-1.37E-10	-1.35E-09	0	-1.56E-09	-1.03E-09	-5.89E-10	-1.77E-09	-8.86E-10	level1
Car(10:0)_RT370	0	0	0	0	0	0	0	0	0	0	level1
Car(10:1)_RT357	0	0	0	0	0	0	0	0	0	0	level1
Car(10:2)_RT333	0	0	0	0	0	0	0	0	0	0	level1
Car(10:2)_RT347	0	0	0	0	0	0	0	0	0	0	level1
Car(11:0)_RT390	0	0	0	0	0	0	0	0	0	0	level1
Car(12:0)_RT430	0	0	0	0	0	0	0	0	0	0	level1
Car(12:1)_RT402	0	0	0	0	0	0	0	0	0	0	level1
Car(12:1-O)_RT364	0	0	0	0	0	0	0	0	0	0	level1
Car(12:2)_RT382	0	0	0	0	0	0	0	0	0	0	level1
Car(12:2-O)_RT340	0	0	0	0	0	0	0	0	0	0	level1
Car(14:0)_RT467	0	0	0	0	0	0	0	0	0	0	level1
Car(14:1)_RT438	0	0	0	0	0	0	0	0	0	0	level1
Car(14:1)_RT447	0	0	0	0	0	0	0	0	0	0	level1
Car(14:1-O)_RT416	0	0	0	0	0	0	0	0	0	0	level1
Car(14:2-O)_RT389	0	0	0	0	0	0	0	0	0	0	level1
Car(16:1-O)_RT453	0	0	0	0	0	0	0	0	0	0	level1
Car(16:2)_RT456	0	0	0	0	0	0	0	0	0	0	level1
Car(16:3)_RT442	0	0	0	0	0	0	0	0	0	0	level1
Car(16:4)_RT421	0	0	0	0	0	0	0	0	0	0	level1
Car(18:1)	0	0	0	0	0	0	0	0	0	0	level1
Car(3:0)_RT63	0	0	0	0	0	0	0	0	0	0	level1
Car(4:0)_RT100	0	0	0	0	0	0	0	0	0	0	level1
Car(6:0)_RT250	0	0	0	0	0	0	0	0	0	0	level1

Car(6:0)_RT263	0	0	0	0	0	0	0	0	0	0	level1
Car(8:0)_RT316	0	0	0	0	0	0	0	0	0	0	level1
cis-8,11,14-Eicosatrienoic acid	0	0	0	0	0	0	0	0	0	0	level1
Cortisone/Aldosterone	0	0	0	0	0	0	0	0	0	0	level1
Cortisone/Aldosterone	0	0	0	0	0	0	0	0	0	0	level1
Dehydrolithocholic acid	0	0	0	0	0	0	0	0	0	0	level1
Dihydroxyacetone	0	0	0	0	0	0	0	0	0	0	level1
Dimethylglycine	0	0	0	0	0	0	0	0	0	0	level1
Docosahexaenoic acid (DHA)	0	0	0	0	0	0	0	0	0	0	level1
Dodecanoic acid	0	0	0	0	0	0	0	0	-2.10E-12	0	level1
Eicosapentaenoic acid	0	0	0	0	0	0	0	0	0	0	level1
Epinephrine	1.12E-08	4.68E-09	1.52E-10	9.41E-09	0	1.06E-08	6.75E-09	2.58E-09	1.17E-08	5.38E-09	level1
Glutamic acid	0	0	0	0	0	0	0	0	0	0	level1
Glyceraldehyde	0	0	0	0	0	0	0	0	0	0	level1
Glycerophosphocholine	0	0	0	0	0	0	0	0	0	0	level1
Heptadecanoic acid	0	0	0	0	0	0	0	0	0	0	level1
Histamine	0	0	0	0	0	0	0	0	0	0	level1
Indole-3-pyruvic acid	0	0	0	0	0	0	0	0	0	0	level1
Indolelactic acid	0	0	0	0	0	0	0	0	0	0	level1
Isovalerylcarnitine	0	0	0	0	0	0	0	0	0	0	level1
Kynurenic acid	0	0	0	0	0	0	0	0	0	0	level1
Kynurenine	0	0	0	0	0	0	0	0	0	0	level1
Lactic acid	0	0	0	0	0	0	0	0	0	0	level1
Myristic acid	0	0	0	0	0	0	0	0	0	0	level1
N1-Methyl-4-pyridone-3-carboxamide	0	0	0	0	0	0	0	0	0	0	level1

Nordeoxycholic acid	0	0	0	0	0	0	0	0	0	0	level1
Normetanephrine	0	2.65E-11	0	2.14E-12	0	0	1.98E-11	3.32E-11	0	2.41E-11	level1
Ornithine	0	0	0	0	0	0	0	0	0	0	level1
PC(35:4)_RT617	0	0	0	0	0	0	0	0	0	0	level1
PC(36:3)_RT642	0	0	0	0	0	0	0	0	0	0	level1
PC(37:6)_RT608	0	0	0	0	0	0	0	0	0	0	level1
PC(40:6)_RT642	0	0	0	0	0	0	0	0	0	0	level1
PC(40:7)_RT616	0	0	0	0	0	0	0	0	0	0	level1
PC(42:8)_RT615	0	0	0	0	0	0	0	0	0	0	level1
Pentadecanoic acid	0	0	0	0	0	0	0	0	0	0	level1
Phenylpyruvic acid	0	0	0	0	0	0	0	0	0	0	level1
Pristanic acid	0	0	0	0	0	0	0	0	0	0	level1
Pyruvic acid	0	0	0	0	0	0	0	0	0	0	level1
S1P(d18:0)	0	0	0	0	0	0	0	0	0	0	level1
S1P(d18:1)	0	0	0	0	0	0	0	0	0	0	level1
S1P(d18:2)_RT463	0	0	0	0	0	0	0	0	0	0	level1
Stearamide	0	0	0	0	0	0	0	0	-2.70E-10	0	level1
Traumatic acid	0	0	0	0	0	0	0	0	0	0	level1
Tyrosine	0	0	0	0	0	0	0	0	0	0	level1
Tyrosine O-sulfate	-4.39E-08	-1.86E-08	0	-3.50E-08	0	-4.09E-08	-2.55E-08	-1.18E-08	-4.68E-08	-2.09E-08	level1
Undecanoic acid	0	0	0	0	0	0	0	0	0	0	level1
2-Hydroxyhexanedioic acid	0	0	0	0	0	0	0	0	0	0	level2
Arabinono-1,4-lactone	-1.68E-07	-1.06E-07	-3.14E-08	-1.52E-07	0	-1.63E-07	-1.27E-07	-8.33E-08	-1.73E-07	-1.13E-07	level2
Camphor	1.41E-08	4.70E-09	0	1.13E-08	0	1.32E-08	7.52E-09	1.88E-09	1.51E-08	5.65E-09	level2
Car(13:0)_RT433	0	0	0	0	0	0	0	0	0	0	level2
Car(14:2)_RT416	0	0	0	0	0	0	0	0	0	0	level2

Car(15:0)_RT486	0	0	0	0	0	0	0	0	0	0	level2
Car(15:1-O)_RT438	-5.68E-09	-2.59E-09	0	-5.19E-09	0	-5.53E-09	-3.82E-09	-1.34E-09	-5.83E-09	-3.01E-09	level2
Car(18:2)_RT502	0	0	0	0	0	0	0	0	0	0	level2
Car(5:1)_RT214	0	0	0	0	0	0	0	0	0	0	level2
Car(7:0)_RT288	0	0	0	0	0	0	0	0	0	0	level2
Car(9:1)_RT320	0	0	0	0	0	0	0	0	0	0	level2
Cer(d18:1/16:0)	0	0	0	0	0	0	0	0	0	0	level2
Lactaldehyde	0	0	0	0	0	0	0	0	0	0	level2
PC(39:6)_RT628	0	0	0	0	0	0	0	0	0	0	level2
(R)-10-Hydroxystearate	0	0	0	0	0	0	0	0	0	0	level3
(R)-4'-Phosphopantothenoyl-L-cysteine	0	0	0	0	0	0	0	0	0	0	level3
16-Oxopalmitate	0	0	0	0	0	0	0	0	0	0	level3
16-Oxopalmitate	0	0	0	0	0	0	0	0	0	0	level3
16-Oxopalmitate	0	0	0	0	0	0	0	0	0	0	level3
18-Hydroxyoleate	0	0	0	0	0	0	0	0	0	0	level3
18-Oxooleate	0	0	0	0	0	0	0	0	0	0	level3
1-Aminocyclopropane-1-carboxylate	0	0	0	0	0	0	0	0	0	0	level3
2-Hydroxyhepta-2,4-dienedioate	0	0	0	0	0	0	0	0	0	0	level3
2-Oxohept-3-enedioate	0	0	0	0	0	0	0	0	0	0	level3
3-(4-Hydroxyphenyl)pyruvate	-2.89E-10	0	0	-4.18E-11	0	-1.82E-10	0	-2.58E-11	-4.35E-10	0	level3
3alpha,7alpha-Dihydroxy-5beta-cholestanate	0	0	0	0	0	0	0	0	0	0	level3
3beta,7alpha-Dihydroxy-5-cholestenoate	0	0	0	0	0	0	0	0	0	0	level3

3-Chloro-D-alanine	0	0	0	0	0	0	0	0	0	0	level3
3-Chloro-L-alanine	0	0	0	0	0	0	0	0	0	0	level3
3-Dehydroshikimate	0	0	0	0	0	0	0	0	0	0	level3
3-D-Glucuronosyl-N2,6-disulfo-beta-D-glucosamine	0	0	0	0	0	0	0	0	0	0	level3
4-(L-Alanin-3-yl)-2-hydroxy-cis,cis-muconate 6-semialdehyde	0	0	0	0	0	0	0	0	0	0	level3
4,5-seco-Dopa	0	0	0	0	0	0	0	0	0	0	level3
4,5-seco-Dopa	5.68E-22	1.53E-21	5.64179E-22	0.00E+00	0	1.64E-21	4.31E-22	0.00E+00	5.91E-22	0.00E+00	level3
4-Hydroxy-2-quinolone	0	0	0	0	0	0	0	0	0	0	level3
4-Imidazolone-5-propanoate	-4.62E-10	-2.29E-10	0	-5.36E-10	0	-4.88E-10	-3.88E-10	-5.31E-11	-4.36E-10	-2.84E-10	level3
5-(L-Alanin-3-yl)-2-hydroxy-cis,cis-muconate 6-semialdehyde	0	0	0	0	0	0	0	4.22E-10	0	0	level3
5-(L-Alanin-3-yl)-2-hydroxy-cis,cis-muconate 6-semialdehyde	0	0	0	0	0	0	0	0	0	0	level3
5'-S-Methyl-5'-thioinosine	0	0	0	0	0	0	0	0	0	0	level3
9,10-Dihydroxystearate	4.84E-09	1.09E-09	0	4.22E-09	0	4.64E-09	2.56E-09	0	5.06E-09	1.59E-09	level3
Acetyl-maltose	0	0	0	0	0	0	0	0	0	0	level3
Aspirin	-4.62E-10	-7.76E-10	0	-7.70E-10	0	-5.89E-10	-8.09E-10	-7.00E-10	-2.98E-10	-7.88E-10	level3
Betalamic acid	0	0	0	0	0	0	0	0	0	0	level3
CDP-choline	0	0	0	0	0	0	0	0	0	0	level3
cis-9,10-Epoxystearic acid	0	0	0	0	0	0	0	0	0	0	level3
D-Galacturonate	0	0	0	0	0	0	0	0	0	0	level3
D-Glucose	0	0	0	0	0	0	0	0	0	0	level3
D-Glucose/alpha-D-Galactose	0	0	0	0	0	0	0	0	0	0	level3

D-Glucose/beta-D-Glucose	0	0	0	0	0	0	0	0	0	0	level3
D-Glucose/D-Fructose	0	0	0	0	0	0	0	0	0	0	level3
D-Glucose/D-Galactose	0	0	0	0	0	0	0	0	0	0	level3
D-Octopine	-5.59E-07	-2.94E-07	0	-4.92E-07	0	-5.38E-07	-3.85E-07	-1.86E-07	-5.80E-07	-3.25E-07	level3
Fumarate	0	0	0	0	0	0	0	0	0	0	level3
Maleic acid	0	0	0	0	0	0	0	0	0	0	level3
N-Acetyl-L-glutamate	0	0	0	0	0	0	0	0	0	0	level3
Nicotinate D-ribonucleoside	0	0	0	0	0	0	0	0	0	0	level3
N-Succinyl-LL-2,6-diaminoheptanedioate	0	0	0	0	0	0	0	0	0	0	level3
Porphobilinogen	0	0	0	0	0	0	0	0	0	0	level3
Sphingosyl-phosphocholine	0	0	0	0	0	0	0	0	0	0	level3

The lambda.1se, intercept and coefficients of differentially expressed metabolites for LASSO models 1-10 of SCZ-HC comparison.

The intercept and coefficients of biomarkers for PCR model of SCZ-HC comparison.

.

Supplementary Table 6D:The detailed parameters of LASSO and PCR models. (MDD-SCZ)

	model1	model2	model3	model4	model5	model6	model7	model8	model9	model10	PCR model	Confidence_level
lambda.1se	0.04849	0.04849	0.05841	0.04419	0.05841	0.05322	0.04849	0.07721	0.07036	0.05322		
(Intercept)	-2.39E+00	-2.39E+00	-2.53E+00	-2.33E+00	-2.53E+00	-2.45E+00	-2.39E+00	-2.59E+00	-2.56E+00	-2.45E+00	0.3718999	
Phenylpyruvic acid	-2.64E-07	-2.64E-07	-2.18E-07	-2.86E-07	-2.18E-07	-2.42E-07	-2.64E-07	-1.45E-07	-1.71E-07	-2.42E-07	-8.70E-08	level1
Car(11:0)_RT390	-1.45E-07	-1.45E-07	-1.01E-07	-1.69E-07	-1.01E-07	-1.23E-07	-1.45E-07	-4.85E-08	-6.44E-08	-1.23E-07	-3.36E-08	level1
Car(16:2-O)_RT395	-1.99E-07	-1.99E-07	-1.58E-07	-2.18E-07	-1.58E-07	-1.79E-07	-1.99E-07	-8.95E-08	-1.13E-07	-1.79E-07	-3.14E-08	level1
Car(12:1-O)_RT364	-3.29E-08	-3.29E-08	-2.42E-08	-3.73E-08	-2.42E-08	-2.85E-08	-3.29E-08	-1.17E-08	-1.57E-08	-2.85E-08	-2.87E-08	level1
Car(12:0)_RT430	-2.33E-08	-2.33E-08	-2.00E-08	-2.49E-08	-2.00E-08	-2.16E-08	-2.33E-08	-1.63E-08	-1.76E-08	-2.16E-08	-4.49E-09	level1
Dodecanoic acid	-1.71E-09	-1.71E-09	-1.24E-09	-1.96E-09	-1.24E-09	-1.47E-09	-1.71E-09	-4.59E-10	-7.16E-10	-1.47E-09	-9.60E-10	level1
Pyroglutamic acid	2.19E-09	2.19E-09	2.46E-09	2.05E-09	2.46E-09	2.32E-09	2.19E-09	2.69E-09	2.62E-09	2.32E-09	7.91E-10	level1
Xanthine	5.76E-08	5.76E-08	4.90E-08	6.23E-08	4.90E-08	5.32E-08	5.76E-08	3.70E-08	4.08E-08	5.32E-08	1.39E-08	level1
4-Imidazolone-5-propanoate	-1.39E-09	-1.39E-09	-1.18E-09	-1.50E-09	-1.18E-09	-1.28E-09	-1.39E-09	-8.68E-10	-9.68E-10	-1.28E-09		level3
Methyl cinnamate	4.26E-08	4.26E-08	4.04E-08	4.42E-08	4.04E-08	4.14E-08	4.26E-08	3.42E-08	3.62E-08	4.14E-08		level3
Car(11:1-O2)_RT289	5.06E-08	5.06E-08	4.31E-08	5.45E-08	4.31E-08	4.68E-08	5.06E-08	3.23E-08	3.58E-08	4.68E-08		level2
beta-Citryl-L-glutamate	1.61E-07	1.61E-07	1.31E-07	1.76E-07	1.31E-07	1.46E-07	1.61E-07	8.26E-08	9.85E-08	1.46E-07		level3
Miraxanthin-V	8.59E-07	8.59E-07	6.99E-07	9.38E-07	6.99E-07	7.79E-07	8.59E-07	4.83E-07	5.54E-07	7.79E-07		level3
Traumatic acid	-3.98E-08	-3.98E-08	-2.75E-08	-4.37E-08	-2.75E-08	-3.42E-08	-3.98E-08	0	-1.06E-08	-3.42E-08		level1
D-Galactose	-2.10E-09	-2.10E-09	-1.24E-08	0	-1.24E-08	-7.34E-09	-2.10E-09	-2.05E-08	-2.23E-08	-7.34E-09		level3
D-Fructose	-1.22E-09	-1.22E-09	-1.08E-09	0	-1.08E-09	-1.02E-09	-1.22E-09	-7.64E-09	-4.86E-10	-1.02E-09		level3
PC(36:3)_RT642	-3.83E-10	-3.83E-10	-1.09E-11	-5.48E-10	-1.09E-11	-2.03E-10	-3.83E-10	0	0	-2.03E-10		level1
alpha-D-Galactose	-3.35E-10	-3.35E-10	-1.56E-12	0	-1.56E-12	-1.59E-10	-3.35E-10	-1.21E-22	0	-1.59E-10		level3
beta-D-Glucose	0	0	0	0	0	-7.55E-23	0	-3.63E-22	0	-7.55E-23		level3
D-Glucose	-1.60E-22	-1.60E-22	-2.15E-22	0	-2.15E-22	0	-1.60E-22	-4.83E-22	-2.56E-22	0		level3
4-Methylsalicylic acid	0	0	0	-1.03E-09	0	0	0	0	0	0		level1
3-methylcytidine	0	0	0	0	0	0	0	0	0	0		level1

4-Vinylphenol	0	0	0	0	0	0	0	0	0	0	level1
Acetoin	0	0	0	0	0	0	0	0	0	0	level1
Arachidonic acid (AA)	0	0	0	0	0	0	0	0	0	0	level1
Arginine	0	0	0	0	0	0	0	0	0	0	level1
Car(10:0)_RT370	0	0	0	0	0	0	0	0	0	0	level1
Car(12:2)_RT382	0	0	0	0	0	0	0	0	0	0	level1
Car(12:2-O)_RT340	0	0	0	0	0	0	0	0	0	0	level1
Car(14:1-O)_RT416	0	0	0	0	0	0	0	0	0	0	level1
Car(14:2-O)_RT389	0	0	0	0	0	0	0	0	0	0	level1
Car(18:1)	0	0	0	0	0	0	0	0	0	0	level1
Car(8:0)_RT316	0	0	0	0	0	0	0	0	0	0	level1
Car(9:0)_RT346	0	0	0	0	0	0	0	0	0	0	level1
Chenodeoxycholic acid	0	0	0	0	0	0	0	0	0	0	level1
Deoxycholic acid	0	0	0	0	0	0	0	0	0	0	level1
Dihydroxyacetone	0	0	0	0	0	0	0	0	0	0	level1
Dimethylglycine	0	0	0	0	0	0	0	0	0	0	level1
gamma-Glutamyllysine	0	0	0	0	0	0	0	0	0	0	level1
Glyceraldehyde	0	0	0	0	0	0	0	0	0	0	level1
Lactic acid	0	0	0	0	0	0	0	0	0	0	level1
Lysine	0	0	0	0	0	0	0	0	0	0	level1
PC(40:6)_RT642	0	0	0	0	0	0	0	0	0	0	level1
PC(42:8)_RT615	0	0	0	0	0	0	0	0	0	0	level1
Stearamide	0	0	0	0	0	0	0	0	0	0	level1
Xanthurenic acid	0	0	0	0	0	0	0	0	0	0	level1
2-Hydroxyhexanedioic acid	0	0	0	0	0	0	0	0	0	0	level2
4-Hydroxyglutamic acid	0	0	0	0	0	0	0	0	0	0	level2

Arabinono-1,4-lactone	0	0	0	0	0	0	0	0	0	0	level2
Car(12:1-O2)_RT315	0	0	0	0	0	0	0	0	0	0	level2
Car(18:2)_RT502	0	0	0	0	0	0	0	0	0	0	level2
Cer(d18:1/16:0)	0	0	0	0	0	0	0	0	0	0	level2
3-(4-Hydroxyphenyl)pyruvate	0	0	0	0	0	0	0	0	0	0	level3
3alpha,7alpha-Dihydroxy-5beta-cholestanate	0	0	0	0	0	0	0	0	0	0	level3
3beta,7alpha-Dihydroxy-5-cholestenoate	0	0	0	0	0	0	0	0	0	0	level3
4-(L-Alanin-3-yl)-2-hydroxy-cis,cis-muconate 6-semialdehyde	0	0	0	0	0	0	0	0	0	0	level3
4,5-seco-Dopa	0	0	0	0	0	0	0	0	0	0	level3
5-(L-Alanin-3-yl)-2-hydroxy-cis,cis-muconate 6-semialdehyde	0	0	0	0	0	0	0	0	0	0	level3
Aspirin	0	0	0	0	0	0	0	0	0	0	level3
Isoniazid alpha-ketoglutaric acid	0	0	0	0	0	0	0	0	0	0	level3
Nicotinate D-ribonucleoside	0	0	0	0	0	0	0	0	0	0	level3
Phosphocreatine	0	0	0	0	0	0	0	0	0	0	level3
Sphingosyl-phosphocholine	0	0	0	0	0	0	0	0	0	0	level3
Ornithine	1.27E-09	1.27E-09	1.48E-10	1.78E-09	1.48E-10	7.33E-10	1.27E-09	0	0	7.33E-10	level1

The lambda.1se, intercept and coefficients of differentially expressed metabolites for LASSO models 1-10 of MDD-SCZ comparison.

The intercept and coefficients of biomarkers for PCR model of MDD-SCZ comparison.

.

Supplementary Table 6E:The detailed parameters of LASSO and PCR models. (BD-SCZ)

	model1	model2	model3	model4	model5	model6	model7	model8	model9	model10	PCR model	Confidence_level
lambda.1se	0.05637	0.08975	0.04680	0.04680	0.03885	0.03885	0.05136	0.04680	0.04264	0.03885		
(Intercept)	2.22E+00	1.32E+00	2.64E+00	2.64E+00	3.09E+00	3.09E+00	2.43E+00	2.64E+00	2.87E+00	3.09E+00	6.60E-01	
Xanthurenic acid	-1.04E-06	-4.97E-08	-1.47E-06	-1.47E-06	-1.91E-06	-1.91E-06	-1.25E-06	-1.47E-06	-1.69E-06	-1.91E-06	-4.01E-07	level1
Car(9:0)_RT346	-7.32E-08	-2.19E-09	-1.06E-07	-1.06E-07	-1.41E-07	-1.41E-07	-8.91E-08	-1.06E-07	-1.23E-07	-1.41E-07	-3.73E-08	level1
Dodecanoic acid	-2.74E-09	-1.57E-09	-3.26E-09	-3.26E-09	-3.80E-09	-3.80E-09	-3.00E-09	-3.26E-09	-3.52E-09	-3.80E-09	-8.70E-10	level1
2-Aminooctanoic acid	1.07E-07	2.17E-08	1.46E-07	1.46E-07	1.86E-07	1.86E-07	1.26E-07	1.46E-07	1.66E-07	1.86E-07	5.31E-08	level1
Arabinono-1,4-lactone	-1.98E-07	-3.29E-07	-1.28E-07	-1.28E-07	-6.46E-08	-6.46E-08	-1.63E-07	-1.28E-07	-9.63E-08	-6.46E-08		level2
PC(39:6)_RT628	-3.44E-08	-4.94E-08	-2.58E-08	-2.58E-08	-1.65E-08	-1.65E-08	-3.02E-08	-2.58E-08	-2.13E-08	-1.65E-08		level2
Cer(d18:1/16:0)	-7.27E-08	-4.72E-08	-8.20E-08	-8.20E-08	-9.12E-08	-9.12E-08	-7.74E-08	-8.20E-08	-8.66E-08	-9.12E-08		level2
Camphor	1.26E-08	1.87E-09	1.67E-08	1.67E-08	2.09E-08	2.09E-08	1.46E-08	1.67E-08	1.88E-08	2.09E-08		level2
Phosphocreatine	1.30E-07	5.20E-08	1.56E-07	1.56E-07	1.74E-07	1.74E-07	1.43E-07	1.56E-07	1.66E-07	1.74E-07		level3
beta-Citryl-L-glutamate	1.26E-07	1.47E-07	1.03E-07	1.03E-07	8.30E-08	8.30E-08	1.15E-07	1.03E-07	9.27E-08	8.30E-08		level3
PC(40:7)_RT616	-4.65E-08	0	-6.83E-08	-6.83E-08	-9.11E-08	-9.11E-08	-5.72E-08	-6.83E-08	-7.95E-08	-9.11E-08		level1
Erucamide	-1.34E-08	0	-2.76E-08	-2.76E-08	-4.26E-08	-4.26E-08	-2.04E-08	-2.76E-08	-3.49E-08	-4.26E-08		level1
Acetoin	-1.08E-09	0	-1.34E-09	-1.34E-09	-1.32E-09	-1.32E-09	-1.24E-09	-1.34E-09	-1.36E-09	-1.32E-09		level1
PC(36:3)_RT642	-5.23E-10	0	-9.64E-10	-9.64E-10	-1.45E-09	-1.45E-09	-7.40E-10	-9.64E-10	-1.20E-09	-1.45E-09		level1
Car(13:0)_RT433	0	0	0	0	-2.08E-09	-2.08E-09	0	0	-9.10E-10	-2.08E-09		level2
Arachidonic acid (AA)	0	0	0	0	0	0	0	0	0	0		level1
Car(11:0)_RT390	0	0	0	0	0	0	0	0	0	0		level1
Car(12:0)_RT430	0	0	0	0	0	0	0	0	0	0		level1
Docosahexaenoic acid (DHA)	0	0	0	0	0	0	0	0	0	0		level1
Eicosapentaenoic acid	0	0	0	0	0	0	0	0	0	0		level1
gamma-Glutamylalanine	0	0	0	0	0	0	0	0	0	0		level1
gamma-Glutamyllysine	0	0	0	0	0	0	0	0	0	0		level1

Heptadecanoic acid	0	0	0	0	0	0	0	0	0	0	level1
Histidinol	0	0	0	0	0	0	0	0	0	0	level1
Lysine	0	0	0	0	0	0	0	0	0	0	level1
Malic acid	0	0	0	0	0	0	0	0	0	0	level1
Ornithine	0	0	0	0	0	0	0	0	0	0	level1
PC(35:4)_RT617	0	0	0	0	0	0	0	0	0	0	level1
PC(37:6)_RT608	0	0	0	0	0	0	0	0	0	0	level1
PC(40:6)_RT642	0	0	0	0	0	0	0	0	0	0	level1
Pristanic acid	0	0	0	0	0	0	0	0	0	0	level1
Salicylic acid	0	0	0	0	0	0	0	0	0	0	level1
Undecanoic acid	0	0	0	0	0	0	0	0	0	0	level1
4-Hydroxyglutamic acid	0	0	0	0	0	0	0	0	0	0	level2
Car(7:0)_RT288	0	0	0	0	0	0	0	0	0	0	level2
(R)-4'-Phosphopantothenoyl-L-cysteine	0	0	0	0	0	0	0	0	0	0	level3
18-Hydroxyoleate	0	0	0	0	0	0	0	0	0	0	level3
3alpha,7alpha-Dihydroxy-5beta-cholestanate	0	0	0	0	0	0	0	0	0	0	level3
3beta,7alpha-Dihydroxy-5-cholestenoate	0	0	0	0	0	0	0	0	0	0	level3
4-Imidazolone-5-propanoate	0	0	0	0	0	0	0	0	0	0	level3
cis-9,10-Epoxystearic acid	0	0	0	0	0	0	0	0	0	0	level3
Ethylenediaminetriacetic acid	0	0	0	0	0	0	0	0	0	0	level3
Isonicotinylglycine	0	0	0	0	0	0	0	0	0	0	level3
L-Cystine	0	0	0	0	0	0	0	0	0	0	level3
Methyl cinnamate	0	0	0	0	0	0	0	0	0	0	level3

Nicotinate D-ribonucleoside	0	0	0	0	0	0	0	0	0	0	level3
Nicotinurate	0	0	0	0	0	0	0	0	0	0	level3
Sphingosyl-phosphocholine	0	0	0	0	0	0	0	0	0	0	level3
4-Amino-4-deoxychorismate	0	0	5.00E-08	5.00E-08	1.06E-07	1.06E-07	2.35E-08	5.00E-08	7.75E-08	1.06E-07	level3

The lambda.1se, intercept and coefficients of differentially expressed metabolites for LASSO models 1-10 of BD-SCZ comparison.

The intercept and coefficients of biomarkers for PCR model of BD-SCZ comparison.
