# Supplementary material for: Identifying plasma metabolic characteristics of major depressive disorder, bipolar disorder, and schizophrenia in adolescents
Source: Transl Psychiatry. 2024 Mar 26;14:163. doi: 10.1038/s41398-024-02886-z (PMC10966062; doi:10.1038/s41398-024-02886-z)
Supplement: Supplementary file 13 — Supplementary Table 7 [file 41398_2024_2886_MOESM13_ESM.pdf]

Supplementary Table 7: Detailed information of medications in each disorder.

Diagnosis	Analysis ID	Drug 1	Drug 2	Drug 3	Drug 4
BD	E208	Venlafaxine	Ibuprofen and Codeine Phosphate Tablets		
BD	E209	Sertraline	Quetiapine		
BD	X123	Paroxetine	Valproic acid	Olanzapine	
BD	X130	Fluvoxamine	Clonazepam		
BD	X139	Sertraline	Aripiprazole	Benzhexol	Dexzopiclone
BD	X193	Fluoxetine	Aripiprazole	Huperzine A	
BD	X194	Sertraline			
BD	X201	Valproic acid	Aripiprazole	Amfebutamone	
BD	X228	Amisulpride	Benzhexol		
BD	X281	Sertraline			
BD	X327	Escitalopram	Agomelatine		
BD	X54_2	Other			
BD	X69	Valproic acid	Quetiapine		
BD	X71	Other			
BD	YJ142	Sertraline	Alprazolam	Aripiprazole	
BD	YJ204	Lamotrigine	Quetiapine		
BD	YJ250	lithium carbonate	Quetiapine		
BD	YJ257	Quetiapine			
BD	YJ265	Other			
BD	YJ270	lithium carbonate	Quetiapine	Amfebutamone	
BD	YJ288	Fluoxetine	Quetiapine		
BD	YJ357	Quetiapine	Tandospirone	Benzhexol	
BD	YJ358	Fluvoxamine	Paliperidone	Alprazolam	
BD	YJ371	Venlafaxine	Amisulpride	Olanzapine	Quetiapine

BD	YJ405	Escitalopram	Aripiprazole	Tandospirone	Lithium carbonate
BD	YJ414	Sertraline	Risperidone		
BD	YJ415	Sertraline			
BD	YJ65	Sertraline	Olanzapine		
BD	YJ79	Sertraline	Trazodone	Quetiapine	
BD	YJ93	Aripiprazole			
MDD	X124	Sertraline	Quetiapine	Alprazolam	Idebenone
MDD	X14	Sertraline	Aripiprazole		
MDD	X146	Duloxetine	Alprazolam	Benzhexol	
MDD	X159	Fluoxetine	Zolpidem	Mirtazapine	
MDD	X206	Venlafaxine	Olanzapine	Zopiclone	
MDD	X22	Sertraline	Buspirone		
MDD	X227	Sertraline			
MDD	X266	Sertraline			
MDD	X269	Venlafaxine	Olanzapine		
MDD	YJ124	Sertraline	Aripiprazole		
MDD	YJ172	Other			
MDD	YJ176	Sertraline	Quetiapine		
MDD	YJ233	Sertraline			
MDD	YJ261	Sertraline	Aripiprazole		
MDD	YJ279	Fluoxetine	Trazodone		
MDD	YJ289	Other			
MDD	YJ298	Trazodone			
MDD	YJ325	Sertraline	Aripiprazole	Benzhexol	Quetiapine
MDD	YJ329	Alprazolam			
MDD	YJ35	Escitalopram	Dexzopiclone		

MDD	YJ365	Sertraline	Aripiprazole	
MDD	YJ69	Sertraline	Olanzapine	
SCZ	X10	Sulpiride	Clozapine	
SCZ	X103	Amisulpride	Huperzine A	Loratadine
SCZ	X105	Other		
SCZ	X126	Hypnotics		
SCZ	X129	Other		
SCZ	X152	Fluoxetine	Olanzapine	
SCZ	X163	Olanzapine	Clonazepam	
SCZ	X185	Olanzapine	Paliperidone	
SCZ	X191	Amisulpride	Olanzapine	Benzhexol
SCZ	X192	Olanzapine	Valproic acid	
SCZ	X202	Amisulpride	Benzhexol	
SCZ	X235	Aripiprazole		
SCZ	X238	Sertraline		
SCZ	X242	Aripiprazole		
SCZ	X247	Fluoxetine	Olanzapine	Huperzine A
SCZ	X265	Olanzapine	Risperidone	Benzhexol
SCZ	X270	Olanzapine		
SCZ	X279	Olanzapine		
SCZ	X28	Amisulpride	Aripiprazole	
SCZ	X324	Olanzapine		
SCZ	X332	Olanzapine		
SCZ	X5	Olanzapine	Aripiprazole	Benzhexol
SCZ	X6	Aripiprazole	Citalopram Hydrobromide	
SCZ	X81	Aripiprazole		

SCZ	YJ133	Aripiprazole	
SCZ	YJ230	Olanzapine	
SCZ	YJ294	Sertraline	Olanzapine
