# Supplementary material for: Identifying plasma metabolic characteristics of major depressive disorder, bipolar disorder, and schizophrenia in adolescents
Source: Transl Psychiatry. 2024 Mar 26;14:163. doi: 10.1038/s41398-024-02886-z (PMC10966062; doi:10.1038/s41398-024-02886-z)
Supplement: Supplementary file 14 — Supplementary Table 8 [file 41398_2024_2886_MOESM14_ESM.pdf]

Supplementary Table 8. Dietary habit of patients with MDD, BD, SCZ and HCs.

	MDD (n=27/45)	BD (n=32/43)	SCZ (n=19/37)	HCs (n=26/50)	P-value
Starchy food (days/week)	6.5±1.0	6.6±1.2	7±0	6.8±1.0	0.102
Vegetable (days/week)	6.4±1.5	6.1±4.6	6.9±0.2	6.7±0.7	0.210
Meat (days/week)	6.6±0.9	6.6±1.3	6.9±0.3	6.8±0.5	0.210
Tea (days/week)	1.3±1.3	1.2±1.2	0.7±1.1	1.3±1.6	0.414
Coffee (days/week)	1.0±1.5	0.6±1.5	0.6±1.1	0.6±1.3	0.440
Fruit (days/week)	4.1±1.4	4.9±1.7	5.0±1.7	5.5±1.6	0.440

Continuous variables were presented as the mean ± SD(standard deviation).The differences in starchy food, vegetable, meat, tea, coffee, and fruit were analyzed using the Kruskal-Wallis test followed by FDR.

Abbreviation: MDD, major depressive disorder; BD, bipolar disorder; SCZ, schizophrenia; HC, health control;
